# A Precise Reproductive Calendar of Sexual and Apomictic Genotypes of *Eragrostis curvula*

**DOI:** 10.3390/plants15071050

**Published:** 2026-03-29

**Authors:** Juan Pablo Selva, Diego Zappacosta, Cristian Andrés Gallo, Petrus Bisp, Emidio Albertini, Viviana Echenique

**Affiliations:** 1 Centro de Recursos Naturales Renovables de la Zona Semiárida (CERZOS-CONICET-UNS), CCT CONICET Bahía Blanca, Bahía Blanca 8000, Argentina; jpselva@criba.edu.ar (J.P.S.); dczappa@criba.edu.ar (D.Z.); cgallo@cerzos-conicet.gob.ar (C.A.G.); petrusbisp@gmail.com (P.B.); 2 Departamento de Agronomía, Universidad Nacional del Sur (UNS), Bahía Blanca 8000, Argentina; 3 Dipartimento di Scienze Agrarie, Alimentari e Ambientali, Università degli Studi di Perugia, 06121 Perugia, Italy

**Keywords:** weeping lovegrass, apomixis, reproductive development, cytoembryology, morphometric analysis

## Abstract

*Eragrostis curvula* serves as a valuable model for studying diplosporous apomixis due to its unique reproductive mode, wide ploidy range, and extensive genomic resources. A major limitation for reproductive studies in this species is the difficulty of isolating female tissues at precise developmental stages, for example, for transcriptomics studies, since different floral tissues can introduce expression noise from non-target tissues. To overcome this, we performed a detailed cytoembryological and morphometric characterization of male and female development in seven *E. curvula* genotypes with different ploidy levels (2X–7X) and reproductive modes (sexual, facultative apomictic, and obligate apomictic). Using differential interference contrast microscopy and methyl salicylate clarification, we described key cytological stages of male and female development. These stages were then correlated with external floral parameters, including pistil, ovary, style, and anther length, to generate genotype-specific developmental calendars. Pistil length showed the strongest association with female developmental stage, particularly during the early phases of ovule development, enabling more precise staging. Synchrony between male and female development was also evaluated, revealing no consistent differences among reproductive modes or ploidy levels. This genotype-informed framework provides a practical tool for stage prediction and tissue selection, supporting future reproductive, developmental, and comparative studies in *E. curvula* and related grasses.

## 1. Introduction

*Eragrostis curvula* (weeping lovegrass) is a warm-season grass widely distributed across Southern Africa and other regions of the world, valued for its forage quality and adaptability. Despite notable genetic and phenotypic variation among genotypes, many accessions produce progenies that are highly uniform. Early studies initially attributed this uniformity to self-fertilization, but cytological analyses later demonstrated that the underlying mechanism is diplospory—a form of gametophytic apomixis in which the unreduced female gametophyte develops directly from the megaspore mother cell (MMC) without meiosis (apomeiosis), and the embryo arises by parthenogenesis [1,2,3]. Subsequent research revealed the coexistence of sexual, facultative apomictic, and obligate apomictic genotypes within a polyploid complex, with apomixis frequently associated with polyploidy [3,4].

Apomixis has attracted considerable attention due to its potential application in plant breeding, as it enables the fixation of elite genotypes and the maintenance of heterosis across generations [5]. Recent advances in genome engineering have demonstrated the feasibility of generating synthetic apomixis in crop species by manipulating key meiotic and embryogenic regulators [6,7,8,9,10,11]. However, the efficiency and stability of engineered systems remain lower than those observed in natural apomictic species, highlighting the need for a deeper understanding of the developmental and molecular mechanisms underlying natural apomixis.

*E. curvula* represents an excellent model for studying apomixis due to its distinctive reproductive features. This species exhibits a specific type of apomictic embryo sac development (*Eragrostis*-type), characterized by the absence of meiotic stages. A tetranucleated embryo sac is formed after two rounds of mitotic division of the megasporocyte, and the embryo:endosperm ploidy ratio remains constant in both sexual and apomictic seeds [12], in contrast to several other apomictic species where endosperm formation frequently involves altered maternal–paternal genomic contributions [13,14]. In addition, although the embryo develops parthenogenetically, endosperm formation still requires fertilization of the polar nucleus by a sperm cell, a process known as pseudogamy. In many pseudogamous apomictic species with octonucleate embryo sacs, fertilization of two unreduced polar nuclei by a reduced sperm cell results in an embryo:endosperm ploidy ratio of 2:5, instead of the 2:3 ratio observed in sexual reproduction. However, *E. curvula* develops a tetranucleate embryo sac containing a single unreduced polar nucleus; therefore, fertilization restores the canonical 2:3 embryo:endosperm ratio despite the apomictic origin of the embryo. As in most apomictic species, normal reduced microspores are produced by meiosis during pollen development, demonstrating that apomeiosis affects only female development [15].

Extensive efforts have therefore focused on identifying genes and regulatory networks associated with apomictic reproduction. In *E. curvula*, transcriptomic analyses, high-quality genome assemblies, and dense genetic maps have facilitated the identification of genomic regions and candidate genes linked to diplospory [16,17,18,19,20,21]. Nevertheless, reproductive and transcriptomic studies in *E. curvula* are limited by the small size of floral structures and the difficulty of isolating female tissues at precise developmental stages. Most previous transcriptomic analyses relied on whole spikelets [16,17,22], which introduce expression noise from anthers and other non-target tissues and may include multiple developmental stages within a single sample [23].

Reproductive calendars based on correlations between internal reproductive stages and external floral traits have been developed in several species, including *Brachiaria* [24], *Paspalum notatum* [25], and *Paspalum rufum* [26], as well as in crop species such as rice, where anther length has been correlated with stages of megagametogenesis [27], and in *Cenchrus americanus*, where pistil length has been used to stage female development [28].

Accurate characterization of gene expression dynamics during megasporogenesis and megagametogenesis requires precise developmental staging and tissue selection. Establishing correlations between internal gametophytic stages and external floral parameters is therefore essential to enable reliable stage prediction. Clarification of pistils with methyl salicylate [29] provides a practical, robust method for observing intact ovules and embryo sacs without sectioning artifacts, facilitating detailed cytoembryological characterization. Although reproductive modes and embryo sac development have been described in *Eragrostis* species of different ploidy levels [2,30,31,32,33], these studies did not systematically correlate external floral morphology with specific internal gametophytic stages across genotypes differing in ploidy and reproductive mode. The absence of such correlations limits the ability to design stage-specific sampling strategies for downstream molecular analyses.

Therefore, this study aimed to characterize male and female reproductive development in *E. curvula* and to establish correlations between internal cytological stages and external floral morphometric traits across genotypes differing in ploidy and reproductive mode. Based on these relationships, we developed a morphometric reproductive calendar that enables the prediction of female developmental stages and facilitates stage-specific sampling for downstream transcriptomic and functional studies of apomixis.

## 2. Results

### 2.1. Clarification Method for Analysis of the Reproductive Development of Weeping Lovegrass

Previous cytoembryological studies on *E. curvula* mainly used paraffin embedding, sectioning, and staining techniques. In this study, a clearing method was employed that allows quick processing of plant material and easier examination of cellular structures. This approach overcomes many limitations of traditional embedding and sectioning, which are labor-intensive and time-consuming.

**Figure 1:**
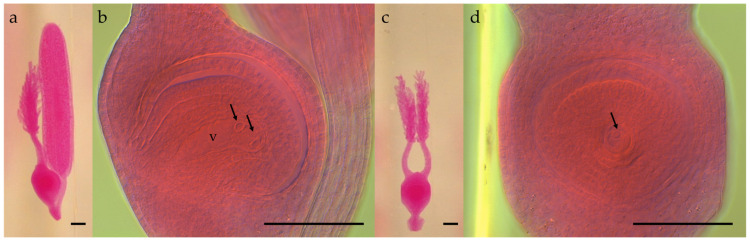
Clarified pistil of *E. curvula* observed under stereomicroscope and DIC microscopy. (**a**) Sagittal view of a clarified pistil under a stereomicroscope. (**b**) DIC micrograph of the ovule in sagittal orientation, showing the binucleated apomictic embryo sac with both micropylar nuclei (arrows) and the chalazal vacuole (v) visible in the same focal plane. (**c**) Frontal view of the same clarified pistil under a stereomicroscope. (**d**) DIC micrograph of the ovule in frontal orientation, showing a single micropylar nucleus (arrow). Scale bars = 100 µm.

A methyl salicylate-based clearing protocol enabled detailed observation of both ovule and anther structures without sectioning. The protocol provided clear visualization; however, special attention must be paid to the lateral positioning of the pistil during examination to ensure a sagittal view of the ovary (Figure 1).

### 2.2. Panicle and Spikelet Structure in Different E. curvula Genotypes

Seven genotypes differing in ploidy level and reproductive mode were analyzed (Table 1).

Panicle maturation in weeping lovegrass progressed from the top to the bottom, and along each branch, while within the spikelet, development followed a bottom-to-top pattern (Figure S1). The number of anthecia per spikelet varied across genotypes (Figure S2). The observed genotype-dependent trait was the abortion of anthecia at the spikelet apex. For example, some genotypes, such as OTA-S, exhibited only a few fully developed anthecia, with the upper part of the spikelet completely aborted (Figure S3).

### 2.3. Male Developmental Stages

**Figure 2:**
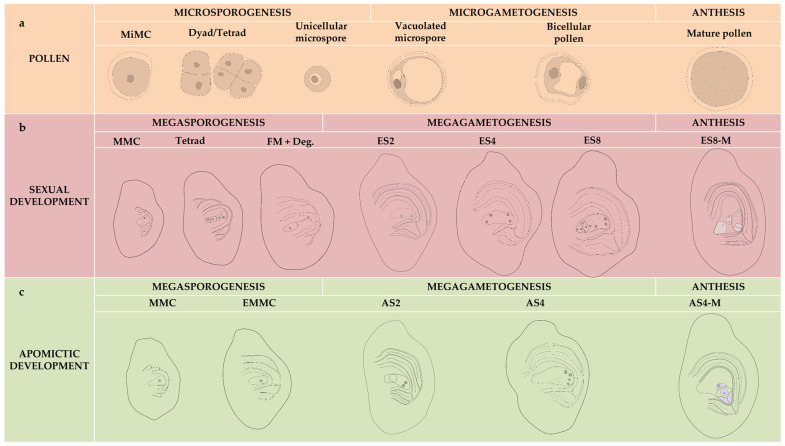
Scheme of male and female developmental stages. (**a**) The six male developmental stages: Microspore mother cell (MiMC), Dyad and Tetrads, Unicellular microspore, Vacuolated unicellular microspore, Bicellular pollen, and Mature pollen grain. (**b**) The seven sexual female developmental stages: Megaspore mother cell (MMC), Tetrad or triad just after meiosis, Functional and Degenerated megaspores (MF + Deg), Binucleated embryo sac just after the first mitosis (ES2), Tetranucleated (ES4), Octonucleated embryo sac (ES8) after the second and third mitosis, and Mature embryo sac at anthesis (ES8-M). (**c**) The five female apomictic developmental stages: Megaspore mother cell (MMC), Elongated megaspore mother cell (EMMC), Binucleated apomictic embryo sac after first mitosis (AS2), Tetranucleated embryo sac after the second mitosis (AS4), and Mature embryo sac at anthesis (AS4-M).

**Figure 3:**
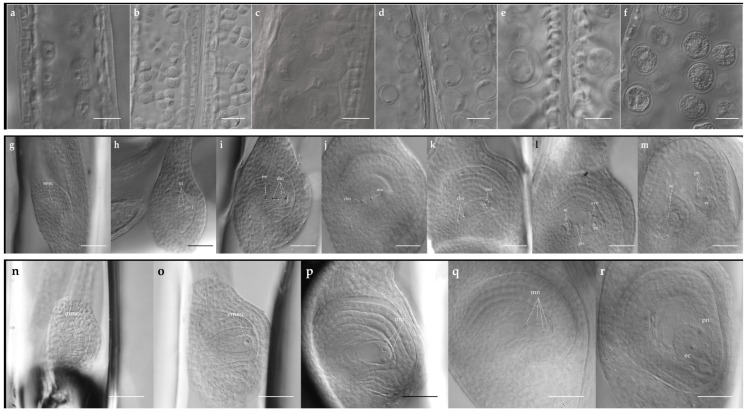
DIC micrographs of male and female reproductive development in *E. curvula*. (**a**) Microspore mother cells within the anther. (**b**) Meiosis: dyads and tetrads of haploid microspores. (**c**) Non-vacuolated unicellular microspore. (**d**) Vacuolated unicellular microspore. (**e**) Bicellular pollen grain after the first mitotic division. (**f**) Mature pollen grains at anthesis. (**g**) Megaspore mother cell (mmc) in the ovule. (**h**) Tetrad (tri) after meiosis of the MMC. (**i**) Functional megaspore (fm) and degenerating megaspores (dm). (**j**) Binucleated embryo sac showing chalazal (chn) and micropylar (mn) nuclei. (**k**) Tetranucleated embryo sac with two nuclei at each pole (chn, mn). (**l**) Octonucleated embryo sac showing antipodal cells (ac), egg cell (ec), and polar nuclei (pn) after cellularization. (**m**) Mature embryo sac with antipodal cells (ac), egg cell (ec), and polar nucleus (pn). (**n**) Megaspore mother cell (mmc) at the onset of apomictic development. (**o**) Elongated MMC (emmc) showing cell elongation and vacuolation without meiosis. (**p**) Binucleated apomictic embryo sac with both micropylar nuclei (mn). (**q**) Tetranucleated apomictic embryo sac with micropylar nuclei (mn). (**r**) Mature apomictic embryo sac showing egg cell (ec) and polar nucleus (pn). Scale bars, (**a**–**f**) = 20 µm, (**g**–**r**) = 50 µm.

Male reproductive development was documented in all evaluated genotypes and followed a conserved pattern (Figure 2a and Figure 3a–f).

The process began in the sporogenous tissue derived from the archeosporium, which underwent mitotic divisions to generate Microspore Mother Cells (MiMC, Figure 3a). These cells subsequently entered meiosis, forming dyads and then tetrads of haploid microspores (Figure 3b).

Male Reproductive Development Was Classified into Six Cytological Stages:

1. MiMC (Figure 3a): Presence of microspore mother cells within the anthers;
2. Meiosis (Figure 3b): Formation of dyads and tetrads;
3. Non-vacuolated unicellular microspore (1N NV, Figure 3c): Single-celled microspore lacking a visible vacuole;
4. Vacuolated unicellular microspore (1N V, Figure 3d): Formation of a prominent vacuole;
5. Bicellular pollen grain (2N, Figure 3e): First mitotic division of the microspore;
6. Mature pollen grain (MP, Figure 3f): Fully developed pollen at anthesis.

### 2.4. Female Sexual Developmental Stages

Seven distinct stages of female sexual development were identified (Figure 2b and Figure 3g–m):

1. Megaspore Mother Cell (MMC, Figure 3g): Enlarged trapezoidal cell in direct contact with the nucellar epidermis.
2. Dyad and Tetrad formation (Figure 3h): Meiosis of the MMC producing first a dyad, followed by a tetrad.
3. Functional Megaspore (FM, Figure 3i): A single chalazal megaspore remained functional, while the micropylar megaspores degenerated.
4. Binucleated Embryo Sac (ES2, Figure 3j): First mitotic division of the functional megaspore.
5. Tetranucleated Embryo Sac (ES4, Figure 3k): Second mitotic division resulting in four nuclei. Unlike its apomictic counterpart, two nuclei are positioned at each pole.
6. Octonucleated Embryo Sac (ES8, Figure 3l): Third mitotic division.
7. Mature Embryo Sac (ES8-M, Figure 3m): Cellularization and differentiation of a Polygonum-type embryo sac at anthesis.

Antipodal proliferation resulted in more than three antipodal cells in mature embryo sacs.

### 2.5. Female Apomictic Developmental Stages

Five distinct stages were identified during apomictic female development (Figure 2c and Figure 3n–r):

1. MMC (Figure 3n): Morphologically similar to that observed in sexual development.
2. Elongated MMC (EMMC, Figure 3o): Cell elongation and vacuolation without meiosis.
3. Binucleated Apomictic Embryo Sac (AS2, Figure 3p): First mitotic division; both nuclei remained at the micropylar end.
4. Tetranucleated Apomictic Embryo Sac (AS4, Figure 3q): Second mitotic division. In the apomictic *Eragrostis*-type embryo sac, this is the final mitotic division.
5. Mature Apomictic Embryo Sac (AS4-M, Figure 3r): Cellularization and maturation.

For the construction of the reproductive calendar, female development was grouped into four broader stages common to both reproductive modes:

- Stage I—MMC.
- Stage II—Postmeiosis, corresponding to the sexual stages of Dyad and Tetrad formation, as well as FM and the apomictic stage of EMMC.
- Stage III—Immature embryo sac, including the sexual stages ES2, ES4, and ES8, and the apomictic stages AS2 and AS4.
- Stage IV—Mature embryo sac, present in both sexual (ES8-M) and apomictic (AS4-M) processes, but with different characteristics.

### 2.6. Timeline of Male and Female Gametophytic Development

A comparative analysis of male and female reproductive development showed that both processes were synchronized across all evaluated genotypes, regardless of reproductive mode or ploidy level (Table 2). Meiosis occurred simultaneously in anthers and ovules; however, synchronization was more consistent at the early and late stages (female Stages I and II, and male Stages MiMC and mature pollen) than at the intermediate stages. When anthers were at the first stage, containing archesporial pollen cells, most ovaries (74%, 167 of 227 observations) also showed MMCs (Table 2). As microsporogenesis advanced and dyads/tetrads appeared in the anthers (meiosis), meiosis was also seen in the pistils, or the EMMC was forming (Stage II) in all genotypes. The percentage of pistils in Stages I and II varied among genotypes (Table 2), mainly due to differences in sample collection timing.

During microgametogenesis (Stages 1N and 2N) and megagametogenesis (Stages III and IV), the proportions of observed structures varied among genotypes, showing no clear patterns linked to reproductive mode or ploidy. This variation may result more from differences in sampling time rather than inherent developmental differences between genotypes. However, once anthers reached their final stage and contained mature pollen grains, all ovules had also advanced to Stage IV (Table 2).

### 2.7. Correlation Between Pistils and Anthers Measurements and Female Developmental Stages

Pistils and anthers were measured using a stereomicroscope and analyzed with differential interference contrast (DIC) microscopy to determine the corresponding stage of female development (Table 3 and Table S1).

**Figure 4:**
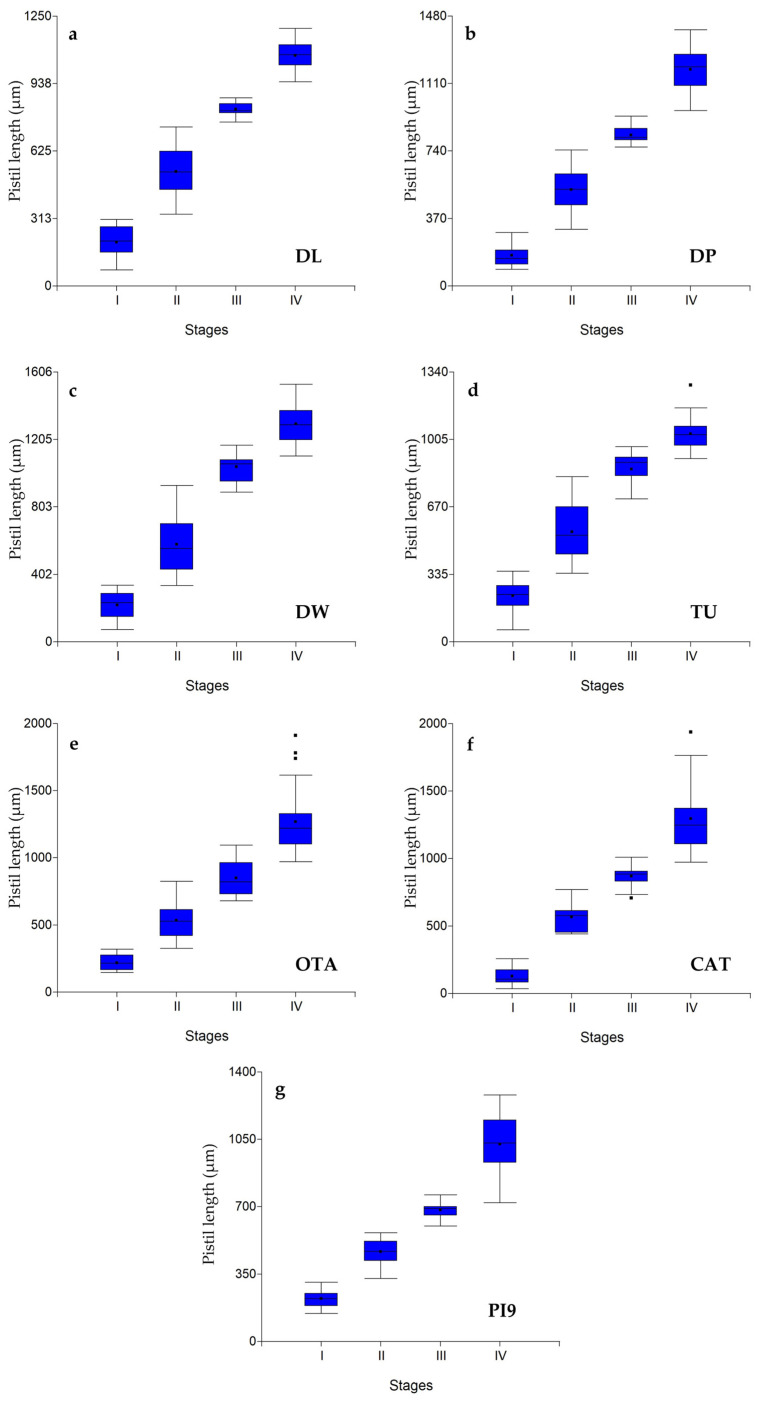
Box-plot of pistil length for all female developmental stages and *E. curvula* genotypes. (**a**) PI9: PI299920, (**b**) OTA: OTA-S, (**c**) TU: Tanganyika, (**d**) DW: Don Walter, (**e**) CAT: Catalina, (**f**) DL: Don Luis, (**g**) DP: Don Pablo. Female developmental stages: I: Megaspore mother cell, II: Postmeiosis or EMMC, III: Immature embryo sac, and IV: Mature embryo sac.

Mean values varied significantly between developmental stages for all comparisons within the same genotype and parameter, except for AL in DL and CAT (Table 3 and Table S2). Although these differences are statistically significant, the main focus in isolating pistils at specific developmental stages is consistency between pistil morphology and its corresponding stage. This relationship is best illustrated in the box plots (Figure 4 and Figures S4–S6), which show that in several genotype–parameter combinations, this consistency is not maintained, as the scatter bars indicate overlap among samples from adjacent stages. A typical example of this issue can be observed for OL or AL across multiple genotypes and stages.

Based on these observations, the parameters OL, SL, and AL did not meet this criterion, as Figures S4–S6 show that the data ranges for consecutive developmental stages often overlapped, leading to the collection of pistils representing mixed stages. In contrast, the parameter PL generally satisfied the criterion, except for stages III and IV, which showed some overlap (Figure 4). As embryo sac development progresses, floral parameters increasingly overlap, making it particularly challenging to distinguish between stages III and IV. Nevertheless, the separation of stages I and II can still be achieved using specific parameters.

To further evaluate each parameter’s ability to discriminate developmental stages, we implemented a simple classification approach based on percentile-derived intervals using a Python (v. 3.11) script. Table 4 and Tables S3–S5 present the classification outputs (precision and recall metrics, interval tables, confusion matrices, and predictions). The PL parameter showed the highest accuracy in assigning pistils to their correct stage, both when using the 50th percentile (precision = 0.791; recall = 0.399) and the 75th percentile (precision = 0.760; recall = 0.578) (Table 4).

**Figure 5:**
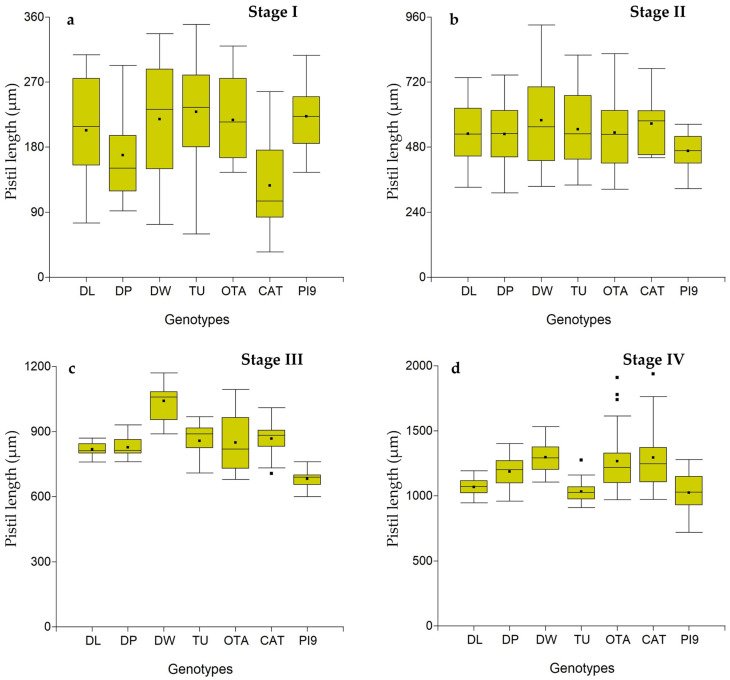
Pistil length for all genotypes, discriminated by female developmental stage. (**a**) Stages: I: Megaspore mother cell, (**b**) Stage II: Postmeiosis or EMMC, (**c**) Stage III: Immature embryo sac, (**d**) Stage IV: Mature embryo sac. Genotypes: DL: Don Luis, DP: Don Pablo, DW: Don Walter, TU: Tanganyika, OTA: OTA-S, CAT: Catalina, and PI9: PI299920.

Stage-specific parameter ranges could be defined within individual genotypes; however, these intervals were not directly transferable across all genotypes (Figure 5 and Figures S7–S9).

### 2.8. Reproductive Calendar

**Figure 6:**
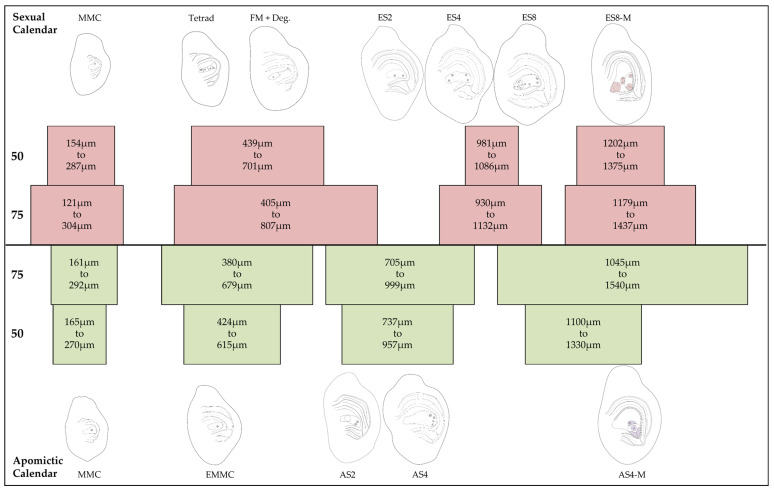
Reproductive calendar of *E. curvula*. Schematic representation of pistil length ranges associated with female developmental stages in OTA-S (sexual) and Don Walter (apomictic). Boxes represent 50th and 75th percentile intervals for each stage. Representative embryo sac structures are illustrated above and below each range for the sexual (MMC to ES8-M) and apomictic (MMC to AS4-M) developmental sequences, respectively. The diagrams highlight the differences in timing and progression between reproductive modes.

Since size ranges for pistil length can be determined at different developmental stages within a genotype, it is possible to create a reproductive calendar for selected representative genotypes, such as the tetraploid sexual OTA-S and the tetraploid facultative apomictic Don Walter (Figure 6). Both genotypes are critical to our research, as extensive data are available, including high-quality genome assemblies, mapping population, genetic linkage maps, transcriptomes from various tissues, and epigenetic studies.

As previously mentioned, PL was the parameter that most effectively discriminated among female developmental stages. However, some overlap between stages may occur for this parameter. To minimize this issue, PL ranges were delimited using percentile-based intervals (50th and 75th percentiles). The use of these narrower and broader ranges minimizes overlap between stages while ensuring sufficient sampling for downstream analyses. This refinement provides greater confidence in the accurate classification of pistils and allows flexible sampling depending on experimental requirements.

## 3. Discussion

This study provides an integrative cytological and morphometric characterization of reproductive development in *Eragrostis curvula* genotypes differing in ploidy level and reproductive mode. By combining ovule clearing, detailed cytoembryological observations, and floral morphometrics, we established a set of reproducible developmental stages that span ovule development from megaspore mother cell differentiation to mature embryo sac formation. The results show that external floral parameters can serve as practical predictors of internal developmental stages, allowing more accurate sampling of reproductive tissues. Similar morphometric approaches have been successfully applied in other grasses and model species, where correlations between floral organ size and gametophytic development have enabled the construction of reproductive calendars and stage-specific sampling strategies [24,25,26,28]. In this context, the framework presented here extends these approaches to *E. curvula*, providing a standardized developmental reference for comparative reproductive studies and for the precise isolation of tissues required in transcriptomic and functional analyses of apomixis.

Among the parameters evaluated, pistil length (PL) was the most accurate overall predictor of female developmental stages, particularly during early development (Stages I and II). In contrast, ovary length (OL) was more informative at later stages (Stages III and IV). This pattern likely reflects spatial constraints within the anthecium: during early development, overall pistil elongation correlates with megasporocyte progression, whereas at later stages, continued ovary enlargement is partially offset by curvature or folding of the styles, limiting further increases in total pistil length. In contrast, ovary growth continues in proportion to gametophyte maturation, which may explain its improved predictive value at advanced stages. Comparable approaches linking external floral morphology with internal reproductive development have been reported in grasses and related species [24,25,26,27,28].

Although the correlation between floral and cytological traits was not consistently strong across genotypes, these features offer a practical way to approximate staging. Percentile-based intervals (50th and 75th percentiles) were used to delineate stages, reducing overlap and enhancing the precision and reproducibility of sample selection for transcriptomic and developmental studies in *E. curvula*.

Our combined cytological and morphometric analyses have enabled us to develop a female reproductive calendar that predicts various gametophytic stages based on floral parameters. This timeline can be monitored using specific metrics, such as PL. Although the correlation between floral parameters and female developmental stages varied among genotypes, pistil length consistently provided the most reliable approximation of developmental stage.

Regarding the synchronization between male and female reproductive development, our study found no differences related to ploidy level or reproductive mode. However, other studied models have shown such differences. For example, Soliman et al. [26] reported that in diploids of *Paspalum rufum*, male and female reproductive development are equally synchronized, whereas in tetraploids, megasporogenesis and early megagametogenesis are delayed compared to microsporogenesis and early microgametogenesis. This delay was also observed when ovary growth served as a reference parameter. In diploids, aposporic initial (AI) cells appeared simultaneously with the female meiocyte tetrad (or triad), whereas in tetraploids, AI cells appeared earlier, at the MMC stage. The extended duration of megasporogenesis in tetraploids may promote AI emergence and aid in the success of apomixis [26]. These differences may reflect the fact that apomixis in *E. curvula* is diplosporic, while in *P. rufum* it is aposporic. In the latter case, apomictic and sexual embryo sacs coexist and compete with each other, whereas this does not occur in diplospory.

Regarding reproductive mode phenotyping, Voigt and Bashaw [3] recommend using the tetrad to identify sexual processes, or the older stages can be useful, for example, when sexual and apomictic embryo sacs are morphologically distinguishable. Our experience shows that the later stages of embryo sac development are the most informative, since observing meiosis is very rare. This is important because it is advisable to collect samples after the appearance of the anthers in the upper anthecia of the spikelet for evaluation.

Regarding the calendar, some authors suggest using other parameters, such as microsporogenesis and microgametogenesis, to estimate the female developmental stage [30]. However, our observations indicate that external pistil parameters are more reliable than those of male development, which can be desynchronized.

A key application of the calendar lies in expression analyses of diplosporous development. Identifying the molecular switch underlying the transition from meiosis to mitosis (apomeiosis) remains a central question in apomixis research [34,35]. The exact timing of this cell-fate transition remains unclear; however, we hypothesize that it occurs during the archesporial stage. This hypothesis highlights the importance of accurately identifying pistils at this developmental stage for transcriptomic analyses. Based on our results, we established floral parameters to identify pistils at this developmental stage (Stage I). This is particularly important for expression studies, since RNA must be extracted from extremely small and fragile tissues. Identifying the mechanisms or genes underlying apomixis will bring us closer to generating apomictic cultures through genetic engineering.

Despite the recurrence of apomixis across plant lineages, no major crop currently exhibits this reproductive strategy [36]. Incorporating apomixis into important agricultural species remains a key goal in plant breeding, as it could maintain hybrid vigor and decrease reliance on clonal propagation [37,38]. Our model system, *E. curvula*, offers several benefits for studying apomixis, including diplospory, a stable embryo–endosperm ratio in both sexual and apomictic seeds, and the absence of polyembryony. These characteristics make it easier to analyze the genetics and development of apomixis and may help in transferring it to crops sensitive to endosperm dosage imbalances, such as maize.

Finally, these findings demonstrate that floral morphometrics can provide a practical framework for reproductive phenotyping in *E. curvula*. Despite variation among genotypes, PL represents a robust indicator of female developmental stage and can guide the collection of stage-specific tissues for both cytological and molecular analyses.

## 4. Materials and Methods

### 4.1. Plant Material

Seeds of *E. curvula* (Schrad.) Nees were obtained from the Instituto Nacional de Tecnología Agropecuaria (INTA, Argentina), the United States Department of Agriculture (USDA, United States), and the CERZOS germplasm collection (CONICET, CCT Bahía Blanca). The study included adult plants from the following genotypes: PI299920 (PI9, accession PI299920, USDA, diploid sexual), Don Walter (DW, INTA, tetraploid facultative apomictic), Tanganyika (TU, accession PI234217, USDA, tetraploid full apomictic), Catalina (CAT, accession PI574519, USDA, tetraploid facultative apomictic), OTA-S (OTA, accession PI574506, USDA, tetraploid sexual), Don Luis (DL, CERZOS, hexaploid facultative apomictic), and Don Pablo (DP, INTA, heptaploid facultative apomictic).

Voucher specimens for each genotype are preserved in the corresponding institutional collections: INTA Germplasm Bank (Argentina), USDA Germplasm Resources Information Network (USDA-GRIN, United States), and CERZOS Germplasm Collection (CONICET–CCT Bahía Blanca, Argentina). No new field collections were conducted for this study. Plants were grown in 10-L pots under greenhouse conditions, following the natural photoperiod of approximately 15 h during the spring flowering period in Bahía Blanca, Buenos Aires, Argentina (38°42′ S, 62°16′ W).

### 4.2. Inflorescence Collection and Preparation

Inflorescences (panicles) from all genotypes were collected at the stage showing initial signs of anthesis in the upper third of the panicle. This stage allows the observation of all developmental phases involved in embryo sac formation, from the archesporial cell to potential fertilization events [23]. For each genotype, at least two panicles per plant were collected and analyzed to account for developmental variability. The total number of pistils evaluated per developmental stage and per genotype is detailed in Table 2; in total, 833 pistils were analyzed across all genotypes and stages. All morphometric measurements were performed on florets collected before anthesis. The analyzed spikelets retained the anthers enclosed within the anthecium, and only pistils in stages before fertilization were evaluated. The panicles were fixed in Farmer’s solution (V_acetic acid_:V_ethanol_ = 1:3) or FAA solution (V_ethanol_:V_distilled water_:V_acetic acid_:V_formaldehyde_ = 10:7:1:2), prepared using analytical-grade reagents. After at least 24 h, the specimens were transferred to 70% ethanol. Spikelets were dissected from the panicle using forceps and a scalpel under a Leica S8APO stereomicroscope equipped with a Leica MC120 HD digital camera (Leica Microsystems, Wetzlar, Germany).

### 4.3. Cytoembryological Studies

To identify male and female developmental stages, the clarification protocol with methyl salicylate described by Young et al. [29] was used with minor modifications. Briefly, spikelets preserved in 70% ethanol were subjected to the following sequential treatments under continuous shaking at room temperature: 95% ethanol for 60 min, 95% ethanol with 0.1% eosin for 30 min, 100% ethanol for 60 min, V_ethanol_:V_methyl salicylate_ = 3:1 for 60 min, V_ethanol_:V_methyl salicylate_ = 1:1 for 60 min, and V_ethanol_:V_methyl salicylate_ = 1:3 for 60 min. Samples were then stored at 4 °C in methyl salicylate.

Clarified spikelets were dissected under a stereomicroscope using fine forceps and a scalpel to isolate pistils with their attached anthers. For mounting, two coverslips were placed parallel to each other on a microscope slide and fixed with a small drop of methyl salicylate to create a narrow channel for the sample. The clarified pistil–anther complex was positioned in the center of this channel, a drop of methyl salicylate was added, and the preparation was covered with a third coverslip. This configuration allowed the tissues to remain immersed and immobile in the clearing solution during observation.

Samples were examined using a Leica DM2500 LED microscope equipped with a DIC system and a Leica MC170 HD digital camera (Leica Microsystems, Germany). Reproductive developmental stages were characterized through cytoembryological observations of cleared material, from the differentiation of the megaspore mother cell (MMC) and microspore mother cell (MiMC) to the mature embryo sac and mature pollen. Additionally, the spatial and temporal distribution of female megasporogenesis and megagametogenesis in the spikelet, as well as male microsporogenesis and microgametogenesis, were determined.

### 4.4. Morphometric Analysis of Floral Bud, Gynoecium, and Anther

To describe reproductive development using quantitative parameters, the following variables were considered, as in other studies [26,28]. Pistil length (PL) was measured along the main axis, from the base of the ovary to the apex of the style. Ovary length (OL) was measured from its base to the uppermost tip. Style length (SL) was recorded from its point of attachment above the ovary to its apex. Anther length (AL) was determined from its base to the most distal tip (Figure S10). Measurements were performed using Leica LAS EZ software (version 3.4.0).

### 4.5. Reproductive Development and Reproductive Calendar Construction

To distinguish between sexual and apomictic pathways and their corresponding developmental stages, we analyzed key cytological features, including the occurrence of meiosis and the number and arrangement of nuclei within the embryo sac [22]. A minimum of 15 observations were performed for each developmental stage per genotype, except for Catalina, where fewer observations were made in stages I and III.

Morphological parameters (PL, OL, SL, and AL) were recorded for each developmental stage. Using these measurements and their relationships with the female and male developmental stages, reproductive calendars were created for sexual and apomictic genotypes.

### 4.6. Statistical and Data Analysis

An analysis of variance (ANOVA) was performed to determine whether significant differences existed among developmental stages within each genotype for each parameter. Box plots were created to explore potential overlaps in developmental stages across genotypes and parameters. All analyses and graphical outputs were generated using INFOSTAT software [39].

A Python script was used to calculate the central 50th and 75th percentile intervals for the OL, SL, PL, and AL measurements within each Genotype-Stage group, and to apply these intervals as simple rule-based classifiers. For each trait and percentile level, the script assigned a Stage only when the observed value fell exclusively within the corresponding interval; cases that fell outside or overlapped multiple intervals were treated as misclassified. The procedure generated all interval estimates, classification outputs, confusion matrices, and precision and recall metrics. Table S6 contains the data used for the analysis, and File S1 contains the script.

## 5. Conclusions

We present here an integrative cytoembryological and morphometric framework for staging female reproductive development in *E. curvula*. Pistil length emerged as the most reliable external parameter for discriminating female developmental stages. The genotype-specific reproductive calendars and percentile-based sampling windows provided here offer practical guidelines to improve the accuracy and reproducibility of tissue collection in developmental and comparative studies. This standardized staging system will facilitate future anatomical, physiological, and developmental research in *E. curvula* and related grasses.

## Figures and Tables

**Table 1:** Accessions and genotypes of *E. curvula* were evaluated, with each accession listed by acronym, seed origin, ploidy level, and reproductive mode.

Genotype (Accession)	Acronym	Origin	Ploidy	Reproductive Mode
PI299920	PI9	USDA	Diploid	Sexual
Don Walter	DW	INTA	Tetraploid	Apomictic
Tanganyika PI234217	TU	USDA	Tetraploid	Apomictic
Catalina PI574519	CAT	USDA	Tetraploid	Apomictic
OTA-S PI574506	OTA	USDA	Tetraploid	Sexual
Don Luis	DL	CERZOS	Hexaploid	Apomictic
Don Pablo	DP	INTA	Heptaploid	Apomictic

**Table 2:** Synchronization between female and male reproductive development in different *E. curvula* genotypes.

Genotype	Female Stage	Arch	MiMC	Meiosis	1N NV	1N V	2N	MP	Total Pistils
DL	I	13	9						22
	II		9	2	7	32	1		51
	III					4	10	1	15
	IV						3	18	21
DP	I	13	3						16
	II		9	10	13	25	1		58
	III					9	8	1	18
	IV						2	25	27
DW	I	37	13						50
	II		17	7	6	26	3		59
	III					8	12	4	24
	IV						2	17	19
TU	I	25	18						43
	II		9	5	15	30	1	1	61
	III					10	8	4	22
	IV						4	33	37
OTA	I	14	1						15
	II		12	6	12	11	11		52
	III					2	17		19
	IV						8	25	33
CAT	I	4	1						5
	II			1	5	9	1		16
	III					2	9	2	13
	IV						4	24	28
PI9	I	12	4						16
	II		4		14	10			28
	III					14			14
	IV					7	11	32	50

Genotypes: DL (Don Luis), DP (Don Pablo), DW (Don Walter), TU (Tanganyika), OTA (OTA-S), CAT (Catalina), and PI9 (PI299920). Female stages (I–IV) represent successive phases of megasporogenesis and megagametogenesis. Male development was divided into seven cytological stages: Arch. (Archesporial pollen cells), MiMC (Microspore mother cell), meiosis, 1N NV (1N Non-vacuolated), 1N V (1N Vacuolated), 2N (Bicellular pollen grain), and MP (mature pollen).

**Table 3:** Average values (±standard deviation) of pistils and anthers measurements for each developmental stage and *E. curvula* genotype.

Genotype	Female Stage	n	OL (µm)	SL (µm)	PL (µm)	AL (µm)
DL	I	22	116 ± 23.7 ^a^	87 ± 56.2 ^a^	203 ± 78.2 ^a^	255 ± 112.7 ^a^
	II	51	206 ± 28.6 ^b^	324 ± 87.4 ^b^	529 ± 114.7 ^b^	722 ± 168.9 ^b^
	III	15	280 ± 12.1 ^c^	538 ± 26.5 ^c^	819 ± 33.3 ^c^	1032 ± 38.3 ^c^
	IV	21	322 ± 13.7 ^d^	745 ± 70.7 ^d^	1068 ± 76.6 ^d^	1126 ± 46.8 ^c^
DP	I	16	105 ± 18.8 ^a^	64 ± 42.5 ^a^	169 ± 59.2 ^a^	182 ± 71.7 ^a^
	II	58	210 ± 32.7 ^b^	318 ± 80.5 ^b^	528 ± 111.1 ^b^	679 ± 149.2 ^b^
	III	18	278 ± 13.3 ^c^	549 ± 42.2 ^c^	827 ± 48 ^c^	979 ± 33.8 ^c^
	IV	27	343 ± 31.5 ^d^	846 ± 98.5 ^d^	1187 ± 119.2 ^d^	1176 ± 108.6 ^d^
DW	I	50	110 ± 18.9 ^a^	109 ± 56 ^a^	218 ± 73.8 ^a^	226 ± 74.2 ^a^
	II	59	195 ± 37.2 ^b^	384 ± 124 ^b^	579 ± 159 ^b^	710 ± 216 ^b^
	III	24	289 ± 13.3 ^c^	752 ± 75.7 ^c^	1042 ± 84.2 ^c^	1121 ± 81.5 ^c^
	IV	19	339 ± 35.5 ^d^	958 ± 103.1 ^d^	1297 ± 116.4 ^d^	1230 ± 76.3 ^d^
TU	I	43	114 ± 23.5 ^a^	115 ± 52.1 ^a^	229 ± 71.9 ^a^	267 ± 96 ^a^
	II	61	200 ± 35.3 ^b^	346 ± 107.1 ^b^	546 ± 137.5 ^b^	655 ± 142.2 ^b^
	III	22	264 ± 19.2 ^c^	593 ± 71.7 ^c^	857 ± 82.4 ^c^	866 ± 109.2 ^c^
	IV	37	314 ± 25 ^d^	720 ± 73.3 ^d^	1033 ± 76.3 ^d^	1012 ± 90.3 ^d^
OTA	I	15	138 ± 20.7 ^a^	79 ± 41.1 ^a^	218 ± 60.2 ^a^	258 ± 87.3 ^a^
	II	52	217 ± 29.7 ^b^	316 ± 97.7 ^b^	533 ± 123.8 ^b^	748 ± 191.3 ^b^
	III	19	280 ± 24.7 ^c^	570 ± 107.5 ^c^	849 ± 125.7 ^c^	1065 ± 127.9 ^c^
	IV	33	348 ± 34.3 ^d^	920 ± 204.1 ^d^	1268 ± 228.6 ^d^	1336 ± 117.7 ^d^
CAT	I	6	85 ± 33.8 ^a^	41 ± 48.1 ^a^	127 ± 79.1 ^a^	133 ± 102.4 ^a^
	II	16	193 ± 22.3 ^b^	373 ± 83.8 ^b^	566 ± 103.6 ^b^	792 ± 135.4 ^b^
	III	13	250 ± 12 ^c^	618 ± 78.7 ^c^	869 ± 85.6 ^c^	1004 ± 45 ^c^
	IV	28	311 ± 29.1 ^d^	983 ± 232.8 ^d^	1295 ± 253.6 ^d^	1084 ± 43.8 ^c^
PI9	I	16	111 ± 14.2 ^a^	111 ± 36.7 ^a^	223 ± 50.3 ^a^	239 ± 72 ^a^
	II	28	173 ± 18.7 ^b^	292 ± 55.5 ^b^	466 ± 70.1 ^d^	734 ± 175.9 ^b^
	III	14	228 ± 6.5 ^c^	455 ± 33.5 ^c^	683 ± 37.1 ^c^	1050 ± 26.4 ^c^
	IV	50	291 ± 29.7 ^d^	733 ± 134.5 ^d^	1024 ± 160.4 ^d^	1331 ± 131.4 ^d^

Pistil length (PL), ovary length (OL), style length (SL), and anther length (AL). Female developmental stages: I: Megaspore Mother Cell, II: Postmeiosis or EMMC, III: Immature embryo sac, and IV: Mature embryo sac. Genotypes: PI9: PI299920, OTA: OTA-S, TU: Tanganyika, DW: Don Walter, CAT: Catalina, DL: Don Luis, and DP: Don Pablo. Values with different letters differ significantly according to Tukey’s HSD test (α = 0.05).

**Table 4:** Precision and recall metrics based on 50th- and 75th-percentile ranges for morphometric parameters across *E. curvula* genotypes.

Percentile	Parameter	Label	Precision	Recall
50	OL	I	1.000	0.512
		II	0.994	0.529
		III	0.793	0.584
		IV	0.982	0.502
		**MACRO**	**0.754**	**0.425**
	SL	I	1.000	0.506
		II	1.000	0.505
		III	0.908	0.472
		IV	0.964	0.498
		**MACRO**	**0.774**	**0.396**
	PL	I	1.000	0.500
		II	1.000	0.498
		III	0.954	0.496
		IV	1.000	0.502
		**MACRO**	**0.791**	**0.399**
	AL	I	1.000	0.488
		II	0.982	0.502
		III	0.621	0.472
		IV	0.901	0.507
		**MACRO**	**0.701**	**0.394**
75	OL	I	0.977	0.750
		II	0.979	0.729
		III	0.791	0.728
		IV	0.975	0.726
		**MACRO**	**0.744**	**0.587**
	SL	I	1.000	0.750
		II	0.988	0.738
		III	0.776	0.720
		IV	0.950	0.702
		**MACRO**	**0.743**	**0.582**
	PL	I	1.000	0.726
		II	0.988	0.732
		III	0.824	0.712
		IV	0.987	0.721
		**MACRO**	**0.760**	**0.578**
	AL	I	1.000	0.726
		II	0.975	0.708
		III	0.654	0.544
		IV	0.928	0.656
		**MACRO**	**0.711**	**0.527**

PL: pistil length; OL: ovary length; SL: style length; AL: anther length. Female developmental stages: I, Megaspore Mother Cell; II, Postmeiosis or EMMC; III, Immature embryo sac; IV, Mature embryo sac.

## Data Availability

All datasets supporting the findings of this study are provided within the article and the Supplementary Materials (raw morphometric measurements, ANOVA outputs, classification analyses, and Python scripts). Any additional raw data or image files used during the analyses are available from the corresponding author upon reasonable request.

## Supplementary Materials

The following supporting information can be downloaded at: https://www.mdpi.com/article/10.3390/plants15071050/s1, Figure S1: Schematic representation of panicle and spikelet morphology of *E. curvula*; Figure S2: Anthecia per spikelet; Figure S3: OTA spikelet; Figure S4: Ovary length in all genotypes; Figure S5: Style length in all genotypes; Figure S6: Anther length in all genotypes; Figure S7: Ovary length in all stages; Figure S8: Style length in all stages; Figure S9: Anther length in all stages; Figure S10: Parameters; Table S1: Raw data used in the study; Table S2: Results of ANOVA comparing morphological parameters in each genotype and all genotypes in each parameter. F and *p*-values are reported for each analysis; Table S3: Interval table generated from the 50th and 75th percentiles. Table shows for each stage and genotype the low, high and center values of each parameter; Table S4: Confusion table generated from the 50th and 75th percentiles to assess the classification accuracy of each parameter; Table S5: Predictions generated from the 50th and 75th percentiles to assess the classification accuracy of each parameter; Table S6: Data used in the script to assess the classification accuracy of each parameter; File S1: Classification script.
