# Supplementary material for: A Precise Reproductive Calendar of Sexual and Apomictic Genotypes of Eragrostis curvula
Source: Plants (Basel). 2026 Mar 29;15(7):1050. doi: 10.3390/plants15071050 (PMC13074311; doi:10.3390/plants15071050)
Supplement: Supplementary file 1 [file plants-15-01050-s001.zip › supplementary material/Table S1. Raw data used in the study.pdf]

Table S1. Raw data used in the study.

DL	OL (µm)	SL (µm)	PL (µm)	AL (µm)	♀ Stage	♂ Stage
	188	271	458	628	II	1N non-vacuolated
	181	266	447	592	II	1N non-vacuolated
	183	239	422	603	II	1N non-vacuolated
	173	292	465	620	II	1N non-vacuolated
	198	269	467	582	II	1N non-vacuolated
	186	237	423	582	II	1N non-vacuolated
	190	278	468	618	II	1N non-vacuolated
	219	340	559	736	II	1N vacuolated
	221	432	653	922	II	1N vacuolated
	207	337	545	730	II	1N vacuolated
	250	446	696	926	II	1N vacuolated
	216	388	603	785	II	1N vacuolated
	271	540	811	980	III	1N vacuolated
	237	380	618	858	II	1N vacuolated
	213	322	535	712	II	1N vacuolated
	297	515	813	1008	III	1N vacuolated
	226	378	604	876	II	1N vacuolated
	200	328	528	732	II	1N vacuolated
	235	364	599	900	II	1N vacuolated
	212	315	527	723	II	1N vacuolated
	255	449	704	961	II	1N vacuolated
	227	400	627	842	II	1N vacuolated
	192	304	496	663	II	1N vacuolated
	230	401	631	906	II	1N vacuolated
	237	393	630	771	II	1N vacuolated
	225	458	683	966	II	1N vacuolated
	213	305	518	859	II	1N vacuolated
	180	266	446	686	II	1N vacuolated
	265	545	810	980	III	1N vacuolated
	221	381	602	887	II	1N vacuolated
	210	352	562	740	II	1N vacuolated
	240	439	679	927	II	1N vacuolated
	203	313	516	797	II	1N vacuolated
	238	413	651	937	II	1N vacuolated
	202	346	548	807	II	1N vacuolated
	262	498	760	1005	III	1N vacuolated
	245	433	678	831	II	1N vacuolated
	233	391	624	909	II	1N vacuolated
	218	398	616	793	II	1N vacuolated
	254	483	737	956	II	1N vacuolated
	221	383	604	808	II	1N vacuolated
	192	312	503	696	II	1N vacuolated

191	302	493	760	II	1N vacuolated
261	462	723	920	II	2N
281	519	801	1011	III	2N
312	718	1030	1079	IV	2N
298	573	871	1042	III	2N
279	506	786	1031	III	2N
293	573	866	1064	III	2N
294	546	841	1112	III	2N
268	508	776	1039	III	2N
319	658	976	1076	IV	2N
304	642	946	1077	IV	2N
271	537	808	1052	III	2N
280	564	844	977	III	2N
290	523	812	1060	III	2N
286	582	867	1066	III	2N
121	65	186	216	I	Arch
80	0	80	110	I	Arch
108	86	194	226	I	Arch
65	10	75	70	I	Arch
104	0	104	114	I	Arch
122	87	209	315	I	Arch
125	104	229	263	I	Arch
75	15	90	112	I	Arch
117	92	208	269	I	Arch
90	10	100	110	I	Arch
105	50	155	190	I	Arch
105	56	161	199	I	Arch
109	61	170	163	I	Arch
170	218	388	553	II	MiMC
120	149	269	353	I	MiMC
162	204	366	494	II	MiMC
147	185	332	398	II	MiMC
174	195	368	496	II	MiMC
118	107	224	284	I	MiMC
185	191	376	530	II	MiMC
158	198	356	421	II	MiMC
142	133	275	324	I	MiMC
170	203	373	463	II	MiMC
124	123	247	302	I	MiMC
147	153	300	410	I	MiMC
159	177	336	400	II	MiMC
141	158	299	469	I	MiMC
165	174	339	414	II	MiMC
153	155	308	388	I	MiMC
136	158	295	380	I	MiMC

	143	147	291	340	I	MiMC
	179	230	409	553	II	Dyad
	197	273	470	540	II	Dyad
	339	855	1193	1231	IV	Mature pollen
	341	658	999	1167	IV	Mature pollen
	305	656	961	1086	IV	Mature pollen
	323	815	1137	1156	IV	Mature pollen
	347	836	1184	1187	IV	Mature pollen
	321	758	1078	1080	IV	Mature pollen
	271	545	816	1055	III	Mature pollen
	326	781	1107	1124	IV	Mature pollen
	326	702	1028	1080	IV	Mature pollen
	302	721	1023	1118	IV	Mature pollen
	321	753	1074	1112	IV	Mature pollen
	320	797	1117	1162	IV	Mature pollen
	298	814	1112	1114	IV	Mature pollen
	336	805	1141	1199	IV	Mature pollen
	328	627	955	1092	IV	Mature pollen
	341	845	1186	1131	IV	Mature pollen
	325	747	1072	1082	IV	Mature pollen
	311	750	1061	1185	IV	Mature pollen
	326	710	1036	1112	IV	Mature pollen
**$\bar{x}$**	220	387	607	748		
**n**	109	109	109	109		

DP	OL (μm)	SL (μm)	PL (μm)	AL (μm)	♀ Stage	♂ Stage
	178	285	462	692	II	1N non-vacuolated
	215	327	542	723	II	1N non-vacuolated
	213	310	524	665	II	1N non-vacuolated
	226	335	561	701	II	1N non-vacuolated
	236	319	555	663	II	1N non-vacuolated
	218	309	528	690	II	1N non-vacuolated
	211	293	504	651	II	1N non-vacuolated
	224	299	523	737	II	1N non-vacuolated
	202	325	527	661	II	1N non-vacuolated
	223	359	581	754	II	1N non-vacuolated
	212	283	496	665	II	1N non-vacuolated
	203	324	528	620	II	1N non-vacuolated
	223	326	548	700	II	1N non-vacuolated
	222	394	615	889	II	1N vacuolated
	222	405	627	874	II	1N vacuolated
	208	324	532	702	II	1N vacuolated
	266	496	762	991	III	1N vacuolated

251	445	696	891	II	1N vacuolated
251	435	686	813	II	1N vacuolated
236	372	608	785	II	1N vacuolated
259	525	784	941	III	1N vacuolated
217	301	518	752	II	1N vacuolated
174	222	396	492	II	1N vacuolated
199	362	560	719	II	1N vacuolated
261	418	678	850	II	1N vacuolated
272	548	821	960	III	1N vacuolated
237	373	610	728	II	1N vacuolated
270	505	775	961	III	1N vacuolated
257	432	689	861	II	1N vacuolated
289	511	800	943	III	1N vacuolated
276	536	812	1000	III	1N vacuolated
229	410	640	850	II	1N vacuolated
273	539	812	930	III	1N vacuolated
247	397	644	769	II	1N vacuolated
220	369	589	826	II	1N vacuolated
295	605	899	994	III	1N vacuolated
218	370	588	750	II	1N vacuolated
264	547	811	936	III	1N vacuolated
209	403	612	770	II	1N vacuolated
223	355	578	742	II	1N vacuolated
263	408	671	854	II	1N vacuolated
250	445	695	885	II	1N vacuolated
253	386	638	734	II	1N vacuolated
276	470	746	852	II	1N vacuolated
241	339	580	772	II	1N vacuolated
247	399	646	811	II	1N vacuolated
262	385	647	903	II	1N vacuolated
304	657	961	1016	IV	2N
219	410	629	961	II	2N
289	605	893	981	III	2N
273	500	772	948	III	2N
284	647	931	999	III	2N
290	527	816	1019	III	2N
274	530	804	997	III	2N
253	568	821	952	III	2N
280	606	886	1005	III	2N
300	563	864	1018	III	2N
312	657	969	999	IV	2N
94	46	140	166	I	Arch
124	67	191	212	I	Arch
81	36	117	100	I	Arch
92	0	92	95	I	Arch

107	34	142	160	I	Arch
90	29	119	113	I	Arch
109	87	196	248	I	Arch
81	48	128	152	I	Arch
102	96	198	161	I	Arch
84	26	110	137	I	Arch
96	39	136	153	I	Arch
109	51	160	160	I	Arch
105	61	166	158	I	Arch
156	181	337	440	II	MiMC
153	200	353	465	II	MiMC
161	193	354	461	II	MiMC
154	157	311	370	II	MiMC
129	106	235	237	I	MiMC
164	210	374	434	II	MiMC
169	199	369	444	II	MiMC
151	177	328	425	II	MiMC
166	222	388	445	II	MiMC
137	140	277	342	I	MiMC
138	155	293	316	I	MiMC
179	272	451	517	II	MiMC
172	258	431	535	II	Dyad
166	204	370	551	II	Dyad
194	276	470	550	II	Dyad
384	999	1383	1328	IV	Mature pollen
345	906	1252	1281	IV	Mature pollen
342	988	1329	1200	IV	Mature pollen
384	916	1300	1335	IV	Mature pollen
355	834	1189	1138	IV	Mature pollen
315	793	1108	1111	IV	Mature pollen
357	848	1205	1278	IV	Mature pollen
348	869	1217	1151	IV	Mature pollen
332	919	1251	1132	IV	Mature pollen
289	760	1049	1026	IV	Mature pollen
324	762	1086	1100	IV	Mature pollen
333	938	1271	1104	IV	Mature pollen
349	854	1202	1092	IV	Mature pollen
285	735	1020	1046	IV	Mature pollen
334	808	1142	1100	IV	Mature pollen
325	773	1098	1085	IV	Mature pollen
296	532	828	1048	III	Mature pollen
394	830	1224	1270	IV	Mature pollen
379	894	1273	1292	IV	Mature pollen
338	775	1150	1248	IV	Mature pollen
320	881	1201	1150	IV	Mature pollen

	360	959	1200	1234	IV	Mature pollen
	315	689	1004	1116	IV	Mature pollen
	369	940	1309	1210	IV	Mature pollen
	368	878	1246	1340	IV	Mature pollen
	411	993	1404	1367	IV	Mature pollen
	196	258	454	632	II	Tetrad
	193	287	479	591	II	Tetrad
	190	253	443	516	II	Tetrad
	175	268	443	558	II	Tetrad
	194	205	399	570	II	Tetrad
	180	253	432	563	II	Tetrad
	189	268	456	563	II	Tetrad
**$\bar{x}$**	236	439	675	770		
**n**	119	119	119	119		

DW	OL (µm)	SL (µm)	PL (µm)	AL (µm)	♀ Stage	♂ Stage
	175	361	536	628	II	1N non-vacuolated
	206	415	621	677	II	1N non-vacuolated
	189	366	555	620	II	1N non-vacuolated
	205	434	639	753	II	1N non-vacuolated
	158	249	407	649	II	1N non-vacuolated
	195	350	545	670	II	1N non-vacuolated
	251	563	813	1050	II	1N vacuolated
	243	595	837	962	II	1N vacuolated
	190	354	544	706	II	1N vacuolated
	233	588	821	992	II	1N vacuolated
	206	416	622	743	II	1N vacuolated
	275	680	955	1077	III	1N vacuolated
	195	508	703	892	II	1N vacuolated
	280	663	943	1012	III	1N vacuolated
	228	477	706	891	II	1N vacuolated
	263	548	811	978	II	1N vacuolated
	185	461	646	781	II	1N vacuolated
	191	393	584	832	II	1N vacuolated
	283	651	934	1082	III	1N vacuolated
	222	486	708	894	II	1N vacuolated
	256	584	840	1007	II	1N vacuolated
	190	356	546	787	II	1N vacuolated
	226	590	816	997	II	1N vacuolated
	194	401	595	764	II	1N vacuolated
	279	613	892	1012	III	1N vacuolated
	205	428	633	787	II	1N vacuolated
	247	514	761	982	II	1N vacuolated

202	402	605	742	II	1N vacuolated
232	562	794	1015	II	1N vacuolated
206	441	647	1030	II	1N vacuolated
193	422	615	822	II	1N vacuolated
295	695	990	1100	III	1N vacuolated
305	724	1029	1091	III	1N vacuolated
249	454	703	893	II	1N vacuolated
274	629	903	1068	III	1N vacuolated
231	507	738	845	II	1N vacuolated
226	446	672	754	II	1N vacuolated
237	403	640	877	II	1N vacuolated
253	637	890	941	III	1N vacuolated
217	483	700	765	II	1N vacuolated
281	650	931	1117	II	2N
308	776	1084	1042	III	2N
210	455	665	965	II	2N
290	816	1106	1091	IV	2N
273	637	910	1134	II	2N
309	774	1083	1214	III	2N
292	802	1094	1157	III	2N
283	775	1058	1137	III	2N
294	767	1061	1193	III	2N
291	767	1058	1136	III	2N
291	779	1070	1112	III	2N
277	751	1028	1088	III	2N
298	780	1077	1121	III	2N
286	764	1051	1136	III	2N
290	791	1081	1174	III	2N
338	945	1283	1146	IV	2N
298	872	1170	1067	III	2N
130	125	255	290	I	Arch
105	45	150	170	I	Arch
107	96	203	230	I	Arch
92	55	147	154	I	Arch
135	175	310	315	I	Arch
95	80	175	175	I	Arch
130	155	285	305	I	Arch
130	115	245	285	I	Arch
95	80	175	165	I	Arch
120	120	240	240	I	Arch
120	135	255	265	I	Arch
125	155	280	285	I	Arch
120	115	235	225	I	Arch
130	170	300	320	I	Arch
73	36	109	117	I	Arch

130	165	295	315	I	Arch
115	95	210	240	I	Arch
105	125	230	240	I	Arch
120	80	200	245	I	Arch
100	50	150	190	I	Arch
105	89	194	179	I	Arch
116	140	257	254	I	Arch
84	58	143	138	I	Arch
112	127	240	221	I	Arch
79	39	118	108	I	Arch
107	104	211	167	I	Arch
89	5	89	105	I	Arch
77	19	96	114	I	Arch
99	99	198	205	I	Arch
129	171	300	284	I	Arch
104	75	178	171	I	Arch
68	5	68	80	I	Arch
92	40	131	148	I	Arch
99	51	150	149	I	Arch
83	5	83	109	I	Arch
89	31	120	126	I	Arch
92	73	165	176	I	Arch
145	211	357	351	II	MiMC
144	265	409	468	II	MiMC
147	234	381	404	II	MiMC
133	179	312	250	I	MiMC
125	141	266	334	I	MiMC
141	227	368	369	II	MiMC
162	292	454	535	II	MiMC
130	177	307	316	I	MiMC
148	236	384	492	II	MiMC
128	177	305	273	I	MiMC
161	288	448	516	II	MiMC
125	165	290	281	I	MiMC
156	270	426	471	II	MiMC
105	134	239	263	I	MiMC
148	273	421	496	II	MiMC
99	153	252	258	I	MiMC
132	205	337	303	I	MiMC
165	240	405	485	II	MiMC
143	192	335	362	II	MiMC
128	167	295	321	I	MiMC
126	162	288	297	I	MiMC
185	245	430	525	II	MiMC
175	300	475	530	II	MiMC

	145	260	405	430	II	MiMC
	144	206	350	376	II	MiMC
	145	240	385	450	II	MiMC
	95	108	203	226	I	MiMC
	140	190	330	365	I	MiMC
	126	179	305	318	I	MiMC
	155	255	410	485	II	MiMC
	178	319	497	577	II	Dyad
	171	279	451	552	II	Dyad
	171	307	478	570	II	Dyad
	168	285	453	558	II	Dyad
	346	1187	1533	1320	IV	Mature pollen
	286	874	1160	1296	III	Mature pollen
	308	948	1256	1313	IV	Mature pollen
	329	874	1202	1198	IV	Mature pollen
	315	900	1215	1363	IV	Mature pollen
	330	845	1175	1280	IV	Mature pollen
	378	954	1332	1285	IV	Mature pollen
	305	853	1158	1247	III	Mature pollen
	331	1045	1377	1306	IV	Mature pollen
	311	892	1203	1229	IV	Mature pollen
	308	819	1128	1205	III	Mature pollen
	330	962	1292	1230	IV	Mature pollen
	284	823	1106	1200	III	Mature pollen
	301	858	1159	1240	IV	Mature pollen
	340	1064	1404	1290	IV	Mature pollen
	334	1039	1373	1198	IV	Mature pollen
	316	875	1191	1208	IV	Mature pollen
	351	960	1311	1239	IV	Mature pollen
	449	850	1299	1211	IV	Mature pollen
	375	1101	1476	1143	IV	Mature pollen
	363	1085	1448	1088	IV	Mature pollen
	174	342	516	611	II	Tetrad
	212	308	520	628	II	Tetrad
	192	314	506	650	II	Tetrad
$\bar{x}$	200	424	623	681		
**n**	152	152	152	152		

**OTA**	**OL (μm)**	**SL (μm)**	**PL (μm)**	**AL (μm)**	**♀ Stage**	**♂ Stage**
	225	325	550	750	II	1N non-vacuolated
	214	271	485	750	II	1N non-vacuolated
	200	225	425	740	II	1N non-vacuolated
	230	380	610	810	II	1N non-vacuolated

215	310	525	730	II	1N non-vacuolated
211	259	470	680	II	1N non-vacuolated
225	325	550	685	II	1N non-vacuolated
210	315	525	640	II	1N non-vacuolated
220	355	575	715	II	1N non-vacuolated
225	235	460	630	II	1N non-vacuolated
200	330	530	660	II	1N non-vacuolated
220	300	520	670	II	1N non-vacuolated
240	400	640	870	II	1N vacuolated
205	265	470	780	II	1N vacuolated
230	370	600	880	II	1N vacuolated
255	405	660	955	II	1N vacuolated
230	390	620	890	II	1N vacuolated
250	350	600	705	II	1N vacuolated
240	390	630	910	II	1N vacuolated
265	435	700	795	III	1N vacuolated
240	345	585	810	II	1N vacuolated
285	525	810	1145	II	1N vacuolated
210	365	575	870	II	1N vacuolated
300	595	895	1245	III	1N vacuolated
230	385	615	1035	II	1N vacuolated
270	550	820	1020	III	2N
245	475	720	1020	III	2N
265	455	720	1010	II	2N
250	455	705	1000	II	2N
285	445	730	895	III	2N
220	410	630	950	II	2N
260	430	690	995	II	2N
240	440	680	980	III	2N
230	370	600	890	II	2N
240	355	595	885	II	2N
240	325	565	795	II	2N
245	500	745	1115	III	2N
200	415	615	955	II	2N
265	685	950	1100	III	2N
265	530	795	950	III	2N
265	535	800	975	III	2N
265	430	695	940	III	2N
250	480	730	935	II	2N
320	645	965	1280	III	2N
310	690	1000	1335	IV	2N
295	580	875	1265	III	2N
275	430	705	1130	II	2N
260	565	825	1090	II	2N
300	705	1005	1135	III	2N

325	695	1020	1100	III	2N
360	730	1090	1240	IV	2N
335	705	1040	1150	IV	2N
275	515	790	1050	III	2N
295	780	1075	1200	IV	2N
300	580	880	1065	III	2N
325	645	970	1160	IV	2N
315	690	1005	1190	IV	2N
305	790	1095	1125	III	2N
285	695	980	1175	III	2N
315	745	1060	1390	IV	2N
365	825	1190	1310	IV	2N
135	80	215	270	I	Arch
125	45	170	175	I	Arch
175	145	320	390	I	Arch
155	80	235	240	I	Arch
115	50	165	190	I	Arch
105	40	145	140	I	Arch
120	30	150	180	I	Arch
140	90	230	225	I	Arch
150	125	275	335	I	Arch
145	120	265	320	I	Arch
135	40	175	245	I	Arch
160	130	290	385	I	Arch
120	45	165	180	I	Arch
125	40	165	195	I	Arch
160	165	325	475	II	MiMC
180	215	395	510	II	MiMC
160	195	355	410	II	MiMC
205	250	455	635	II	MiMC
170	190	360	500	II	MiMC
170	210	380	540	II	MiMC
200	175	375	500	II	MiMC
170	190	360	415	II	MiMC
190	180	370	540	II	MiMC
176	214	390	500	II	MiMC
170	130	300	400	I	MiMC
165	240	405	520	II	MiMC
210	195	405	505	II	MiMC
385	1155	1540	1420	IV	Mature pollen
370	1010	1380	1370	IV	Mature pollen
315	1025	1340	1185	IV	Mature pollen
325	1005	1330	1265	IV	Mature pollen
340	1025	1365	1240	IV	Mature pollen
325	840	1165	1300	IV	Mature pollen

	345	815	1160	1350	IV	Mature pollen
	325	835	1160	1330	IV	Mature pollen
	415	1365	1780	1660	IV	Mature pollen
	350	970	1320	1325	IV	Mature pollen
	355	925	1280	1330	IV	Mature pollen
	330	940	1270	1370	IV	Mature pollen
	415	1200	1615	1500	IV	Mature pollen
	315	960	1275	1345	IV	Mature pollen
	320	815	1135	1250	IV	Mature pollen
	390	1350	1740	1560	IV	Mature pollen
	350	870	1220	1295	IV	Mature pollen
	430	1480	1910	1580	IV	Mature pollen
	380	820	1200	1240	IV	Mature pollen
	395	790	1185	1400	IV	Mature pollen
	305	975	1280	1310	IV	Mature pollen
	345	985	1330	1380	IV	Mature pollen
	360	920	1280	1405	IV	Mature pollen
	335	765	1100	1300	IV	Mature pollen
	330	715	1045	1400	IV	Mature pollen
	190	190	380	650	II	Tetrad
	230	280	510	690	II	Tetrad
	205	260	465	680	II	Tetrad
	215	300	515	680	II	Tetrad
	200	220	420	640	II	Tetrad
	205	245	450	580	II	Tetrad
$\bar{x}$	253	494	748	900		
**n**	119	119	119	119		

**TU**	**OL (µm)**	**SL (µm)**	**PL (µm)**	**AL (µm)**	**♀ Stage**	**♂ Stage**
	170	285	455	595	II	1N non-vacuolated
	220	325	545	615	II	1N non-vacuolated
	190	335	525	670	II	1N non-vacuolated
	175	275	450	595	II	1N non-vacuolated
	155	250	405	585	II	1N non-vacuolated
	160	265	425	530	II	1N non-vacuolated
	185	250	435	590	II	1N non-vacuolated
	175	320	495	580	II	1N non-vacuolated
	200	370	570	605	II	1N non-vacuolated
	180	325	505	575	II	1N non-vacuolated
	165	290	455	575	II	1N non-vacuolated
	185	280	465	600	II	1N non-vacuolated
	170	230	400	500	II	1N non-vacuolated
	197	308	505	644	II	1N non-vacuolated

175	354	529	585	II	1N non-vacuolated
240	460	700	760	II	1N vacuolated
200	350	550	620	II	1N vacuolated
190	350	540	720	II	1N vacuolated
200	380	580	610	II	1N vacuolated
220	400	620	720	II	1N vacuolated
190	510	700	735	II	1N vacuolated
255	455	710	726	III	1N vacuolated
250	575	825	690	III	1N vacuolated
240	200	440	600	II	1N vacuolated
200	262	462	620	II	1N vacuolated
250	575	825	765	III	1N vacuolated
245	470	715	875	II	1N vacuolated
260	480	740	875	II	1N vacuolated
230	325	555	730	II	1N vacuolated
205	340	545	695	II	1N vacuolated
230	345	575	720	II	1N vacuolated
285	535	820	890	II	1N vacuolated
255	470	725	845	II	1N vacuolated
205	275	480	700	II	1N vacuolated
275	525	800	790	II	1N vacuolated
250	515	765	765	III	1N vacuolated
240	510	750	725	II	1N vacuolated
215	395	610	650	II	1N vacuolated
265	625	890	815	III	1N vacuolated
230	670	900	850	III	1N vacuolated
235	535	770	770	II	1N vacuolated
250	575	825	790	III	1N vacuolated
200	425	625	675	II	1N vacuolated
225	340	565	775	II	1N vacuolated
229	548	777	869	II	1N vacuolated
184	344	529	687	II	1N vacuolated
254	537	791	932	II	1N vacuolated
261	508	769	884	II	1N vacuolated
264	507	771	920	II	1N vacuolated
168	257	425	701	II	1N vacuolated
227	486	713	783	III	1N vacuolated
225	444	670	756	II	1N vacuolated
278	571	849	791	III	1N vacuolated
208	484	692	705	II	1N vacuolated
243	484	728	785	III	1N vacuolated
250	485	735	780	III	2N
280	550	830	920	III	2N
185	370	555	875	II	2N
285	665	950	870	III	2N

270	680	950	910	IV	2N
282	635	917	896	III	2N
250	685	935	865	III	2N
270	635	905	945	III	2N
355	695	1050	890	IV	2N
285	645	930	930	III	2N
270	680	950	885	IV	2N
295	621	916	958	III	2N
294	798	1092	979	IV	2N
75	45	120	190	I	Arch
95	75	170	210	I	Arch
100	135	235	245	I	Arch
120	80	200	245	I	Arch
110	115	225	265	I	Arch
100	90	190	230	I	Arch
115	85	200	230	I	Arch
110	80	190	190	I	Arch
95	85	180	170	I	Arch
100	65	165	170	I	Arch
100	50	150	165	I	Arch
100	70	170	170	I	Arch
120	140	260	315	I	Arch
115	210	325	280	I	Arch
120	145	265	325	I	Arch
150	160	310	380	I	Arch
120	75	195	245	I	Arch
120	110	230	260	I	Arch
92	29	121	122	I	Arch
116	107	223	219	I	Arch
75	22	97	98	I	Arch
78	87	165	202	I	Arch
40	20	60	83	I	Arch
98	82	180	163	I	Arch
79	18	97	93	I	Arch
130	210	340	360	I	MiMC
130	210	340	480	I	MiMC
135	145	280	340	I	MiMC
140	125	265	335	I	MiMC
175	230	405	500	II	MiMC
145	145	290	375	I	MiMC
160	230	390	490	II	MiMC
150	195	345	450	I	MiMC
160	180	340	425	II	MiMC
151	153	304	390	I	MiMC
150	135	285	380	I	MiMC

165	195	360	465	II	MiMC
155	245	400	510	II	MiMC
185	245	430	500	II	MiMC
150	225	365	440	II	MiMC
145	205	350	425	I	MiMC
115	135	250	315	I	MiMC
125	140	265	310	I	MiMC
120	140	260	300	I	MiMC
125	125	250	305	I	MiMC
125	155	280	310	I	MiMC
128	143	271	326	I	MiMC
150	190	340	427	II	MiMC
120	135	255	303	I	MiMC
120	161	281	310	I	MiMC
142	210	352	409	II	MiMC
110	101	211	221	I	MiMC
164	246	410	507	II	Dyad
163	241	404	496	II	Dyad
167	276	443	505	II	Dyad
314	826	1140	940	IV	Mature pollen
360	550	910	960	IV	Mature pollen
330	620	950	840	IV	Mature pollen
340	690	1030	1165	IV	Mature pollen
280	690	970	1045	IV	Mature pollen
330	660	990	1050	IV	Mature pollen
225	460	685	955	II	Mature pollen
335	630	965	1000	IV	Mature pollen
270	705	975	1035	IV	Mature pollen
325	655	980	1060	IV	Mature pollen
315	675	990	1160	IV	Mature pollen
310	685	995	1140	IV	Mature pollen
345	640	985	1015	IV	Mature pollen
275	625	900	1040	III	Mature pollen
325	825	1150	975	IV	Mature pollen
290	710	1000	965	IV	Mature pollen
275	665	940	875	IV	Mature pollen
350	925	1275	975	IV	Mature pollen
320	740	1060	935	IV	Mature pollen
310	740	1050	975	IV	Mature pollen
300	700	1000	960	IV	Mature pollen
310	665	975	950	IV	Mature pollen
290	850	1140	985	IV	Mature pollen
322	813	1135	1148	IV	Mature pollen
285	684	969	1106	III	Mature pollen
346	725	1071	1190	IV	Mature pollen

	305	665	969	1082	IV	Mature pollen
	333	743	1076	1179	IV	Mature pollen
	285	752	1037	1066	IV	Mature pollen
	320	706	1026	1099	IV	Mature pollen
	270	679	949	1026	III	Mature pollen
	298	738	1036	946	IV	Mature pollen
	306	722	1028	985	IV	Mature pollen
	322	763	1085	1038	IV	Mature pollen
	287	755	1041	937	IV	Mature pollen
	280	611	891	958	III	Mature pollen
	333	829	1162	1052	IV	Mature pollen
	332	716	1048	1050	IV	Mature pollen
	190	320	510	530	II	Tetrad
	185	295	480	550	II	Tetrad
**$\bar{x}$**	212	403	615	662		
**n**	163	163	163	163		

**CAT**	**OL**	**SL**	**PL**	**AL**	**♀ Stage**	**♂ Stage**
	186	283	469	759	II	1N non-vacuolated
	162	281	443	717	II	1N non-vacuolated
	169	281	451	746	II	1N non-vacuolated
	156	289	445	684	II	1N non-vacuolated
	177	314	492	744	II	1N non-vacuolated
	263	590	853	991	III	1N vacuolated
	189	385	573	876	II	1N vacuolated
	205	376	582	885	II	1N vacuolated
	201	406	607	864	II	1N vacuolated
	222	441	663	878	II	1N vacuolated
	217	419	636	879	II	1N vacuolated
	206	401	607	886	II	1N vacuolated
	214	400	614	408	II	1N vacuolated
	218	506	724	826	II	1N vacuolated
	174	371	546	860	II	1N vacuolated
	239	495	733	986	III	1N vacuolated
	266	622	888	977	III	2N
	237	647	884	987	III	2N
	246	688	934	974	III	2N
	249	640	890	1008	III	2N
	229	478	707	1018	III	2N
	255	576	831	980	III	2N
	240	590	831	1001	III	2N
	222	548	770	990	II	2N
	251	656	907	985	III	2N

	275	698	974	1016	IV	2N
	255	586	841	967	III	2N
	282	740	1022	1005	IV	2N
	274	731	1005	1045	IV	2N
	297	944	1241	1054	IV	2N
	82	41	123	137	I	Arq
	80	0	83	82	I	Arq
	35	0	35	38	I	Arq
	70	18	88	104	I	Arq
	131	126	257	305	I	MiMC
	346	1318	1664	1169	IV	Pollen
	281	774	1055	1070	IV	Pollen
	371	1394	1765	1109	IV	Pollen
	269	1013	1282	1055	IV	Pollen
	309	944	1254	1076	IV	Pollen
	321	985	1306	1138	IV	Pollen
	354	1333	1687	1159	IV	Pollen
	300	943	1243	1057	IV	Pollen
	384	1151	1535	1142	IV	Pollen
	328	968	1296	1110	IV	Pollen
	313	1061	1373	1087	IV	Pollen
	297	989	1285	1067	IV	Pollen
	346	1100	1445	1110	IV	Pollen
	298	812	1110	1066	IV	Pollen
	322	1615	1937	1109	IV	Pollen
	295	818	1113	1054	IV	Pollen
	312	1319	1631	1112	IV	Pollen
	286	752	1038	1119	IV	Pollen
	269	712	982	1046	III	Pollen
	321	1003	1324	1117	IV	Pollen
	256	754	1010	1137	III	Pollen
	287	745	1032	1007	IV	Pollen
	308	806	1115	1036	IV	Pollen
	322	888	1210	1057	IV	Pollen
	326	872	1198	1101	IV	Pollen
	292	817	1108	1116	IV	Pollen
	177	264	441	668	II	Tetrad
	114	63	176		I	
**$\bar{x}$**	247	663	911	915		
**n**	63	63	63	62		

**PI9**	**OL**	**SL**	**PL**	**AL**	**♀ Stage**	**♂ Stage**
	165	250	415	605	II	1N non-vacuolated

160	260	420	655	II	1N non-vacuolated
175	295	470	695	II	1N non-vacuolated
160	305	465	785	II	1N non-vacuolated
180	345	525	795	II	1N non-vacuolated
175	270	445	670	II	1N non-vacuolated
175	290	465	750	II	1N non-vacuolated
175	275	450	705	II	1N non-vacuolated
160	260	420	665	II	1N non-vacuolated
180	290	470	805	II	1N non-vacuolated
180	275	455	770	II	1N non-vacuolated
190	260	450	650	II	1N non-vacuolated
165	285	450	650	II	1N non-vacuolated
180	305	485	830	II	1N non-vacuolated
215	350	565	1000	II	1N vacuolated
220	430	650	1010	III	1N vacuolated
225	430	655	1025	III	1N vacuolated
245	505	750	1105	IV	1N vacuolated
160	370	530	800	II	1N vacuolated
220	480	700	1080	III	1N vacuolated
220	435	655	1100	III	1N vacuolated
240	480	720	1035	IV	1N vacuolated
190	330	520	875	II	1N vacuolated
240	565	805	1120	IV	1N vacuolated
185	315	500	910	II	1N vacuolated
230	475	705	1040	III	1N vacuolated
255	513	768	1090	IV	1N vacuolated
190	310	500	880	II	1N vacuolated
245	495	740	1095	IV	1N vacuolated
185	310	495	845	II	1N vacuolated
170	390	560	950	II	1N vacuolated
230	460	690	1075	III	1N vacuolated
225	375	600	1045	III	1N vacuolated
247	476	723	1205	IV	1N vacuolated
197	365	562	1030	II	1N vacuolated
235	465	700	1042	III	1N vacuolated
190	340	530	795	II	1N vacuolated
220	450	670	1055	III	1N vacuolated
235	470	705	1060	III	1N vacuolated
236	526	762	1060	III	1N vacuolated
235	455	690	1010	III	1N vacuolated
260	485	745	1130	IV	1N vacuolated
190	365	555	845	II	1N vacuolated
230	450	680	1030	III	1N vacuolated
235	465	700	1070	III	1N vacuolated
270	570	840	1215	IV	2N

255	635	890	1250	IV	2N
280	710	990	1240	IV	2N
260	645	905	1260	IV	2N
290	685	975	1240	IV	2N
270	630	900	1240	IV	2N
260	680	940	1210	IV	2N
285	715	1000	1270	IV	2N
280	660	940	1270	IV	2N
270	590	860	1275	IV	2N
275	690	965	1225	IV	2N
90	60	150	145	I	Arch
125	130	255	305	I	Arch
105	85	190	215	I	Arch
123	163	288	305	I	Arch
100	90	190	200	I	Arch
120	115	235	250	I	Arch
95	85	180	175	I	Arch
120	125	245	285	I	Arch
100	85	185	175	I	Arch
105	85	190	190	I	Arch
90	55	145	140	I	Arch
110	100	210	205	I	Arch
136	164	300	345	I	MiMC
145	205	350	455	II	MiMC
140	187	327	400	II	MiMC
132	175	307	390	I	MiMC
145	185	330	385	II	MiMC
132	195	327	355	II	MiMC
115	125	240	245	I	MiMC
110	140	250	255	I	MiMC
320	745	1065	1405	IV	Pollen
300	730	1030	1315	IV	Pollen
315	885	1200	1410	IV	Pollen
315	855	1170	1430	IV	Pollen
320	755	1075	1405	IV	Pollen
265	775	1040	1345	IV	Pollen
315	965	1280	1490	IV	Pollen
310	775	1085	1485	IV	Pollen
285	920	1205	1500	IV	Pollen
330	820	1150	1480	IV	Pollen
265	665	930	1265	IV	Pollen
325	880	1205	1485	IV	Pollen
300	700	1000	1325	IV	Pollen
325	770	1095	1475	IV	Pollen
335	920	1255	1465	IV	Pollen

	285	785	1070	1350	IV	Pollen
	330	910	1240	1470	IV	Pollen
	280	725	1005	1275	IV	Pollen
	315	760	1075	1400	IV	Pollen
	305	715	1020	1455	IV	Pollen
	330	805	1135	1485	IV	Pollen
	305	815	1120	1310	IV	Pollen
	340	875	1215	1500	IV	Pollen
	280	670	950	1250	IV	Pollen
	330	925	1255	1470	IV	Pollen
	270	775	1045	1270	IV	Pollen
	320	865	1185	1455	IV	Pollen
	325	950	1275	1490	IV	Pollen
	280	670	950	1345	IV	Pollen
	310	830	1140	1430	IV	Pollen
	335	910	1245	1520	IV	Pollen
	275	755	1030	1305	IV	Pollen
**$\bar{x}$**	226	490	716	978		
**n**	108	108	108	108		

*Genotypes: DL (Don Luis), DP (Don Pablo), DW (Don Walter), TU (Tanganyika), OTA (OTA-S), CAT (Catalina) and PI9 (PI299920). Parameters: pistil length (PL), ovary length (OL), style length (SL), and anther length (AL). Female developmental stages: I: Megaspore Mother Cell, II: Postmeiosis or EMMC, III: Immature embryo sac, and IV: Mature embryo sac. Male development stages: Arch (Archesporial pollen cells), MiMC (Microspore mother cell), meiosis, 1N NV (1N Non-vacuolated), 1N V (1N Vacuolated), 2N (Bicellular pollen grain) and Pollen).
