# Supplementary material for: A Precise Reproductive Calendar of Sexual and Apomictic Genotypes of Eragrostis curvula
Source: Plants (Basel). 2026 Mar 29;15(7):1050. doi: 10.3390/plants15071050 (PMC13074311; doi:10.3390/plants15071050)
Supplement: Supplementary file 1 [file plants-15-01050-s001.zip › supplementary material/Figure S5. SL Genotypes.pdf]

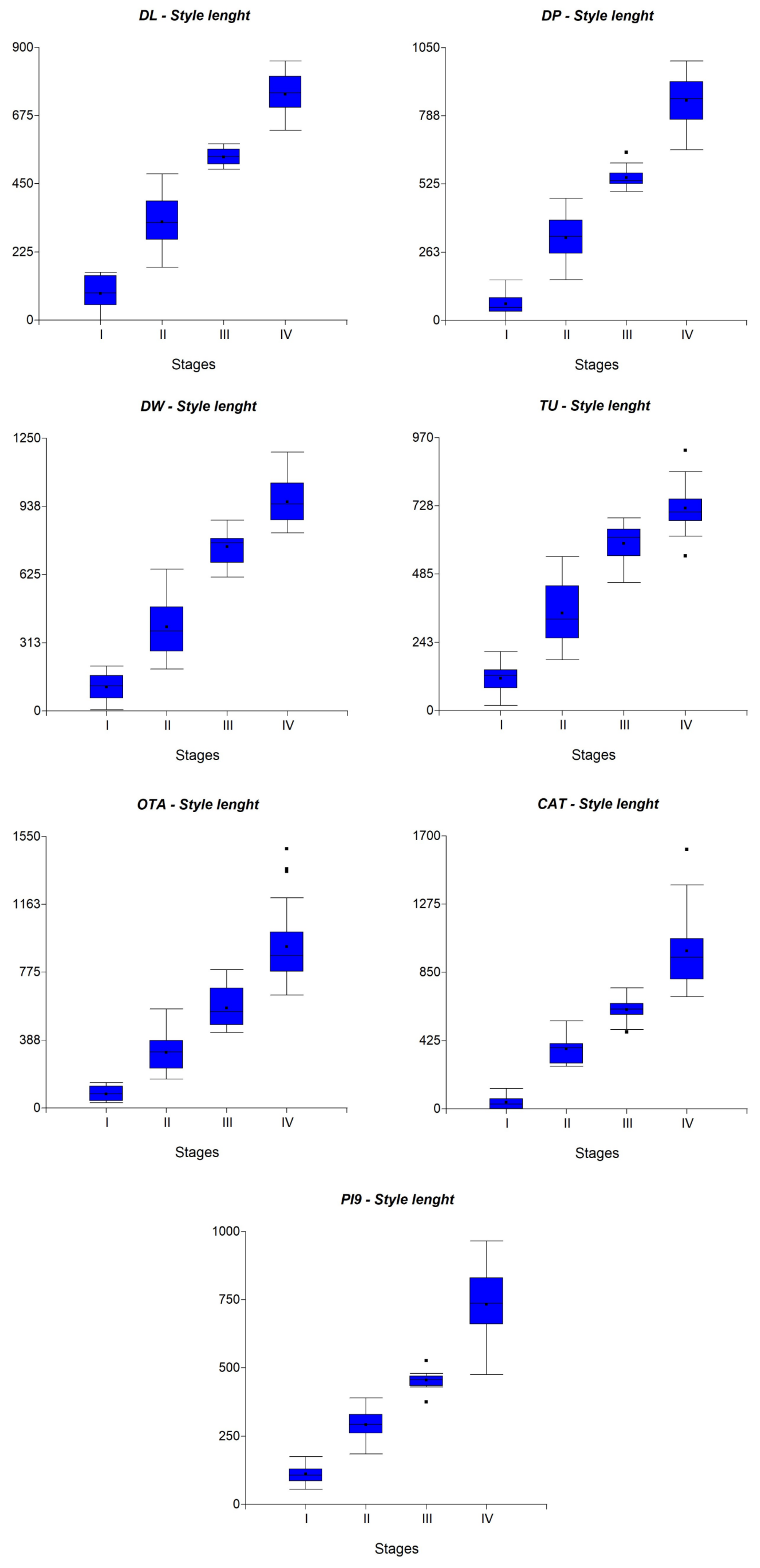
DL - Style lenght
900
675
450
225
0
I
II
III
IV
Stages
DP - Style lenght
1050
788
525
263
0
I
II
III
IV
Stages
DW - Style lenght
1250
938
625
313
0
I
II
III
IV
Stages
TU - Style lenght
970
728
485
243
0
I
II
III
IV
Stages
OTA - Style lenght
1550
1163
775
388
0
I
II
III
IV
Stages
CAT - Style lenght
1700
1275
850
425
0
I
II
III
IV
Stages
PI9 - Style lenght
1000
750
500
250
0
I
II
III
IV
Stages
