# Supplementary material for: A Precise Reproductive Calendar of Sexual and Apomictic Genotypes of Eragrostis curvula
Source: Plants (Basel). 2026 Mar 29;15(7):1050. doi: 10.3390/plants15071050 (PMC13074311; doi:10.3390/plants15071050)
Supplement: Supplementary file 1 [file plants-15-01050-s001.zip › supplementary material/Table S3. Interval table generated from the 50th and 75th percentiles.pdf]

Table S3. Interval table generated from the 50th and 75th percentiles. Table shows for each stage and genotype the low, high and center values of each parameter.

Percentile	Genotype	Stage	Parameter	Low (μm)	High (μm)	Center (μm)
**50**	**DL**	I	OL	105	133	119
			SL	52	144	98
			PL	157	274	215
			AL	170	336	253
		II	OL	184	227	205
			SL	266	392	329
			PL	447	621	534
			AL	587	868	727
		III	OL	271	292	281
			SL	517	555	536
			PL	805	843	824
			AL	1007	1058	1032
		IV	OL	312	328	320
			SL	702	805	754
			PL	1023	1117	1070
			AL	1082	1162	1122
	**DP**	I	OL	92	113	102
			SL	36	89	62
			PL	126	197	161
			AL	148	218	183
		II	OL	182	234	208
			SL	261	386	323
			PL	445	614	530
			AL	559	782	671
		III	OL	271	289	280
			SL	526	567	546
			PL	801	855	828
			AL	949	1000	974
		IV	OL	322	364	343
			SL	774	918	846
			PL	1103	1262	1182
			AL	1100	1274	1187
	**DW**	I	OL	95	128	111
			SL	62	160	111
			PL	154	287	221
			AL	168	285	226
		II	OL	164	224	194
			SL	276	469	373
			PL	439	702	570
			AL	528	892	710

	III	OL	282	298	290
		SL	691	794	743
		PL	981	1087	1034
		AL	1075	1179	1127
	IV	OL	316	349	332
		SL	875	1042	958
		PL	1203	1375	1289
		AL	1198	1288	1243
**TU**	I	OL	100	127	113
		SL	80	145	113
		PL	180	280	230
		AL	196	326	261
	II	OL	170	225	198
		SL	257	444	351
		PL	435	670	553
		AL	550	735	643
	III	OL	250	280	265
		SL	555	643	599
		PL	825	917	871
		AL	784	941	862
	IV	OL	294	332	313
		SL	675	752	714
		PL	975	1071	1023
		AL	950	1060	1005
**OTA**	I	OL	123	153	138
		SL	43	123	83
		PL	165	270	218
		AL	185	328	256
	II	OL	200	240	220
		SL	233	386	309
		PL	424	615	519
		AL	634	890	762
	III	OL	265	300	283
		SL	488	665	576
		PL	738	958	848
		AL	978	1130	1054
	IV	OL	325	365	345
		SL	780	1005	893
		PL	1100	1330	1215
		AL	1250	1390	1320
**CAT**	I	OL	73	106	89
		SL	5	58	31
		PL	84	163	124

			AL	82	137	110
		II	OL	176	215	196
			SL	288	409	348
			PL	465	620	542
			AL	737	878	808
		III	OL	240	256	248
			SL	586	656	621
			PL	831	907	869
			AL	980	1008	994
		IV	OL	291	323	307
			SL	811	1071	941
			PL	1110	1391	1250
			AL	1055	1113	1084
	**PI9**	I	OL	100	121	110
			SL	85	133	109
			PL	189	251	220
			AL	186	290	238
		II	OL	160	186	173
			SL	260	333	296
			PL	439	521	480
			AL	654	845	749
		III	OL	221	235	228
			SL	439	469	454
			PL	659	700	679
			AL	1033	1068	1050
		IV	OL	270	319	294
			SL	661	828	744
			PL	933	1148	1040
			AL	1243	1463	1353
**75**	**DL**	I	OL	86	142	114
			SL	10	154	82
			PL	96	297	196
			AL	111	383	247
		II	OL	171	238	204
			SL	203	433	318
			PL	374	672	523
			AL	505	922	713
		III	OL	267	295	281
			SL	508	573	540
			PL	784	866	825
			AL	980	1065	1022
		IV	OL	305	340	322
			SL	657	826	741

		PL	969	1163	1066
		AL	1080	1186	1133
**DP**	I	OL	84	130	107
		SL	29	110	69
		PL	116	240	178
		AL	111	257	184
	II	OL	166	251	209
		SL	206	410	308
		PL	376	647	511
		AL	468	854	661
	III	OL	264	294	279
		SL	506	605	555
		PL	776	892	834
		AL	941	1016	979
	IV	OL	313	383	348
		SL	741	954	848
		PL	1027	1307	1167
		AL	1056	1319	1187
**DW**	I	OL	89	130	110
		SL	39	175	107
		PL	121	304	213
		AL	128	315	221
	II	OL	147	242	194
		SL	241	559	400
		PL	405	807	606
		AL	469	990	729
	III	OL	277	305	291
		SL	649	827	738
		PL	930	1132	1031
		AL	1038	1206	1122
	IV	OL	309	372	340
		SL	852	1080	966
		PL	1179	1437	1308
		AL	1144	1311	1228
**TU**	I	OL	93	144	118
		SL	54	161	107
		PL	154	309	231
		AL	166	379	273
	II	OL	162	243	202
		SL	230	508	369
		PL	402	745	574
		AL	500	872	686
	III	OL	247	285	266

		SL	486	673	580
		PL	732	940	836
		AL	765	984	874
	IV	OL	283	343	313
		SL	658	819	738
		PL	958	1138	1048
		AL	923	1144	1033
**OTA**	I	OL	119	163	141
		SL	40	130	85
		PL	161	293	227
		AL	179	386	283
	II	OL	178	250	214
		SL	195	424	310
		PL	380	679	529
		AL	507	980	743
	III	OL	250	304	277
		SL	441	695	568
		PL	705	999	852
		AL	943	1228	1085
	IV	OL	315	390	353
		SL	715	1155	935
		PL	1045	1540	1293
		AL	1200	1420	1310
**CAT**	I	OL	57	120	89
		SL	0	87	43
		PL	65	206	136
		AL	60	221	141
	II	OL	168	219	193
		SL	281	449	365
		PL	445	671	558
		AL	682	885	784
	III	OL	238	265	251
		SL	536	700	618
		PL	782	958	870
		AL	976	1032	1004
	IV	OL	281	346	314
		SL	748	1319	1033
		PL	1034	1652	1343
		AL	1039	1131	1085
**PI9**	I	OL	94	126	110
		SL	82	163	123
		PL	176	290	233
		AL	171	310	241

II	OL	151	190	170
	SL	222	359	291
	PL	374	546	460
	AL	511	899	705
III	OL	220	235	228
	SL	430	477	453
	PL	653	705	679
	AL	1019	1077	1048
IV	OL	256	329	293
	SL	566	907	736
	PL	809	1214	1012
	AL	1206	1485	1345

Genotypes: DL (Don Luis), DP (Don Pablo), DW (Don Walter), TU (Tanganyika), OTA (OTA-S), CAT (Catalina) and PI9 (PI299920). Female developmental stages: I: Megaspore Mother Cell, II: Postmeiosis or EMMC, III: Immature embryo sac, and IV: Mature embryo sac. Parameters: pistil length (PL), ovary length (OL), style length (SL), and anther length (AL).
