# Supplementary material for: A Precise Reproductive Calendar of Sexual and Apomictic Genotypes of Eragrostis curvula
Source: Plants (Basel). 2026 Mar 29;15(7):1050. doi: 10.3390/plants15071050 (PMC13074311; doi:10.3390/plants15071050)
Supplement: Supplementary file 1 [file plants-15-01050-s001.zip › supplementary material/Supplementary figure leyends.pdf]

Supplementary figure leyends

Figure S1: Schematic representation of panicle and spikelet morphology of *E. curvula*. (a) Panicle diagram with arrows indicating the maturation gradient, where florets in the upper portion of the inflorescence are the most advanced in development. (b) Detailed illustration of a spikelet showing six visible anthecia (b-1 to b-6), where the basal anthecium (b-1) is the most mature and typically reaches anthesis first

Figure S2: Number of anthecia per spikelet in seven *E. curvula* genotypes. Bars represent mean values ± standard deviation. Genotypes: DL: Don Luis, DP: Don Pablo, DW: Don Walter, TU: Tanganyika, OTA: OTA-S, CAT: Catalina, PI9: PI299920.

Figure S3: Spikelet morphology of OTA-S genotype. (a) Intact spikelet showing the characteristic reduced number of anthecia. (b) Dissected spikelet revealing the aborted apical anthecia (arrow).

Figure S4: Box-plot of ovary length for all female developmental stages and *E. curvula* genotypes. (a) PI9: PI299920, (b) OTA: OTA-S, (c) TU: Tanganyika, (d) DW: Don Walter, (e) CAT: Catalina, (f) DL: Don Luis, (g) DP: Don Pablo. Female developmental stages: I: Megaspore mother cell, II: Postmeiosis or EMMC, III: Immature embryo sac, and IV: Mature embryo sac.

Figure S5: Box-plot of style length for all female developmental stages and *E. curvula* genotypes. (a) PI9: PI299920, (b) OTA: OTA-S, (c) TU: Tanganyika, (d) DW: Don Walter, (e) CAT: Catalina, (f) DL: Don Luis, (g) DP: Don Pablo. Female developmental stages: I: Megaspore mother cell, II: Postmeiosis or EMMC, III: Immature embryo sac, and IV: Mature embryo sac.

Figure S6: Box-plot of anther length for all female developmental stages and *E. curvula* genotypes. (a) PI9: PI299920, (b) OTA: OTA-S, (c) TU: Tanganyika, (d) DW: Don Walter, (e) CAT: Catalina, (f) DL: Don Luis, (g) DP: Don Pablo. Female developmental stages: I: Megaspore mother cell, II: Postmeiosis or EMMC, III: Immature embryo sac, and IV: Mature embryo sac. Figure S7: Ovary length in all stages

Figure S7: Ovary length for all genotypes, discriminated by female developmental stage. (a) Stages: I: Megaspore mother cell, (b) Stage II: Postmeiosis or EMMC, (c) Stage III: Immature embryo sac, (d) Stage IV: Mature embryo sac. Genotypes: DL: Don Luis, DP: Don Pablo, DW: Don Walter, TU: Tanganyika, OTA: OTA-S, CAT: Catalina, and PI9: PI299920

Figure S8: Style length for all genotypes, discriminated by female developmental stage. (a) Stages: I: Megaspore mother cell, (b) Stage II: Postmeiosis or EMMC, (c) Stage III: Immature embryo sac, (d) Stage IV: Mature embryo sac. Genotypes: DL: Don Luis, DP: Don Pablo, DW: Don Walter, TU: Tanganyika, OTA: OTA-S, CAT: Catalina, and PI9: PI299920

Figure S9: Anther length for all genotypes, discriminated by female developmental stage. (a) Stages: I: Megaspore mother cell, (b) Stage II: Postmeiosis or EMMC, (c) Stage

III: Immature embryo sac, (d) Stage IV: Mature embryo sac. Genotypes: DL: Don Luis, DP: Don Pablo, DW: Don Walter, TU: Tanganyika, OTA: OTA-S, CAT: Catalina, and PI9: PI299920

Figure S10: Parameters. Measurement of floral parameters: AL (anther length), PL (pistil length), OL (ovary length), and SL (style length)
