# Supplementary material for: A Precise Reproductive Calendar of Sexual and Apomictic Genotypes of Eragrostis curvula
Source: Plants (Basel). 2026 Mar 29;15(7):1050. doi: 10.3390/plants15071050 (PMC13074311; doi:10.3390/plants15071050)
Supplement: Supplementary file 1 [file plants-15-01050-s001.zip › supplementary material/Table S5. Predictions generated from the 50th and 75th percentiles.pdf]

Table S5. Predictions generated from the 50th and 75th percentiles to assess the classification accuracy of each parameter.

Percentile	Genotype	OL (µm)	SL (µm)	PL (µm)	AL (µm)	Stage	Pred OL	Pred SL	Pred PL	Pred AL
50	DL	120	149	269	353	I	I		I	
		121	65	186	216	I	I	I	I	I
		80	0	80	110	I				
		108	86	194	226	I	I	I	I	I
		65	10	75	70	I				
		118	107	224	284	I	I	I	I	I
		104	0	104	114	I				
		122	87	209	315	I	I	I	I	I
		125	104	229	263	I	I	I	I	I
		75	15	90	112	I				
		142	133	275	324	I		I		I
		117	92	208	269	I	I	I	I	I
		124	123	247	302	I	I	I	I	I
		90	10	100	110	I				
		147	153	300	410	I				
		105	50	155	190	I	I			I
		105	56	161	199	I	I	I	I	I
		141	158	299	469	I				
		153	155	308	388	I				
		136	158	295	380	I				
		109	61	170	163	I	I	I	I	
		143	147	291	340	I				
		219	340	559	736	II	II	II	II	II
		170	218	388	553	II				
		221	432	653	922	II	II			
		207	337	545	730	II	II	II	II	II
		162	204	366	494	II				
		250	446	696	926	II				
		216	388	603	785	II	II	II	II	II
		188	271	458	628	II	II	II	II	II
		147	185	332	398	II				
		237	380	618	858	II		II	II	II
		213	322	535	712	II	II	II	II	II
		174	195	368	496	II				
		226	378	604	876	II	II	II	II	
		200	328	528	732	II	II	II	II	II
		235	364	599	900	II		II	II	
		212	315	527	723	II	II	II	II	II
		185	191	376	530	II	II			
		255	449	704	961	II				

227	400	627	842	II				II
192	304	496	663	II	II	II	II	II
158	198	356	421	II				
230	401	631	906	II				
237	393	630	771	II				II
225	458	683	966	II	II			
213	305	518	859	II	II	II	II	II
180	266	446	686	II		II		II
170	203	373	463	II				
221	381	602	887	II	II	II	II	
210	352	562	740	II	II	II	II	II
179	230	409	553	II				
240	439	679	927	II				
203	313	516	797	II	II	II	II	II
181	266	447	592	II		II	II	II
238	413	651	937	II				
202	346	548	807	II	II	II	II	II
183	239	422	603	II				II
159	177	336	400	II				
245	433	678	831	II				II
173	292	465	620	II		II	II	II
233	391	624	909	II		II		
218	398	616	793	II	II		II	II
198	269	467	582	II	II	II	II	
165	174	339	414	II				
254	483	737	956	II				
221	383	604	808	II	II	II	II	II
186	237	423	582	II	II			
261	462	723	920	II				
192	312	503	696	II	II	II	II	II
190	278	468	618	II	II	II	II	II
191	302	493	760	II	II	II	II	II
197	273	470	540	II	II	II	II	
298	573	871	1042	III				III
279	506	786	1031	III	III			III
271	545	816	1055	III	III	III	III	III
271	540	811	980	III	III	III	III	
293	573	866	1064	III				
297	515	813	1008	III			III	III
294	546	841	1112	III		III	III	IV
268	508	776	1039	III				III
265	545	810	980	III		III	III	
271	537	808	1052	III	III	III	III	III
262	498	760	1005	III				
280	564	844	977	III	III			

	290	523	812	1060	III	III	III	III	
	286	582	867	1066	III	III			
	281	519	801	1011	III	III	III		III
	339	855	1193	1231	IV				
	341	658	999	1167	IV				
	305	656	961	1086	IV				IV
	323	815	1137	1156	IV	IV			IV
	312	718	1030	1079	IV	IV	IV	IV	
	347	836	1184	1187	IV				
	321	758	1078	1080	IV	IV	IV	IV	
	326	781	1107	1124	IV	IV	IV	IV	IV
	326	702	1028	1080	IV	IV	IV	IV	
	302	721	1023	1118	IV		IV	IV	IV
	321	753	1074	1112	IV	IV	IV	IV	IV
	320	797	1117	1162	IV	IV	IV	IV	IV
	319	658	976	1076	IV	IV			
	304	642	946	1077	IV				
	298	814	1112	1114	IV			IV	IV
	336	805	1141	1199	IV		IV		
	328	627	955	1092	IV	IV			IV
	341	845	1186	1131	IV				IV
	325	747	1072	1082	IV	IV	IV	IV	IV
	311	750	1061	1185	IV		IV	IV	
	326	710	1036	1112	IV	IV	IV	IV	IV
DP	94	46	140	166	I	I	I	I	I
	124	67	191	212	I		I	I	I
	129	106	235	237	I				
	81	36	117	100	I		I		
	92	0	92	95	I	I			
	107	34	142	160	I	I		I	I
	90	29	119	113	I				
	109	87	196	248	I	I	I	I	
	137	140	277	342	I				
	138	155	293	316	I				
	81	48	128	152	I		I	I	I
	102	96	198	161	I	I			I
	84	26	110	137	I				
	96	39	136	153	I	I	I	I	I
	109	51	160	160	I	I	I	I	I
	105	61	166	158	I	I	I	I	I
	222	394	615	889	II	II			
	156	181	337	440	II				
	222	405	627	874	II	II			
	208	324	532	702	II	II	II	II	II

153	200	353	465	II				
251	445	696	891	II				
178	285	462	692	II		II	II	II
161	193	354	461	II				
251	435	686	813	II				
196	258	454	632	II	II		II	II
154	157	311	370	II				
219	410	629	961	II	II			III
236	372	608	785	II		II	II	
193	287	479	591	II	II	II	II	II
217	301	518	752	II	II	II	II	II
174	222	396	492	II				
199	362	560	719	II	II	II	II	II
215	327	542	723	II	II	II	II	II
164	210	374	434	II				
261	418	678	850	II				
213	310	524	665	II	II	II	II	II
237	373	610	728	II		II	II	II
190	253	443	516	II	II			
226	335	561	701	II	II	II	II	II
169	199	369	444	II				
257	432	689	861	II				
236	319	555	663	II		II	II	II
218	309	528	690	II	II	II	II	II
229	410	640	850	II	II			
211	293	504	651	II	II	II	II	II
151	177	328	425	II				
247	397	644	769	II				II
220	369	589	826	II	II	II	II	
224	299	523	737	II	II	II	II	II
218	370	588	750	II	II	II	II	II
172	258	431	535	II				
209	403	612	770	II	II		II	II
166	204	370	551	II				
223	355	578	742	II	II	II	II	II
175	268	443	558	II		II		
263	408	671	854	II				
202	325	527	661	II	II	II	II	II
166	222	388	445	II				
223	359	581	754	II	II	II	II	II
194	205	399	570	II	II			II
180	253	432	563	II				II
250	445	695	885	II				
212	283	496	665	II	II	II	II	II
253	386	638	734	II				II

189	268	456	563	II	II	II	II	II
276	470	746	852	II	III			
203	324	528	620	II	II	II	II	II
241	339	580	772	II		II	II	II
179	272	451	517	II		II	II	
247	399	646	811	II				
194	276	470	550	II	II	II	II	
262	385	647	903	II		II		
223	326	548	700	II	II	II	II	II
290	527	816	1019	III		III	III	
274	530	804	997	III	III	III	III	III
266	496	762	991	III				III
259	525	784	941	III				
253	568	821	952	III			III	III
280	606	886	1005	III	III			
272	548	821	960	III	III	III	III	III
270	505	775	961	III				III
289	511	800	943	III	III			
276	536	812	1000	III	III	III	III	
273	539	812	930	III	III	III	III	
295	605	899	994	III				III
264	547	811	936	III		III	III	
296	532	828	1048	III		III	III	
289	605	893	981	III	III			III
273	500	772	948	III	III			
300	563	864	1018	III		III		
284	647	931	999	III	III			III
384	999	1383	1328	IV				
345	906	1252	1281	IV	IV	IV	IV	
342	988	1329	1200	IV	IV			IV
384	916	1300	1335	IV		IV		
355	834	1189	1138	IV	IV	IV	IV	IV
315	793	1108	1111	IV		IV	IV	IV
357	848	1205	1278	IV	IV	IV	IV	
304	657	961	1016	IV				
348	869	1217	1151	IV	IV	IV	IV	IV
332	919	1251	1132	IV	IV		IV	IV
289	760	1049	1026	IV	III			
324	762	1086	1100	IV	IV			IV
333	938	1271	1104	IV	IV			IV
349	854	1202	1092	IV	IV	IV	IV	
285	735	1020	1046	IV	III			
334	808	1142	1100	IV	IV	IV	IV	IV
325	773	1098	1085	IV	IV			
312	657	969	999	IV				III

	394	830	1224	1270	IV		IV	IV	IV
	379	894	1273	1292	IV		IV		
	338	775	1150	1248	IV	IV	IV	IV	IV
	320	881	1201	1150	IV		IV	IV	IV
	360	959	1200	1234	IV	IV		IV	IV
	315	689	1004	1116	IV				IV
	369	940	1309	1210	IV				IV
	368	878	1246	1340	IV		IV	IV	
	411	993	1404	1367	IV				
DW	105	89	194	179	I	I	I	I	I
	116	140	257	254	I	I	I	I	I
	84	58	143	138	I				
	112	127	240	221	I	I	I	I	I
	79	39	118	108	I				
	107	104	211	167	I	I	I	I	
	89	5	89	105	I				
	133	179	312	250	I				I
	77	19	96	114	I				
	125	141	266	334	I	I	I	I	
	99	99	198	205	I	I	I	I	I
	129	171	300	284	I				I
	130	177	307	316	I				
	104	75	178	171	I	I	I	I	I
	68	5	68	80	I				
	128	177	305	273	I				I
	92	40	131	148	I				
	125	165	290	281	I	I			I
	99	51	150	149	I	I			
	105	134	239	263	I	I	I	I	I
	83	5	83	109	I				
	99	153	252	258	I	I	I	I	I
	89	31	120	126	I				
	132	205	337	303	I				
	92	73	165	176	I		I	I	I
	130	125	255	290	I		I	I	
	105	45	150	170	I	I			I
	107	96	203	230	I	I	I	I	I
	128	167	295	321	I				
	126	162	288	297	I	I			
	92	55	147	154	I				
	135	175	310	315	I				
	95	80	175	175	I	I	I	I	I
	130	155	285	305	I		I	I	
	130	115	245	285	I		I	I	I

95	80	175	165	I	I	I	I	
120	120	240	240	I	I	I	I	I
120	135	255	265	I	I	I	I	I
95	108	203	226	I	I	I	I	I
125	155	280	285	I	I	I	I	I
140	190	330	365	I				
120	115	235	225	I	I	I	I	I
130	170	300	320	I				
73	36	109	117	I				
130	165	295	315	I				
115	95	210	240	I	I	I	I	I
105	125	230	240	I	I	I	I	I
120	80	200	245	I	I	I	I	I
100	50	150	190	I	I			I
126	179	305	318	I	I			
251	563	813	1050	II				
175	361	536	628	II	II	II	II	II
145	211	357	351	II				
281	650	931	1117	II				III
243	595	837	962	II				
190	354	544	706	II	II	II	II	II
144	265	409	468	II				
273	637	910	1134	II				III
233	588	821	992	II				
206	416	622	743	II	II	II	II	II
206	415	621	677	II	II	II	II	II
147	234	381	404	II				
195	508	703	892	II	II			
228	477	706	891	II				II
189	366	555	620	II	II	II	II	II
263	548	811	978	II				
185	461	646	781	II	II	II	II	II
191	393	584	832	II	II	II	II	II
178	319	497	577	II	II	II	II	II
222	486	708	894	II	II			
174	342	516	611	II	II	II	II	II
141	227	368	369	II				
256	584	840	1007	II				
190	356	546	787	II	II	II	II	II
162	292	454	535	II		II	II	II
171	279	451	552	II	II	II	II	II
226	590	816	997	II				
194	401	595	764	II	II	II	II	II
148	236	384	492	II				
205	428	633	787	II	II	II	II	II

161	288	448	516	II		II	II	
247	514	761	982	II				
202	402	605	742	II	II	II	II	II
156	270	426	471	II				
232	562	794	1015	II				
205	434	639	753	II	II	II	II	II
148	273	421	496	II				
206	441	647	1030	II	II	II	II	
193	422	615	822	II	II	II	II	II
171	307	478	570	II	II	II	II	II
158	249	407	649	II				II
210	455	665	965	II	II	II	II	
195	350	545	670	II	II	II	II	II
165	240	405	485	II	II			
249	454	703	893	II		II		
212	308	520	628	II	II	II	II	II
143	192	335	362	II				
231	507	738	845	II				II
168	285	453	558	II	II	II	II	II
226	446	672	754	II		II	II	II
185	245	430	525	II	II			
237	403	640	877	II		II	II	II
175	300	475	530	II	II	II	II	II
192	314	506	650	II	II	II	II	II
145	260	405	430	II				
144	206	350	376	II				
145	240	385	450	II				
217	483	700	765	II	II		II	II
155	255	410	485	II				
286	874	1160	1296	III	III			
309	774	1083	1214	III		III	III	IV
275	680	955	1077	III				III
305	853	1158	1247	III				IV
292	802	1094	1157	III	III			III
280	663	943	1012	III				
283	775	1058	1137	III	III	III	III	III
294	767	1061	1193	III	III	III	III	
283	651	934	1082	III	III			III
291	767	1058	1136	III	III	III	III	III
308	819	1128	1205	III				IV
291	779	1070	1112	III	III	III	III	III
277	751	1028	1088	III		III	III	III
279	613	892	1012	III				
284	823	1106	1200	III	III			IV
298	780	1077	1121	III	III	III	III	III

	286	764	1051	1136	III	III	III	III	III
	290	791	1081	1174	III	III	III	III	III
	308	776	1084	1042	III		III	III	
	295	695	990	1100	III	III	III	III	III
	305	724	1029	1091	III		III	III	III
	274	629	903	1068	III				
	298	872	1170	1067	III	III			
	253	637	890	941	III				
	346	1187	1533	1320	IV	IV			
	290	816	1106	1091	IV	III			III
	308	948	1256	1313	IV		IV	IV	
	329	874	1202	1198	IV	IV			IV
	315	900	1215	1363	IV		IV	IV	
	330	845	1175	1280	IV	IV			IV
	378	954	1332	1285	IV		IV	IV	IV
	331	1045	1377	1306	IV	IV			
	311	892	1203	1229	IV		IV	IV	IV
	330	962	1292	1230	IV	IV	IV	IV	IV
	301	858	1159	1240	IV				IV
	340	1064	1404	1290	IV	IV			
	334	1039	1373	1198	IV	IV	IV	IV	IV
	316	875	1191	1208	IV	IV	IV		IV
	351	960	1311	1239	IV		IV	IV	IV
	338	945	1283	1146	IV	IV	IV	IV	III
	449	850	1299	1211	IV			IV	IV
	375	1101	1476	1143	IV				III
	363	1085	1448	1088	IV				III
OTA	135	80	215	270	I	I	I	I	I
	125	45	170	175	I	I	I	I	
	175	145	320	390	I				
	155	80	235	240	I		I	I	I
	115	50	165	190	I		I	I	I
	105	40	145	140	I				
	120	30	150	180	I				
	140	90	230	225	I	I	I	I	I
	150	125	275	335	I	I			
	145	120	265	320	I	I	I	I	I
	135	40	175	245	I	I		I	I
	170	130	300	400	I				
	160	130	290	385	I				
	120	45	165	180	I		I	I	
	125	40	165	195	I	I		I	I
	260	565	825	1090	II		III	III	III
	265	455	720	1010	II	III			III

225	325	550	750	II	II	II	II	II
214	271	485	750	II	II	II	II	II
240	400	640	870	II	II			II
160	165	325	475	II				
250	455	705	1000	II				III
200	225	425	740	II	II		II	II
205	265	470	780	II	II	II	II	II
230	370	600	880	II	II	II	II	II
180	215	395	510	II				
220	410	630	950	II	II			
190	190	380	650	II				II
260	430	690	995	II				III
230	380	610	810	II	II	II	II	II
160	195	355	410	II				
230	280	510	690	II	II	II	II	II
255	405	660	955	II				
205	260	465	680	II	II	II	II	II
205	250	455	635	II	II	II	II	II
215	310	525	730	II	II	II	II	II
215	300	515	680	II	II	II	II	II
211	259	470	680	II	II	II	II	II
200	220	420	640	II	II			II
230	390	620	890	II	II			II
170	190	360	500	II				
230	370	600	890	II	II	II	II	II
170	210	380	540	II				
240	355	595	885	II	II	II	II	II
240	325	565	795	II	II	II	II	II
200	415	615	955	II	II		II	
200	175	375	500	II	II			
170	190	360	415	II				
225	325	550	685	II	II	II	II	II
190	180	370	540	II				
250	350	600	705	II		II	II	II
210	315	525	640	II	II	II	II	II
205	245	450	580	II	II	II	II	
240	390	630	910	II	II			
220	355	575	715	II	II	II	II	II
176	214	390	500	II				
240	345	585	810	II	II	II	II	II
225	235	460	630	II	II	II	II	
200	330	530	660	II	II	II	II	II
165	240	405	520	II		II		
285	525	810	1145	II	III	III	III	
210	365	575	870	II	II	II	II	II

210	195	405	505	II	II			
220	300	520	670	II	II	II	II	II
250	480	730	935	II				
230	385	615	1035	II	II	II	II	III
275	430	705	1130	II	III			III
270	550	820	1020	III	III	III	III	III
300	705	1005	1135	III	III			
245	475	720	1020	III				III
325	695	1020	1100	III	IV			III
275	515	790	1050	III	III	III	III	III
300	580	880	1065	III	III	III	III	III
285	445	730	895	III	III			
240	440	680	980	III	II			III
305	790	1095	1125	III		IV		III
285	695	980	1175	III	III			
245	500	745	1115	III		III	III	III
265	685	950	1100	III	III		III	III
265	530	795	950	III	III	III	III	
265	535	800	975	III	III	III	III	
265	435	700	795	III	III			II
265	430	695	940	III	III			
320	645	965	1280	III		III		IV
300	595	895	1245	III	III	III	III	
295	580	875	1265	III	III	III	III	IV
385	1155	1540	1420	IV				
370	1010	1380	1370	IV				IV
315	1025	1340	1185	IV				
325	1005	1330	1265	IV	IV	IV	IV	IV
340	1025	1365	1240	IV	IV			
360	730	1090	1240	IV	IV			
335	705	1040	1150	IV	IV			
325	840	1165	1300	IV	IV	IV	IV	IV
295	780	1075	1200	IV	III	IV		
345	815	1160	1350	IV	IV	IV	IV	IV
325	835	1160	1330	IV	IV	IV	IV	IV
325	645	970	1160	IV	IV	III		
315	690	1005	1190	IV				
415	1365	1780	1660	IV				
350	970	1320	1325	IV	IV	IV	IV	IV
355	925	1280	1330	IV	IV	IV	IV	IV
330	940	1270	1370	IV	IV	IV	IV	IV
415	1200	1615	1500	IV				
315	960	1275	1345	IV		IV	IV	IV
320	815	1135	1250	IV		IV	IV	IV
390	1350	1740	1560	IV				

	350	870	1220	1295	IV	IV	IV	IV	IV
	430	1480	1910	1580	IV				
	380	820	1200	1240	IV		IV	IV	
	395	790	1185	1400	IV		IV	IV	
	305	975	1280	1310	IV		IV	IV	IV
	345	985	1330	1380	IV	IV	IV	IV	IV
	360	920	1280	1405	IV	IV	IV	IV	
	310	690	1000	1335	IV				IV
	315	745	1060	1390	IV				IV
	335	765	1100	1300	IV	IV		IV	IV
	365	825	1190	1310	IV	IV	IV	IV	IV
	330	715	1045	1400	IV	IV			
TU	130	210	340	360	I				
	130	210	340	480	I				
	75	45	120	190	I				
	95	75	170	210	I				I
	135	145	280	340	I		I	I	
	100	135	235	245	I	I	I	I	I
	140	125	265	335	I		I	I	
	145	145	290	375	I		I		
	120	80	200	245	I	I	I	I	I
	110	115	225	265	I	I	I	I	I
	150	195	345	450	I				
	151	153	304	390	I				
	150	135	285	380	I		I		
	100	90	190	230	I	I	I	I	I
	115	85	200	230	I	I	I	I	I
	110	80	190	190	I	I	I	I	
	95	85	180	170	I		I	I	
	100	65	165	170	I	I			
	145	205	350	425	I				
	100	50	150	165	I	I			
	115	135	250	315	I	I	I	I	I
	125	140	265	310	I	I	I	I	I
	100	70	170	170	I	I			
	120	140	260	300	I	I	I	I	I
	125	125	250	305	I	I	I	I	I
	125	155	280	310	I	I		I	I
	120	140	260	315	I	I	I	I	I
	115	210	325	280	I	I			I
	120	145	265	325	I	I	I	I	I
	150	160	310	380	I				
	120	75	195	245	I	I		I	I
	120	110	230	260	I	I	I	I	I

128	143	271	326	I		I	I	
120	135	255	303	I	I	I	I	I
120	161	281	310	I	I			I
92	29	121	122	I				
116	107	223	219	I	I	I	I	I
75	22	97	98	I				
78	87	165	202	I		I		I
40	20	60	83	I				
98	82	180	163	I		I	I	
110	101	211	221	I	I	I	I	I
79	18	97	93	I				
240	460	700	760	II				
200	350	550	620	II	II	II	II	II
190	350	540	720	II	II	II	II	II
200	380	580	610	II	II	II	II	II
220	400	620	720	II	II	II	II	II
190	510	700	735	II	II			II
240	200	440	600	II			II	II
200	262	462	620	II	II	II	II	II
170	285	455	595	II	II	II	II	II
175	230	405	500	II	II			
190	320	510	530	II	II	II	II	
160	230	390	490	II				
245	470	715	875	II				III
160	180	340	425	II				
260	480	740	875	II	III			III
185	295	480	550	II	II	II	II	II
230	325	555	730	II		II	II	II
220	325	545	615	II	II	II	II	II
165	195	360	465	II				
205	340	545	695	II	II	II	II	II
190	335	525	670	II	II	II	II	II
230	345	575	720	II		II	II	II
285	535	820	890	II				III
225	460	685	955	II	II			IV
255	470	725	845	II	III			III
205	275	480	700	II	II	II	II	II
275	525	800	790	II	III			III
155	245	400	510	II				
185	245	430	500	II	II			
240	510	750	725	II				II
150	225	365	440	II				
215	395	610	650	II	II	II	II	II
175	275	450	595	II	II	II	II	II
155	250	405	585	II				II

235	535	770	770	II				
160	265	425	530	II		II		
185	370	555	875	II	II	II	II	III
185	250	435	590	II	II		II	II
175	320	495	580	II	II	II	II	II
200	425	625	675	II	II	II	II	II
200	370	570	605	II	II	II	II	II
225	340	565	775	II	II	II	II	
180	325	505	575	II	II	II	II	II
165	290	455	575	II		II	II	II
185	280	465	600	II	II	II	II	II
170	230	400	500	II	II			
229	548	777	869	II				III
184	344	529	687	II	II	II	II	II
254	537	791	932	II	III			III
150	190	340	427	II				
261	508	769	884	II	III			III
197	308	505	644	II	II	II	II	II
264	507	771	920	II	III			III
168	257	425	701	II		II		II
175	354	529	585	II	II	II	II	II
164	246	410	507	II				
225	444	670	756	II	II	II	II	
163	241	404	496	II				
208	484	692	705	II	II			II
142	210	352	409	II				
167	276	443	505	II		II	II	
250	485	735	780	III	III			
255	455	710	726	III	III			II
250	575	825	690	III	III	III	III	II
250	575	825	765	III	III	III	III	
250	685	935	865	III	III	IV		III
280	550	830	920	III	III		III	III
270	635	905	945	III	III	III	III	
275	625	900	1040	III	III	III	III	IV
250	515	765	765	III	III			
265	625	890	815	III	III	III	III	III
230	670	900	850	III			III	III
285	645	930	930	III				III
250	575	825	790	III	III	III	III	III
285	665	950	870	III				III
285	684	969	1106	III		IV		
270	679	949	1026	III	III	IV		IV
282	635	917	896	III		III		III
227	486	713	783	III				

	280	611	891	958	III	III	III	III	IV
	278	571	849	791	III	III	III	III	III
	295	621	916	958	III	IV	III	III	IV
	243	484	728	785	III				III
	314	826	1140	940	IV	IV			III
	360	550	910	960	IV			III	IV
	330	620	950	840	IV	IV	III		III
	340	690	1030	1165	IV		IV	IV	
	280	690	970	1045	IV	III	IV		IV
	330	660	990	1050	IV	IV		IV	IV
	335	630	965	1000	IV		III		IV
	270	705	975	1035	IV	III	IV	IV	IV
	325	655	980	1060	IV	IV		IV	IV
	315	675	990	1160	IV	IV	IV	IV	
	355	695	1050	890	IV		IV	IV	III
	310	685	995	1140	IV	IV	IV	IV	
	345	640	985	1015	IV		III	IV	IV
	325	825	1150	975	IV	IV			IV
	290	710	1000	965	IV		IV	IV	IV
	275	665	940	875	IV	III			III
	350	925	1275	975	IV				IV
	320	740	1060	935	IV	IV	IV	IV	III
	310	740	1050	975	IV	IV	IV	IV	IV
	300	700	1000	960	IV	IV	IV	IV	IV
	270	680	950	885	IV	III	IV		III
	310	665	975	950	IV	IV		IV	IV
	290	850	1140	985	IV				IV
	270	680	950	910	IV	III	IV		III
	322	813	1135	1148	IV	IV			
	346	725	1071	1190	IV		IV	IV	
	305	665	969	1082	IV	IV			
	333	743	1076	1179	IV		IV		
	285	752	1037	1066	IV		IV	IV	
	320	706	1026	1099	IV	IV	IV	IV	
	298	738	1036	946	IV	IV	IV	IV	
	306	722	1028	985	IV	IV	IV	IV	IV
	322	763	1085	1038	IV	IV			IV
	287	755	1041	937	IV			IV	III
	333	829	1162	1052	IV				IV
	332	716	1048	1050	IV	IV	IV	IV	IV
	294	798	1092	979	IV	IV			IV
CAT	131	126	257	305	I				
	82	41	123	137	I	I	I	I	I
	80	0	83	82	I	I			I

35	0	35	38	I				
114	63	176		I				
70	18	88	104	I		I	I	I
189	385	573	876	II	II	II	II	II
186	283	469	759	II	II		II	II
205	376	582	885	II	II	II	II	
177	264	441	668	II	II			
201	406	607	864	II	II	II	II	II
222	441	663	878	II				II
162	281	443	717	II				
217	419	636	879	II				
169	281	451	746	II				II
206	401	607	886	II	II	II	II	
156	289	445	684	II		II		
214	400	614	408	II	II	II	II	
222	548	770	990	II				III
218	506	724	826	II				II
177	314	492	744	II	II	II	II	II
174	371	546	860	II		II	II	II
266	622	888	977	III		III	III	
263	590	853	991	III		III	III	III
237	647	884	987	III		III	III	III
246	688	934	974	III	III			
249	640	890	1008	III	III	III	III	III
229	478	707	1018	III				
255	576	831	980	III	III		III	III
240	590	831	1001	III	III	III	III	III
269	712	982	1046	III				
256	754	1010	1137	III	III			
251	656	907	985	III	III	III	III	III
239	495	733	986	III				III
255	586	841	967	III	III	III	III	
346	1318	1664	1169	IV				
281	774	1055	1070	IV				IV
371	1394	1765	1109	IV				IV
269	1013	1282	1055	IV		IV	IV	IV
282	740	1022	1005	IV				III
309	944	1254	1076	IV	IV	IV	IV	IV
321	985	1306	1138	IV	IV	IV	IV	
274	731	1005	1045	IV				
354	1333	1687	1159	IV				
300	943	1243	1057	IV	IV	IV	IV	IV
384	1151	1535	1142	IV				
328	968	1296	1110	IV		IV	IV	IV
297	944	1241	1054	IV	IV	IV	IV	

	313	1061	1373	1087	IV	IV	IV	IV	IV
	297	989	1285	1067	IV	IV	IV	IV	IV
	346	1100	1445	1110	IV				IV
	298	812	1110	1066	IV	IV	IV	IV	IV
	322	1615	1937	1109	IV	IV			IV
	295	818	1113	1054	IV	IV	IV	IV	
	312	1319	1631	1112	IV	IV			IV
	286	752	1038	1119	IV				
	321	1003	1324	1117	IV	IV	IV	IV	
	287	745	1032	1007	IV				III
	308	806	1115	1036	IV	IV		IV	
	322	888	1210	1057	IV	IV	IV	IV	IV
	275	698	974	1016	IV				
	326	872	1198	1101	IV		IV	IV	IV
	292	817	1108	1116	IV	IV	IV		
PI9	90	60	150	145	I				
	125	130	255	305	I		I		
	136	164	300	345	I				
	105	85	190	215	I	I	I	I	I
	123	163	288	305	I				
	100	90	190	200	I	I	I	I	I
	120	115	235	250	I	I	I	I	I
	95	85	180	175	I		I		
	120	125	245	285	I	I	I	I	I
	132	175	307	390	I				
	100	85	185	175	I	I	I		
	105	85	190	190	I	I	I	I	I
	90	55	145	140	I				
	115	125	240	245	I	I	I	I	I
	110	100	210	205	I	I	I	I	I
	110	140	250	255	I	I		I	I
	215	350	565	1000	II				
	165	250	415	605	II	II			
	160	260	420	655	II	II	II		II
	175	295	470	695	II	II	II	II	II
	160	370	530	800	II	II			II
	160	305	465	785	II	II	II	II	II
	190	330	520	875	II		II	II	
	185	315	500	910	II	II	II	II	
	145	205	350	455	II				
	180	345	525	795	II	II			II
	140	187	327	400	II				
	190	310	500	880	II		II	II	
	185	310	495	845	II	II	II	II	II

170	390	560	950	II	II			
175	270	445	670	II	II	II	II	II
175	290	465	750	II	II	II	II	II
197	365	562	1030	II				
175	275	450	705	II	II	II	II	II
145	185	330	385	II				
190	340	530	795	II				II
160	260	420	665	II	II	II		II
180	290	470	805	II	II	II	II	II
132	195	327	355	II				
180	275	455	770	II	II	II	II	II
190	260	450	650	II		II	II	
190	365	555	845	II				II
165	285	450	650	II	II	II	II	
180	305	485	830	II	II	II	II	II
220	430	650	1010	III				
225	430	655	1025	III	III			
220	480	700	1080	III			III	
220	435	655	1100	III				
230	475	705	1040	III	III			III
230	460	690	1075	III	III	III	III	
225	375	600	1045	III	III			III
235	465	700	1042	III	III	III	III	III
220	450	670	1055	III		III	III	III
235	470	705	1060	III	III			III
236	526	762	1060	III				III
235	455	690	1010	III	III	III	III	
230	450	680	1030	III	III	III	III	
235	465	700	1070	III	III	III	III	
320	745	1065	1405	IV		IV	IV	IV
270	570	840	1215	IV	IV			
300	730	1030	1315	IV	IV	IV	IV	IV
315	885	1200	1410	IV	IV			IV
255	635	890	1250	IV				IV
315	855	1170	1430	IV	IV			IV
280	710	990	1240	IV	IV	IV	IV	
320	755	1075	1405	IV		IV	IV	IV
265	775	1040	1345	IV		IV	IV	IV
315	965	1280	1490	IV	IV			
245	505	750	1105	IV				
310	775	1085	1485	IV	IV	IV	IV	
285	920	1205	1500	IV	IV			
260	645	905	1260	IV				IV
330	820	1150	1480	IV		IV		
265	665	930	1265	IV		IV		IV

		240	480	720	1035	IV				III
		325	880	1205	1485	IV				
		300	700	1000	1325	IV	IV	IV	IV	IV
		240	565	805	1120	IV				
		290	685	975	1240	IV	IV	IV	IV	
		325	770	1095	1475	IV		IV	IV	
		335	920	1255	1465	IV				
		285	785	1070	1350	IV	IV	IV	IV	IV
		255	513	768	1090	IV				
		330	910	1240	1470	IV				
		280	725	1005	1275	IV	IV	IV	IV	IV
		245	495	740	1095	IV				
		315	760	1075	1400	IV	IV	IV	IV	IV
		270	630	900	1240	IV	IV			
		305	715	1020	1455	IV	IV	IV	IV	IV
		260	680	940	1210	IV		IV	IV	
		247	476	723	1205	IV				
		330	805	1135	1485	IV		IV	IV	
		285	715	1000	1270	IV	IV	IV	IV	IV
		305	815	1120	1310	IV	IV	IV	IV	IV
		280	660	940	1270	IV	IV		IV	IV
		340	875	1215	1500	IV				
		280	670	950	1250	IV	IV	IV	IV	IV
		330	925	1255	1470	IV				
		270	775	1045	1270	IV	IV	IV	IV	IV
		320	865	1185	1455	IV				IV
		270	590	860	1275	IV	IV			IV
		325	950	1275	1490	IV				
		280	670	950	1345	IV	IV	IV	IV	IV
		260	485	745	1130	IV				
		310	830	1140	1430	IV	IV		IV	IV
		275	690	965	1225	IV	IV	IV	IV	
		335	910	1245	1520	IV				
		275	755	1030	1305	IV	IV	IV	IV	IV
75	DL	120	149	269	353	I	I	I	I	I
		121	65	186	216	I	I	I	I	I
		80	0	80	110	I				
		108	86	194	226	I	I	I	I	I
		65	10	75	70	I		I		
		118	107	224	284	I	I	I	I	I
		104	0	104	114	I	I		I	I
		122	87	209	315	I	I	I	I	I
		125	104	229	263	I	I	I	I	I
		75	15	90	112	I		I		I

142	133	275	324	I	I	I	I	I
117	92	208	269	I	I	I	I	I
124	123	247	302	I	I	I	I	I
90	10	100	110	I	I	I	I	
147	153	300	410	I		I		
105	50	155	190	I	I	I	I	I
105	56	161	199	I	I	I	I	I
141	158	299	469	I	I			
153	155	308	388	I				
136	158	295	380	I	I		I	I
109	61	170	163	I	I	I	I	I
143	147	291	340	I		I	I	I
219	340	559	736	II	II	II	II	II
170	218	388	553	II		II	II	II
221	432	653	922	II	II	II	II	
207	337	545	730	II	II	II	II	II
162	204	366	494	II		II		
250	446	696	926	II				
216	388	603	785	II	II	II	II	II
188	271	458	628	II	II	II	II	II
147	185	332	398	II				
237	380	618	858	II	II	II	II	II
213	322	535	712	II	II	II	II	II
174	195	368	496	II	II			
226	378	604	876	II	II	II	II	II
200	328	528	732	II	II	II	II	II
235	364	599	900	II	II	II	II	II
212	315	527	723	II	II	II	II	II
185	191	376	530	II	II		II	II
255	449	704	961	II				
227	400	627	842	II	II	II	II	II
192	304	496	663	II	II	II	II	II
158	198	356	421	II				
230	401	631	906	II	II	II	II	II
237	393	630	771	II	II	II	II	II
225	458	683	966	II	II			
213	305	518	859	II	II	II	II	II
180	266	446	686	II	II	II	II	II
170	203	373	463	II				
221	381	602	887	II	II	II	II	II
210	352	562	740	II	II	II	II	II
179	230	409	553	II	II	II	II	II
240	439	679	927	II				
203	313	516	797	II	II	II	II	II
181	266	447	592	II	II	II	II	II

238	413	651	937	II		II	II	
202	346	548	807	II	II	II	II	II
183	239	422	603	II	II	II	II	II
159	177	336	400	II				
245	433	678	831	II				II
173	292	465	620	II	II	II	II	II
233	391	624	909	II	II	II	II	II
218	398	616	793	II	II	II	II	II
198	269	467	582	II	II	II	II	II
165	174	339	414	II				
254	483	737	956	II				
221	383	604	808	II	II	II	II	II
186	237	423	582	II	II	II	II	II
261	462	723	920	II				II
192	312	503	696	II	II	II	II	II
190	278	468	618	II	II	II	II	II
191	302	493	760	II	II	II	II	II
197	273	470	540	II	II	II	II	II
298	573	871	1042	III		III		III
279	506	786	1031	III	III		III	III
271	545	816	1055	III	III	III	III	III
271	540	811	980	III	III	III	III	III
293	573	866	1064	III	III	III	III	III
297	515	813	1008	III		III	III	III
294	546	841	1112	III	III	III	III	IV
268	508	776	1039	III	III	III		III
265	545	810	980	III		III	III	III
271	537	808	1052	III	III	III	III	III
262	498	760	1005	III				III
280	564	844	977	III	III	III	III	
290	523	812	1060	III	III	III	III	III
286	582	867	1066	III	III			
281	519	801	1011	III	III	III	III	III
339	855	1193	1231	IV	IV			
341	658	999	1167	IV		IV	IV	IV
305	656	961	1086	IV	IV			IV
323	815	1137	1156	IV	IV	IV	IV	IV
312	718	1030	1079	IV	IV	IV	IV	
347	836	1184	1187	IV				
321	758	1078	1080	IV	IV	IV	IV	IV
326	781	1107	1124	IV	IV	IV	IV	IV
326	702	1028	1080	IV	IV	IV	IV	IV
302	721	1023	1118	IV		IV	IV	IV
321	753	1074	1112	IV	IV	IV	IV	IV
320	797	1117	1162	IV	IV	IV	IV	IV

	319	658	976	1076	IV	IV	IV	IV	
	304	642	946	1077	IV				
	298	814	1112	1114	IV		IV	IV	IV
	336	805	1141	1199	IV	IV	IV	IV	
	328	627	955	1092	IV	IV			IV
	341	845	1186	1131	IV				IV
	325	747	1072	1082	IV	IV	IV	IV	IV
	311	750	1061	1185	IV	IV	IV	IV	IV
	326	710	1036	1112	IV	IV	IV	IV	IV
DP	94	46	140	166	I	I	I	I	I
	124	67	191	212	I	I	I	I	I
	129	106	235	237	I	I	I	I	I
	81	36	117	100	I		I	I	
	92	0	92	95	I	I			
	107	34	142	160	I	I	I	I	I
	90	29	119	113	I	I	I	I	I
	109	87	196	248	I	I	I	I	I
	137	140	277	342	I				
	138	155	293	316	I				
	81	48	128	152	I		I	I	I
	102	96	198	161	I	I	I	I	I
	84	26	110	137	I	I			I
	96	39	136	153	I	I	I	I	I
	109	51	160	160	I	I	I	I	I
	105	61	166	158	I	I	I	I	I
	222	394	615	889	II	II	II	II	
	156	181	337	440	II				
	222	405	627	874	II	II	II	II	
	208	324	532	702	II	II	II	II	II
	153	200	353	465	II				
	251	445	696	891	II				
	178	285	462	692	II	II	II	II	II
	161	193	354	461	II				
	251	435	686	813	II				II
	196	258	454	632	II	II	II	II	II
	154	157	311	370	II				
	219	410	629	961	II	II		II	III
	236	372	608	785	II	II	II	II	II
	193	287	479	591	II	II	II	II	II
	217	301	518	752	II	II	II	II	II
	174	222	396	492	II	II	II	II	II
	199	362	560	719	II	II	II	II	II
	215	327	542	723	II	II	II	II	II
	164	210	374	434	II		II		

261	418	678	850	II				II
213	310	524	665	II	II	II	II	II
237	373	610	728	II	II	II	II	II
190	253	443	516	II	II	II	II	II
226	335	561	701	II	II	II	II	II
169	199	369	444	II	II			
257	432	689	861	II				
236	319	555	663	II	II	II	II	II
218	309	528	690	II	II	II	II	II
229	410	640	850	II	II		II	II
211	293	504	651	II	II	II	II	II
151	177	328	425	II				
247	397	644	769	II	II	II	II	II
220	369	589	826	II	II	II	II	II
224	299	523	737	II	II	II	II	II
218	370	588	750	II	II	II	II	II
172	258	431	535	II	II	II	II	II
209	403	612	770	II	II	II	II	II
166	204	370	551	II				II
223	355	578	742	II	II	II	II	II
175	268	443	558	II	II	II	II	II
263	408	671	854	II		II		
202	325	527	661	II	II	II	II	II
166	222	388	445	II		II	II	
223	359	581	754	II	II	II	II	II
194	205	399	570	II	II		II	II
180	253	432	563	II	II	II	II	II
250	445	695	885	II	II			
212	283	496	665	II	II	II	II	II
253	386	638	734	II		II	II	II
189	268	456	563	II	II	II	II	II
276	470	746	852	II	III			II
203	324	528	620	II	II	II	II	II
241	339	580	772	II	II	II	II	II
179	272	451	517	II	II	II	II	II
247	399	646	811	II	II	II	II	II
194	276	470	550	II	II	II	II	II
262	385	647	903	II		II		
223	326	548	700	II	II	II	II	II
290	527	816	1019	III	III	III	III	
274	530	804	997	III	III	III	III	III
266	496	762	991	III	III			III
259	525	784	941	III		III	III	
253	568	821	952	III		III	III	III
280	606	886	1005	III	III		III	III

	272	548	821	960	III	III	III	III	III
	270	505	775	961	III	III			III
	289	511	800	943	III	III	III	III	III
	276	536	812	1000	III	III	III	III	III
	273	539	812	930	III	III	III	III	
	295	605	899	994	III		III		III
	264	547	811	936	III		III	III	
	296	532	828	1048	III		III	III	
	289	605	893	981	III	III	III		III
	273	500	772	948	III	III			III
	300	563	864	1018	III		III	III	
	284	647	931	999	III	III			III
	384	999	1383	1328	IV				
	345	906	1252	1281	IV	IV	IV	IV	IV
	342	988	1329	1200	IV	IV			IV
	384	916	1300	1335	IV		IV	IV	
	355	834	1189	1138	IV	IV	IV	IV	IV
	315	793	1108	1111	IV	IV	IV	IV	IV
	357	848	1205	1278	IV	IV	IV	IV	IV
	304	657	961	1016	IV				III
	348	869	1217	1151	IV	IV	IV	IV	IV
	332	919	1251	1132	IV	IV	IV	IV	IV
	289	760	1049	1026	IV	III	IV	IV	
	324	762	1086	1100	IV	IV	IV	IV	IV
	333	938	1271	1104	IV	IV	IV	IV	IV
	349	854	1202	1092	IV	IV	IV	IV	IV
	285	735	1020	1046	IV	III			
	334	808	1142	1100	IV	IV	IV	IV	IV
	325	773	1098	1085	IV	IV	IV	IV	IV
	312	657	969	999	IV				III
	394	830	1224	1270	IV		IV	IV	IV
	379	894	1273	1292	IV	IV	IV	IV	IV
	338	775	1150	1248	IV	IV	IV	IV	IV
	320	881	1201	1150	IV	IV	IV	IV	IV
	360	959	1200	1234	IV	IV		IV	IV
	315	689	1004	1116	IV	IV			IV
	369	940	1309	1210	IV	IV	IV		IV
	368	878	1246	1340	IV	IV	IV	IV	
	411	993	1404	1367	IV				
DW	105	89	194	179	I	I	I	I	I
	116	140	257	254	I	I	I	I	I
	84	58	143	138	I		I	I	I
	112	127	240	221	I	I	I	I	I
	79	39	118	108	I				

107	104	211	167	I	I	I	I	I
89	5	89	105	I	I			
133	179	312	250	I				I
77	19	96	114	I				
125	141	266	334	I	I	I	I	
99	99	198	205	I	I	I	I	I
129	171	300	284	I	I	I	I	I
130	177	307	316	I	I			
104	75	178	171	I	I	I	I	I
68	5	68	80	I				
128	177	305	273	I	I			I
92	40	131	148	I	I	I	I	I
125	165	290	281	I	I	I	I	I
99	51	150	149	I	I	I	I	I
105	134	239	263	I	I	I	I	I
83	5	83	109	I				
99	153	252	258	I	I	I	I	I
89	31	120	126	I	I			
132	205	337	303	I				I
92	73	165	176	I	I	I	I	I
130	125	255	290	I	I	I	I	I
105	45	150	170	I	I	I	I	I
107	96	203	230	I	I	I	I	I
128	167	295	321	I	I	I	I	
126	162	288	297	I	I	I	I	I
92	55	147	154	I	I	I	I	I
135	175	310	315	I				I
95	80	175	175	I	I	I	I	I
130	155	285	305	I	I	I	I	I
130	115	245	285	I	I	I	I	I
95	80	175	165	I	I	I	I	I
120	120	240	240	I	I	I	I	I
120	135	255	265	I	I	I	I	I
95	108	203	226	I	I	I	I	I
125	155	280	285	I	I	I	I	I
140	190	330	365	I				
120	115	235	225	I	I	I	I	I
130	170	300	320	I	I	I	I	
73	36	109	117	I				
130	165	295	315	I	I	I	I	I
115	95	210	240	I	I	I	I	I
105	125	230	240	I	I	I	I	I
120	80	200	245	I	I	I	I	I
100	50	150	190	I	I	I	I	I
126	179	305	318	I	I			

251	563	813	1050	II				III
175	361	536	628	II	II	II	II	II
145	211	357	351	II				
281	650	931	1117	II	III	III	III	III
243	595	837	962	II				II
190	354	544	706	II	II	II	II	II
144	265	409	468	II		II	II	
273	637	910	1134	II				III
233	588	821	992	II	II			
206	416	622	743	II	II	II	II	II
206	415	621	677	II	II	II	II	II
147	234	381	404	II				
195	508	703	892	II	II	II	II	II
228	477	706	891	II	II	II	II	II
189	366	555	620	II	II	II	II	II
263	548	811	978	II		II		II
185	461	646	781	II	II	II	II	II
191	393	584	832	II	II	II	II	II
178	319	497	577	II	II	II	II	II
222	486	708	894	II	II	II	II	II
174	342	516	611	II	II	II	II	II
141	227	368	369	II				
256	584	840	1007	II				
190	356	546	787	II	II	II	II	II
162	292	454	535	II	II	II	II	II
171	279	451	552	II	II	II	II	II
226	590	816	997	II	II			
194	401	595	764	II	II	II	II	II
148	236	384	492	II	II			II
205	428	633	787	II	II	II	II	II
161	288	448	516	II	II	II	II	II
247	514	761	982	II		II	II	II
202	402	605	742	II	II	II	II	II
156	270	426	471	II	II	II	II	II
232	562	794	1015	II	II		II	
205	434	639	753	II	II	II	II	II
148	273	421	496	II	II	II	II	II
206	441	647	1030	II	II	II	II	
193	422	615	822	II	II	II	II	II
171	307	478	570	II	II	II	II	II
158	249	407	649	II	II	II	II	II
210	455	665	965	II	II	II	II	II
195	350	545	670	II	II	II	II	II
165	240	405	485	II	II		II	II
249	454	703	893	II		II	II	II

212	308	520	628	II	II	II	II	II
143	192	335	362	II				
231	507	738	845	II	II	II	II	II
168	285	453	558	II	II	II	II	II
226	446	672	754	II	II	II	II	II
185	245	430	525	II	II	II	II	II
237	403	640	877	II	II	II	II	II
175	300	475	530	II	II	II	II	II
192	314	506	650	II	II	II	II	II
145	260	405	430	II		II	II	
144	206	350	376	II				
145	240	385	450	II				
217	483	700	765	II	II	II	II	II
155	255	410	485	II	II	II	II	II
286	874	1160	1296	III	III	IV		IV
309	774	1083	1214	III	IV	III	III	IV
275	680	955	1077	III		III	III	III
305	853	1158	1247	III	III	IV		IV
292	802	1094	1157	III	III	III	III	
280	663	943	1012	III	III	III	III	
283	775	1058	1137	III	III	III	III	III
294	767	1061	1193	III	III	III	III	
283	651	934	1082	III	III	III	III	III
291	767	1058	1136	III	III	III	III	III
308	819	1128	1205	III		III	III	
291	779	1070	1112	III	III	III	III	III
277	751	1028	1088	III	III	III	III	III
279	613	892	1012	III	III			
284	823	1106	1200	III	III	III	III	
298	780	1077	1121	III	III	III	III	III
286	764	1051	1136	III	III	III	III	III
290	791	1081	1174	III	III	III	III	
308	776	1084	1042	III		III	III	III
295	695	990	1100	III	III	III	III	III
305	724	1029	1091	III	III	III	III	III
274	629	903	1068	III				III
298	872	1170	1067	III	III	IV		III
253	637	890	941	III				II
346	1187	1533	1320	IV	IV			
290	816	1106	1091	IV	III	III	III	III
308	948	1256	1313	IV		IV	IV	
329	874	1202	1198	IV	IV	IV	IV	
315	900	1215	1363	IV	IV	IV	IV	
330	845	1175	1280	IV	IV			IV
378	954	1332	1285	IV		IV	IV	IV

	331	1045	1377	1306	IV	IV	IV	IV	IV
	311	892	1203	1229	IV	IV	IV	IV	IV
	330	962	1292	1230	IV	IV	IV	IV	IV
	301	858	1159	1240	IV	III	IV		IV
	340	1064	1404	1290	IV	IV	IV	IV	IV
	334	1039	1373	1198	IV	IV	IV	IV	
	316	875	1191	1208	IV	IV	IV	IV	IV
	351	960	1311	1239	IV	IV	IV	IV	IV
	338	945	1283	1146	IV	IV	IV	IV	
	449	850	1299	1211	IV			IV	IV
	375	1101	1476	1143	IV				III
	363	1085	1448	1088	IV	IV			III
OTA	135	80	215	270	I	I	I	I	I
	125	45	170	175	I	I	I	I	
	175	145	320	390	I				
	155	80	235	240	I	I	I	I	I
	115	50	165	190	I		I	I	I
	105	40	145	140	I		I		
	120	30	150	180	I	I			I
	140	90	230	225	I	I	I	I	I
	150	125	275	335	I	I	I	I	I
	145	120	265	320	I	I	I	I	I
	135	40	175	245	I	I	I	I	I
	170	130	300	400	I		I		
	160	130	290	385	I	I	I	I	I
	120	45	165	180	I	I	I	I	I
	125	40	165	195	I	I	I	I	I
	260	565	825	1090	II	III	III	III	III
	265	455	720	1010	II	III	III	III	III
	225	325	550	750	II	II	II	II	II
	214	271	485	750	II	II	II	II	II
	240	400	640	870	II	II	II	II	II
	160	165	325	475	II	I			
	250	455	705	1000	II		III	III	III
	200	225	425	740	II	II	II	II	II
	205	265	470	780	II	II	II	II	II
	230	370	600	880	II	II	II	II	II
	180	215	395	510	II	II	II	II	II
	220	410	630	950	II	II	II	II	
	190	190	380	650	II	II		II	II
	260	430	690	995	II	III			III
	230	380	610	810	II	II	II	II	II
	160	195	355	410	II	I	II		
	230	280	510	690	II	II	II	II	II

255	405	660	955	II	III	II	II	
205	260	465	680	II	II	II	II	II
205	250	455	635	II	II	II	II	II
215	310	525	730	II	II	II	II	II
215	300	515	680	II	II	II	II	II
211	259	470	680	II	II	II	II	II
200	220	420	640	II	II	II	II	II
230	390	620	890	II	II	II	II	II
170	190	360	500	II				
230	370	600	890	II	II	II	II	II
170	210	380	540	II		II	II	II
240	355	595	885	II	II	II	II	II
240	325	565	795	II	II	II	II	II
200	415	615	955	II	II	II	II	
200	175	375	500	II	II			
170	190	360	415	II				
225	325	550	685	II	II	II	II	II
190	180	370	540	II	II			II
250	350	600	705	II		II	II	II
210	315	525	640	II	II	II	II	II
205	245	450	580	II	II	II	II	II
240	390	630	910	II	II	II	II	II
220	355	575	715	II	II	II	II	II
176	214	390	500	II		II	II	
240	345	585	810	II	II	II	II	II
225	235	460	630	II	II	II	II	II
200	330	530	660	II	II	II	II	II
165	240	405	520	II		II	II	II
285	525	810	1145	II	III	III	III	III
210	365	575	870	II	II	II	II	II
210	195	405	505	II	II	II	II	
220	300	520	670	II	II	II	II	II
250	480	730	935	II		III	III	II
230	385	615	1035	II	II	II	II	III
275	430	705	1130	II	III		III	III
270	550	820	1020	III	III	III	III	III
300	705	1005	1135	III	III			III
245	475	720	1020	III	II	III	III	III
325	695	1020	1100	III	IV	III		III
275	515	790	1050	III	III	III	III	III
300	580	880	1065	III	III	III	III	III
285	445	730	895	III	III	III	III	II
240	440	680	980	III	II			
305	790	1095	1125	III		IV	IV	III
285	695	980	1175	III	III	III	III	III

	245	500	745	1115	III	II	III	III	III
	265	685	950	1100	III	III	III	III	III
	265	530	795	950	III	III	III	III	
	265	535	800	975	III	III	III	III	
	265	435	700	795	III	III			II
	265	430	695	940	III	III			II
	320	645	965	1280	III	IV	III	III	IV
	300	595	895	1245	III	III	III	III	IV
	295	580	875	1265	III	III	III	III	IV
	385	1155	1540	1420	IV	IV	IV	IV	IV
	370	1010	1380	1370	IV	IV	IV	IV	IV
	315	1025	1340	1185	IV	IV	IV	IV	III
	325	1005	1330	1265	IV	IV	IV	IV	IV
	340	1025	1365	1240	IV	IV	IV	IV	IV
	360	730	1090	1240	IV	IV	IV	IV	IV
	335	705	1040	1150	IV	IV			III
	325	840	1165	1300	IV	IV	IV	IV	IV
	295	780	1075	1200	IV	III	IV	IV	
	345	815	1160	1350	IV	IV	IV	IV	IV
	325	835	1160	1330	IV	IV	IV	IV	IV
	325	645	970	1160	IV	IV	III	III	III
	315	690	1005	1190	IV	IV	III		III
	415	1365	1780	1660	IV				
	350	970	1320	1325	IV	IV	IV	IV	IV
	355	925	1280	1330	IV	IV	IV	IV	IV
	330	940	1270	1370	IV	IV	IV	IV	IV
	415	1200	1615	1500	IV				
	315	960	1275	1345	IV	IV	IV	IV	IV
	320	815	1135	1250	IV	IV	IV	IV	IV
	390	1350	1740	1560	IV	IV			
	350	870	1220	1295	IV	IV	IV	IV	IV
	430	1480	1910	1580	IV				
	380	820	1200	1240	IV	IV	IV	IV	IV
	395	790	1185	1400	IV		IV	IV	IV
	305	975	1280	1310	IV		IV	IV	IV
	345	985	1330	1380	IV	IV	IV	IV	IV
	360	920	1280	1405	IV	IV	IV	IV	IV
	310	690	1000	1335	IV		III		IV
	315	745	1060	1390	IV	IV	IV	IV	IV
	335	765	1100	1300	IV	IV	IV	IV	IV
	365	825	1190	1310	IV	IV	IV	IV	IV
	330	715	1045	1400	IV	IV	IV	IV	IV
TU	130	210	340	360	I	I			I
	130	210	340	480	I	I			

75	45	120	190	I				I
95	75	170	210	I	I	I	I	I
135	145	280	340	I	I	I	I	I
100	135	235	245	I	I	I	I	I
140	125	265	335	I	I	I	I	I
145	145	290	375	I		I	I	I
120	80	200	245	I	I	I	I	I
110	115	225	265	I	I	I	I	I
150	195	345	450	I				
151	153	304	390	I		I	I	
150	135	285	380	I		I	I	
100	90	190	230	I	I	I	I	I
115	85	200	230	I	I	I	I	I
110	80	190	190	I	I	I	I	I
95	85	180	170	I	I	I	I	I
100	65	165	170	I	I	I	I	I
145	205	350	425	I				
100	50	150	165	I	I			
115	135	250	315	I	I	I	I	I
125	140	265	310	I	I	I	I	I
100	70	170	170	I	I	I	I	I
120	140	260	300	I	I	I	I	I
125	125	250	305	I	I	I	I	I
125	155	280	310	I	I	I	I	I
120	140	260	315	I	I	I	I	I
115	210	325	280	I	I			I
120	145	265	325	I	I	I	I	I
150	160	310	380	I		I		
120	75	195	245	I	I	I	I	I
120	110	230	260	I	I	I	I	I
128	143	271	326	I	I	I	I	I
120	135	255	303	I	I	I	I	I
120	161	281	310	I	I		I	I
92	29	121	122	I				
116	107	223	219	I	I	I	I	I
75	22	97	98	I				
78	87	165	202	I		I	I	I
40	20	60	83	I				
98	82	180	163	I	I	I	I	
110	101	211	221	I	I	I	I	I
79	18	97	93	I				
240	460	700	760	II	II	II	II	II
200	350	550	620	II	II	II	II	II
190	350	540	720	II	II	II	II	II
200	380	580	610	II	II	II	II	II

220	400	620	720	II	II	II	II	II
190	510	700	735	II	II	III	II	II
240	200	440	600	II	II		II	II
200	262	462	620	II	II	II	II	II
170	285	455	595	II	II	II	II	II
175	230	405	500	II	II	II	II	II
190	320	510	530	II	II	II	II	II
160	230	390	490	II		II		
245	470	715	875	II		II	II	III
160	180	340	425	II				
260	480	740	875	II	III	II		III
185	295	480	550	II	II	II	II	II
230	325	555	730	II	II	II	II	II
220	325	545	615	II	II	II	II	II
165	195	360	465	II	II			
205	340	545	695	II	II	II	II	II
190	335	525	670	II	II	II	II	II
230	345	575	720	II	II	II	II	II
285	535	820	890	II		III	III	III
225	460	685	955	II	II	II	II	
255	470	725	845	II	III	II	II	
205	275	480	700	II	II	II	II	II
275	525	800	790	II	III	III	III	
155	245	400	510	II		II		II
185	245	430	500	II	II	II	II	II
240	510	750	725	II	II	III	III	II
150	225	365	440	II				
215	395	610	650	II	II	II	II	II
175	275	450	595	II	II	II	II	II
155	250	405	585	II		II	II	II
235	535	770	770	II	II	III	III	
160	265	425	530	II		II	II	II
185	370	555	875	II	II	II	II	III
185	250	435	590	II	II	II	II	II
175	320	495	580	II	II	II	II	II
200	425	625	675	II	II	II	II	II
200	370	570	605	II	II	II	II	II
225	340	565	775	II	II	II	II	
180	325	505	575	II	II	II	II	II
165	290	455	575	II	II	II	II	II
185	280	465	600	II	II	II	II	II
170	230	400	500	II	II	II		II
229	548	777	869	II	II	III	III	
184	344	529	687	II	II	II	II	II
254	537	791	932	II	III	III	III	

150	190	340	427	II				
261	508	769	884	II	III	III	III	III
197	308	505	644	II	II	II	II	II
264	507	771	920	II	III		III	III
168	257	425	701	II	II	II	II	II
175	354	529	585	II	II	II	II	II
164	246	410	507	II	II	II	II	II
225	444	670	756	II	II	II	II	II
163	241	404	496	II	II	II	II	
208	484	692	705	II	II	II	II	II
142	210	352	409	II	I			
167	276	443	505	II	II	II	II	II
250	485	735	780	III	III	II		
255	455	710	726	III	III	II	II	II
250	575	825	690	III	III	III	III	II
250	575	825	765	III	III	III	III	
250	685	935	865	III	III	IV	III	
280	550	830	920	III	III	III	III	III
270	635	905	945	III	III	III	III	
275	625	900	1040	III	III	III	III	IV
250	515	765	765	III	III	III	III	
265	625	890	815	III	III	III	III	
230	670	900	850	III	II		III	
285	645	930	930	III		III	III	
250	575	825	790	III	III	III	III	
285	665	950	870	III				
285	684	969	1106	III		IV	IV	IV
270	679	949	1026	III	III	IV		IV
282	635	917	896	III	III	III	III	III
227	486	713	783	III	II		II	
280	611	891	958	III	III	III	III	
278	571	849	791	III	III	III	III	
295	621	916	958	III	IV	III	III	
243	484	728	785	III		II	II	
314	826	1140	940	IV	IV			
360	550	910	960	IV		III	III	
330	620	950	840	IV	IV	III		
340	690	1030	1165	IV	IV	IV	IV	
280	690	970	1045	IV	III	IV	IV	IV
330	660	990	1050	IV	IV		IV	IV
335	630	965	1000	IV	IV	III	IV	IV
270	705	975	1035	IV	III	IV	IV	IV
325	655	980	1060	IV	IV	III	IV	IV
315	675	990	1160	IV	IV	IV	IV	
355	695	1050	890	IV		IV	IV	III

	310	685	995	1140	IV	IV	IV	IV	IV
	345	640	985	1015	IV		III	IV	IV
	325	825	1150	975	IV	IV			
	290	710	1000	965	IV	IV	IV	IV	
	275	665	940	875	IV	III		III	III
	350	925	1275	975	IV				
	320	740	1060	935	IV	IV	IV	IV	
	310	740	1050	975	IV	IV	IV	IV	
	300	700	1000	960	IV	IV	IV	IV	
	270	680	950	885	IV	III	IV		III
	310	665	975	950	IV	IV		IV	
	290	850	1140	985	IV	IV			IV
	270	680	950	910	IV	III	IV		III
	322	813	1135	1148	IV	IV	IV	IV	
	346	725	1071	1190	IV		IV	IV	
	305	665	969	1082	IV	IV		IV	IV
	333	743	1076	1179	IV	IV	IV	IV	
	285	752	1037	1066	IV		IV	IV	IV
	320	706	1026	1099	IV	IV	IV	IV	IV
	298	738	1036	946	IV	IV	IV	IV	
	306	722	1028	985	IV	IV	IV	IV	IV
	322	763	1085	1038	IV	IV	IV	IV	IV
	287	755	1041	937	IV	IV	IV	IV	
	333	829	1162	1052	IV	IV			IV
	332	716	1048	1050	IV	IV	IV	IV	IV
	294	798	1092	979	IV	IV	IV	IV	
CAT	131	126	257	305	I				
	82	41	123	137	I	I	I	I	I
	80	0	83	82	I	I	I	I	I
	35	0	35	38	I		I		
	114	63	176		I	I	I	I	
	70	18	88	104	I	I	I	I	I
	189	385	573	876	II	II	II	II	II
	186	283	469	759	II	II	II	II	II
	205	376	582	885	II	II	II	II	II
	177	264	441	668	II	II			
	201	406	607	864	II	II	II	II	II
	222	441	663	878	II		II	II	II
	162	281	443	717	II		II		II
	217	419	636	879	II	II	II	II	II
	169	281	451	746	II	II	II	II	II
	206	401	607	886	II	II	II	II	
	156	289	445	684	II		II	II	II
	214	400	614	408	II	II	II	II	

222	548	770	990	II		III		III
218	506	724	826	II	II			II
177	314	492	744	II	II	II	II	II
174	371	546	860	II	II	II	II	II
266	622	888	977	III		III	III	III
263	590	853	991	III	III	III	III	III
237	647	884	987	III		III	III	III
246	688	934	974	III	III	III	III	
249	640	890	1008	III	III	III	III	III
229	478	707	1018	III				III
255	576	831	980	III	III	III	III	III
240	590	831	1001	III	III	III	III	III
269	712	982	1046	III				IV
256	754	1010	1137	III	III	IV		
251	656	907	985	III	III	III	III	III
239	495	733	986	III	III			III
255	586	841	967	III	III	III	III	
346	1318	1664	1169	IV	IV	IV		
281	774	1055	1070	IV		IV	IV	IV
371	1394	1765	1109	IV				IV
269	1013	1282	1055	IV		IV	IV	IV
282	740	1022	1005	IV	IV			III
309	944	1254	1076	IV	IV	IV	IV	IV
321	985	1306	1138	IV	IV	IV	IV	
274	731	1005	1045	IV				IV
354	1333	1687	1159	IV				
300	943	1243	1057	IV	IV	IV	IV	IV
384	1151	1535	1142	IV		IV	IV	
328	968	1296	1110	IV	IV	IV	IV	IV
297	944	1241	1054	IV	IV	IV	IV	IV
313	1061	1373	1087	IV	IV	IV	IV	IV
297	989	1285	1067	IV	IV	IV	IV	IV
346	1100	1445	1110	IV	IV	IV	IV	IV
298	812	1110	1066	IV	IV	IV	IV	IV
322	1615	1937	1109	IV	IV			IV
295	818	1113	1054	IV	IV	IV	IV	IV
312	1319	1631	1112	IV	IV		IV	IV
286	752	1038	1119	IV	IV	IV	IV	IV
321	1003	1324	1117	IV	IV	IV	IV	IV
287	745	1032	1007	IV	IV			III
308	806	1115	1036	IV	IV	IV	IV	
322	888	1210	1057	IV	IV	IV	IV	IV
275	698	974	1016	IV		III		III
326	872	1198	1101	IV	IV	IV	IV	IV

	292	817	1108	1116	IV	IV	IV	IV	IV
PI9	90	60	150	145	I				
	125	130	255	305	I	I	I	I	I
	136	164	300	345	I				
	105	85	190	215	I	I	I	I	I
	123	163	288	305	I	I	I	I	I
	100	90	190	200	I	I	I	I	I
	120	115	235	250	I	I	I	I	I
	95	85	180	175	I	I	I	I	I
	120	125	245	285	I	I	I	I	I
	132	175	307	390	I				
	100	85	185	175	I	I	I	I	I
	105	85	190	190	I	I	I	I	I
	90	55	145	140	I				
	115	125	240	245	I	I	I	I	I
	110	100	210	205	I	I	I	I	I
	110	140	250	255	I	I	I	I	I
	215	350	565	1000	II		II		
	165	250	415	605	II	II	II	II	II
	160	260	420	655	II	II	II	II	II
	175	295	470	695	II	II	II	II	II
	160	370	530	800	II	II		II	II
	160	305	465	785	II	II	II	II	II
	190	330	520	875	II	II	II	II	II
	185	315	500	910	II	II	II	II	
	145	205	350	455	II				
	180	345	525	795	II	II	II	II	II
	140	187	327	400	II				
	190	310	500	880	II	II	II	II	II
	185	310	495	845	II	II	II	II	II
	170	390	560	950	II	II			
	175	270	445	670	II	II	II	II	II
	175	290	465	750	II	II	II	II	II
	197	365	562	1030	II				III
	175	275	450	705	II	II	II	II	II
	145	185	330	385	II				
	190	340	530	795	II	II	II	II	II
	160	260	420	665	II	II	II	II	II
	180	290	470	805	II	II	II	II	II
	132	195	327	355	II				
	180	275	455	770	II	II	II	II	II
	190	260	450	650	II	II	II	II	II
	190	365	555	845	II	II			II
	165	285	450	650	II	II	II	II	II

180	305	485	830	II	II	II	II	II
220	430	650	1010	III	III	III		
225	430	655	1025	III	III	III	III	III
220	480	700	1080	III	III		III	
220	435	655	1100	III	III	III	III	
230	475	705	1040	III	III	III	III	III
230	460	690	1075	III	III	III	III	III
225	375	600	1045	III	III			III
235	465	700	1042	III	III	III	III	III
220	450	670	1055	III	III	III	III	III
235	470	705	1060	III	III	III	III	III
236	526	762	1060	III				III
235	455	690	1010	III	III	III	III	
230	450	680	1030	III	III	III	III	III
235	465	700	1070	III	III	III	III	III
320	745	1065	1405	IV	IV	IV	IV	IV
270	570	840	1215	IV	IV	IV	IV	IV
300	730	1030	1315	IV	IV	IV	IV	IV
315	885	1200	1410	IV	IV	IV	IV	IV
255	635	890	1250	IV		IV	IV	IV
315	855	1170	1430	IV	IV	IV	IV	IV
280	710	990	1240	IV	IV	IV	IV	IV
320	755	1075	1405	IV	IV	IV	IV	IV
265	775	1040	1345	IV	IV	IV	IV	IV
315	965	1280	1490	IV	IV			
245	505	750	1105	IV				
310	775	1085	1485	IV	IV	IV	IV	IV
285	920	1205	1500	IV	IV		IV	
260	645	905	1260	IV	IV	IV	IV	IV
330	820	1150	1480	IV		IV	IV	IV
265	665	930	1265	IV	IV	IV	IV	IV
240	480	720	1035	IV				III
325	880	1205	1485	IV	IV	IV	IV	IV
300	700	1000	1325	IV	IV	IV	IV	IV
240	565	805	1120	IV				
290	685	975	1240	IV	IV	IV	IV	IV
325	770	1095	1475	IV	IV	IV	IV	IV
335	920	1255	1465	IV				IV
285	785	1070	1350	IV	IV	IV	IV	IV
255	513	768	1090	IV				
330	910	1240	1470	IV				IV
280	725	1005	1275	IV	IV	IV	IV	IV
245	495	740	1095	IV				
315	760	1075	1400	IV	IV	IV	IV	IV
270	630	900	1240	IV	IV	IV	IV	IV

305	715	1020	1455	IV	IV	IV	IV	IV
260	680	940	1210	IV	IV	IV	IV	IV
247	476	723	1205	IV		III		
330	805	1135	1485	IV		IV	IV	IV
285	715	1000	1270	IV	IV	IV	IV	IV
305	815	1120	1310	IV	IV	IV	IV	IV
280	660	940	1270	IV	IV	IV	IV	IV
340	875	1215	1500	IV		IV		
280	670	950	1250	IV	IV	IV	IV	IV
330	925	1255	1470	IV				IV
270	775	1045	1270	IV	IV	IV	IV	IV
320	865	1185	1455	IV	IV	IV	IV	IV
270	590	860	1275	IV	IV	IV	IV	IV
325	950	1275	1490	IV	IV			
280	670	950	1345	IV	IV	IV	IV	IV
260	485	745	1130	IV	IV			
310	830	1140	1430	IV	IV	IV	IV	IV
275	690	965	1225	IV	IV	IV	IV	IV
335	910	1245	1520	IV				
275	755	1030	1305	IV	IV	IV	IV	IV

Parameters: pistil length (PL), ovary length (OL), style length (SL), and anther length (AL). Genotypes: DL (Don Luis), DP (Don Pablo), DW (Don Walter), TU (Tanganyika), OTA (OTA-S), CAT (Catalina), and PI9 (PI299920). Female developmental stages: I: Megaspore Mother Cell, II: Postmeiosis or EMMC, III: Immature embryo sac, and IV: Mature embryo sac.
