# Supplementary material for: A Precise Reproductive Calendar of Sexual and Apomictic Genotypes of Eragrostis curvula
Source: Plants (Basel). 2026 Mar 29;15(7):1050. doi: 10.3390/plants15071050 (PMC13074311; doi:10.3390/plants15071050)
Supplement: Supplementary file 1 [file plants-15-01050-s001.zip › supplementary material/Figure S6. AL Genotypes.pdf]

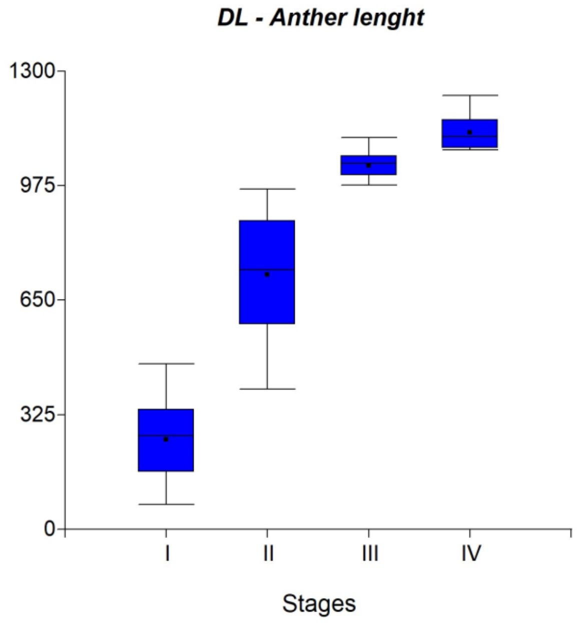
DL - Anther lenght
1300
975
650
325
0
I
II
III
IV
Stages

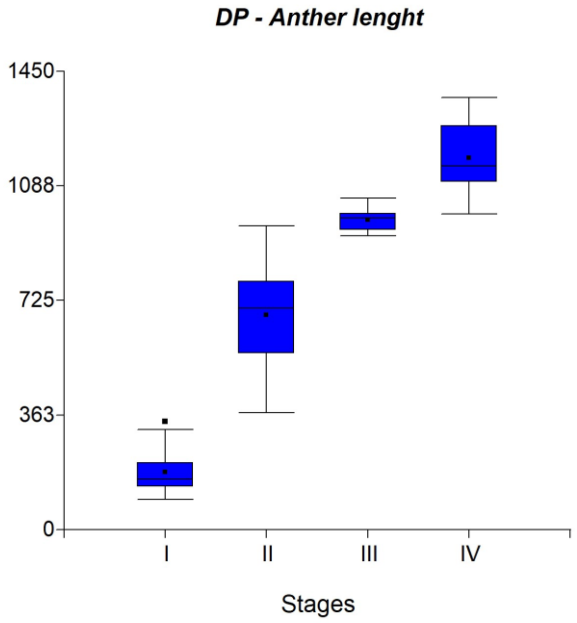
DP - Anther lenght
1450
1088
725
363
0
I
II
III
IV
Stages

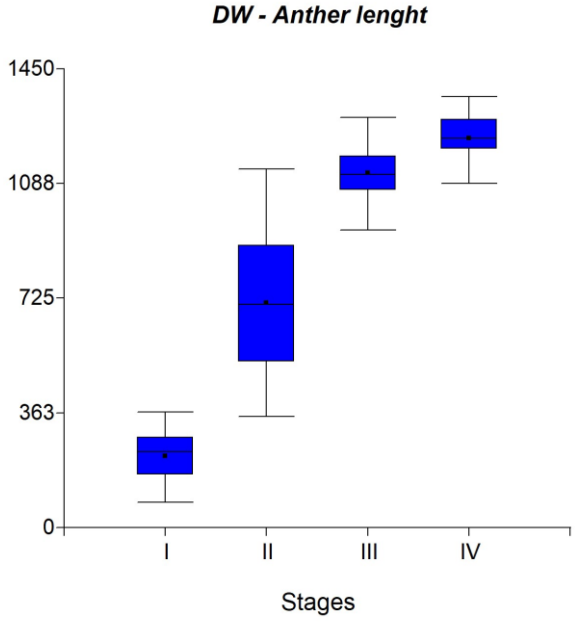
DW - Anther lenght
1450
1088
725
363
0
I
II
III
IV
Stages

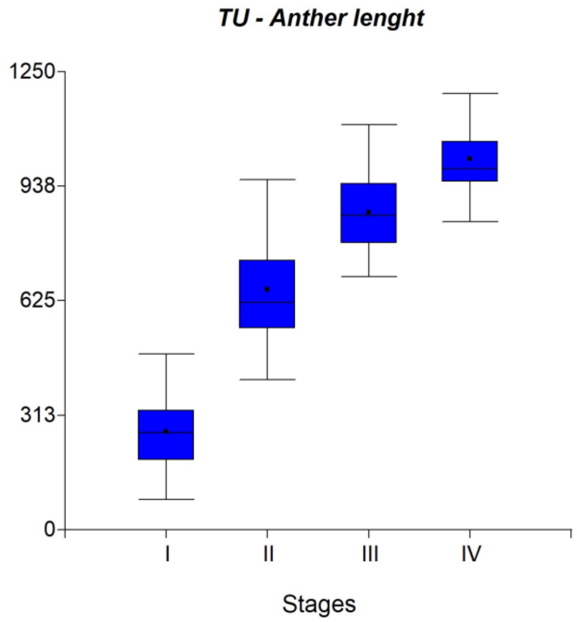
TU - Anther lenght
1250
938
625
313
0
I
II
III
IV
Stages

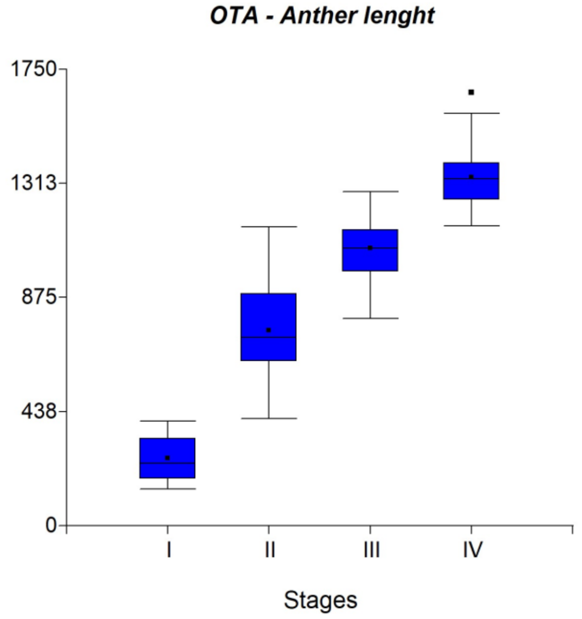
OTA - Anther lenght
1750
1313
875
438
0
I
II
III
IV
Stages

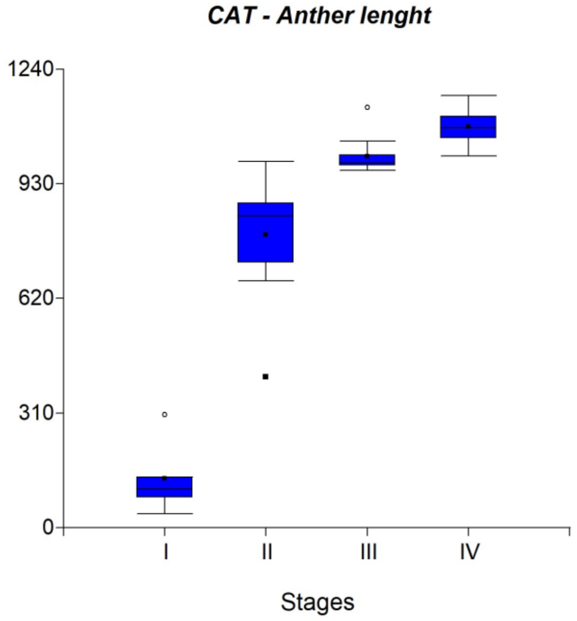
CAT - Anther lenght
1240
930
620
310
0
I
II
III
IV
Stages

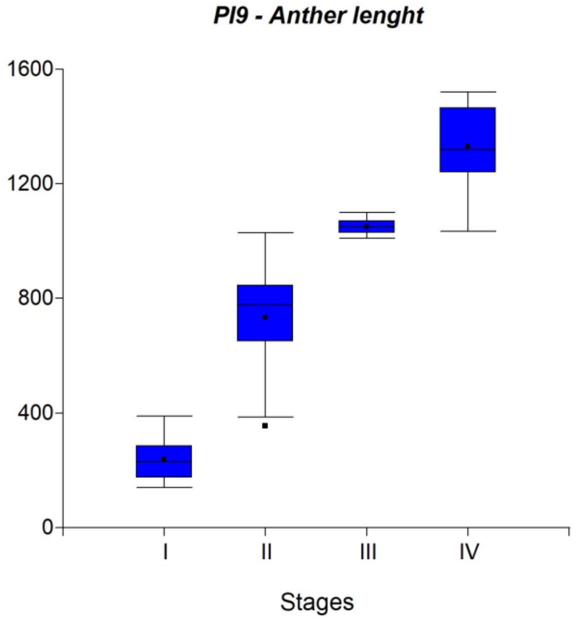
PI9 - Anther lenght
1600
1200
800
400
0
I
II
III
IV
Stages
