# Supplementary material for: A Precise Reproductive Calendar of Sexual and Apomictic Genotypes of Eragrostis curvula
Source: Plants (Basel). 2026 Mar 29;15(7):1050. doi: 10.3390/plants15071050 (PMC13074311; doi:10.3390/plants15071050)
Supplement: Supplementary file 1 [file plants-15-01050-s001.zip › supplementary material/Table S4. Confusion table generated from the 50th and 75th percentiles.pdf]

Table S4. Confusion table generated from the 50th and 75th percentiles to assess the classification accuracy of each parameter.

**Percentile 0.5**	**pred_I**	**pred_II**	**pred_III**	**pred_IV**	**pred_NONE_**
true_I	86	0	0	0	82
true_II	0	172	10	0	143
true_III	0	1	73	2	49
true_IV	0	0	9	108	98
true_NONE_	0	0	0	0	0
**Percentile 0.75**	pred_I	pred_II	pred_III	pred_IV	pred_NONE_
true_I	126	0	0	0	42
true_II	3	237	14	0	71
true_III	0	5	91	4	25
true_IV	0	0	10	156	49
true_NONE_	0	0	0	0	0
