# Supplementary material for: A Precise Reproductive Calendar of Sexual and Apomictic Genotypes of Eragrostis curvula
Source: Plants (Basel). 2026 Mar 29;15(7):1050. doi: 10.3390/plants15071050 (PMC13074311; doi:10.3390/plants15071050)
Supplement: Supplementary file 1 [file plants-15-01050-s001.zip › supplementary material/Table S2. Results of ANOVA comparing morphological parameters in each genotype and all genotypes in ea.pdf]

Table S2. Results of ANOVA comparing morphological parameters in each genotype and all genotypes in each parameter. F and *p-values* are reported for each analysis.

**Per Genotype**				**All genotypes**			
**Genotype**	**Parameter**	**F**	***p-value***	**Parameter**	**Female Stage**	**F**	***p-value***
DL	OL	313.29	<0.0001	OL	I	5.97	<0.0001
	SL	327.51	<0.0001		II	7.32	<0.0001
	PL	342.94	<0.0001		III	28.55	<0.0001
	AL	191.16	<0.0001		IV	17.76	<0.0001
DP	OL	264.95	<0.0001	SL	I	4.29	0.0050
	SL	439.84	<0.0001		II	4.72	0.0010
	PL	422.44	<0.0001		III	33.26	<0.0001
	AL	257.84	<0.0001		IV	18.19	<0.0001
DW	OL	382.03	<0.0001	PL	I	3.15	0.0060
	SL	471.04	<0.0001		II	2.89	0.0094
	PL	487.89	<0.0001		III	33.48	<0.0001
	AL	317.07	<0.0001		IV	18.09	<0.0001
TU	OL	356.18	<0.0001	AL	I	3.52	0.0027
	SL	397.05	<0.0001		II	2.45	0.0249
	PL	449.59	<0.0001		III	21.72	<0.0001
	AL	300.24	<0.0001		IV	54.6	<0.0001
OTA	OL	219.88	<0.0001				
	SL	193.06	<0.0001				
	PL	216.07	<0.0001				
	AL	200.37	<0.0001				
CAT	OL	165.55	<0.0001				
	SL	78.00	<0.0001				
	PL	94.91	<0.0001				
	AL	206.64	<0.0001				
PI9	OL	310.52	<0.0001				
	SL	219.07	<0.0001				
	PL	248.89	<0.0001				
	AL	327.37	<0.0001				

Genotypes: DL (Don Luis), DP (Don Pablo), DW (Don Walter), TU (Tanganyika), OTA (OTA-S), CAT (Catalina) and PI9 (PI299920). Parameters: pistil length (PL), ovary length (OL), style length (SL), and anther length (AL). Female developmental stages: I: Megaspore Mother Cell, II: Postmeiosis or EMMC, III: Immature embryo sac, and IV: Mature embryo sac.
