# Supplementary material for: A Precise Reproductive Calendar of Sexual and Apomictic Genotypes of Eragrostis curvula
Source: Plants (Basel). 2026 Mar 29;15(7):1050. doi: 10.3390/plants15071050 (PMC13074311; doi:10.3390/plants15071050)
Supplement: Supplementary file 1 [file plants-15-01050-s001.zip › supplementary material/Figure S4. OL Genotypes.pdf]

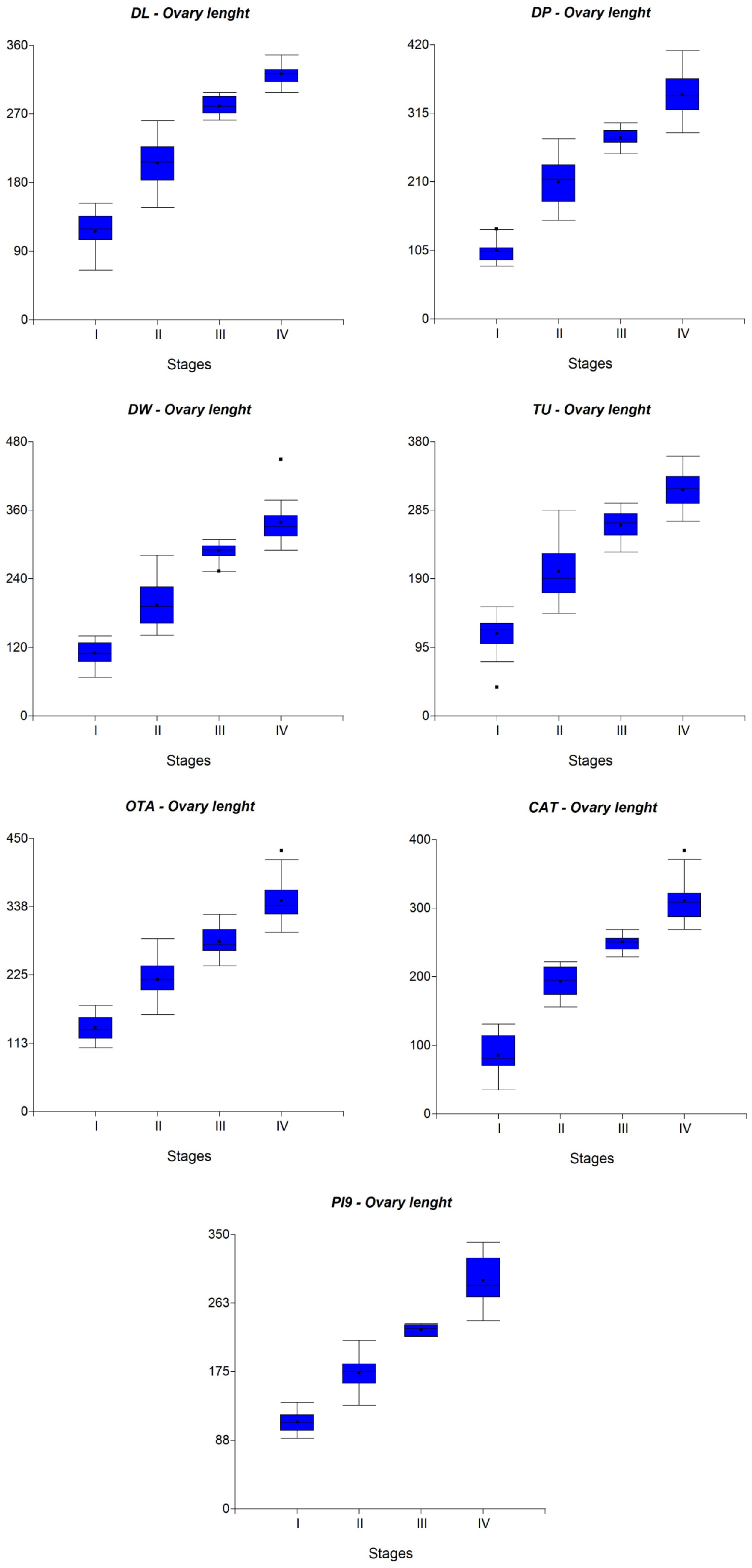
DL - Ovary lenght
360
270
180
90
0
I
II
III
IV
Stages
DP - Ovary lenght
420
315
210
105
0
I
II
III
IV
Stages
DW - Ovary lenght
480
360
240
120
0
I
II
III
IV
Stages
TU - Ovary lenght
380
285
190
95
0
I
II
III
IV
Stages
OTA - Ovary lenght
450
338
225
113
0
I
II
III
IV
Stages
CAT - Ovary lenght
400
300
200
100
0
I
II
III
IV
Stages
PI9 - Ovary lenght
350
263
175
88
0
I
II
III
IV
Stages
