# Supplementary figures and images for: A Precise Reproductive Calendar of Sexual and Apomictic Genotypes of Eragrostis curvula
Source: Plants (Basel). 2026 Mar 29;15(7):1050. doi: 10.3390/plants15071050 (PMC13074311; doi:10.3390/plants15071050)

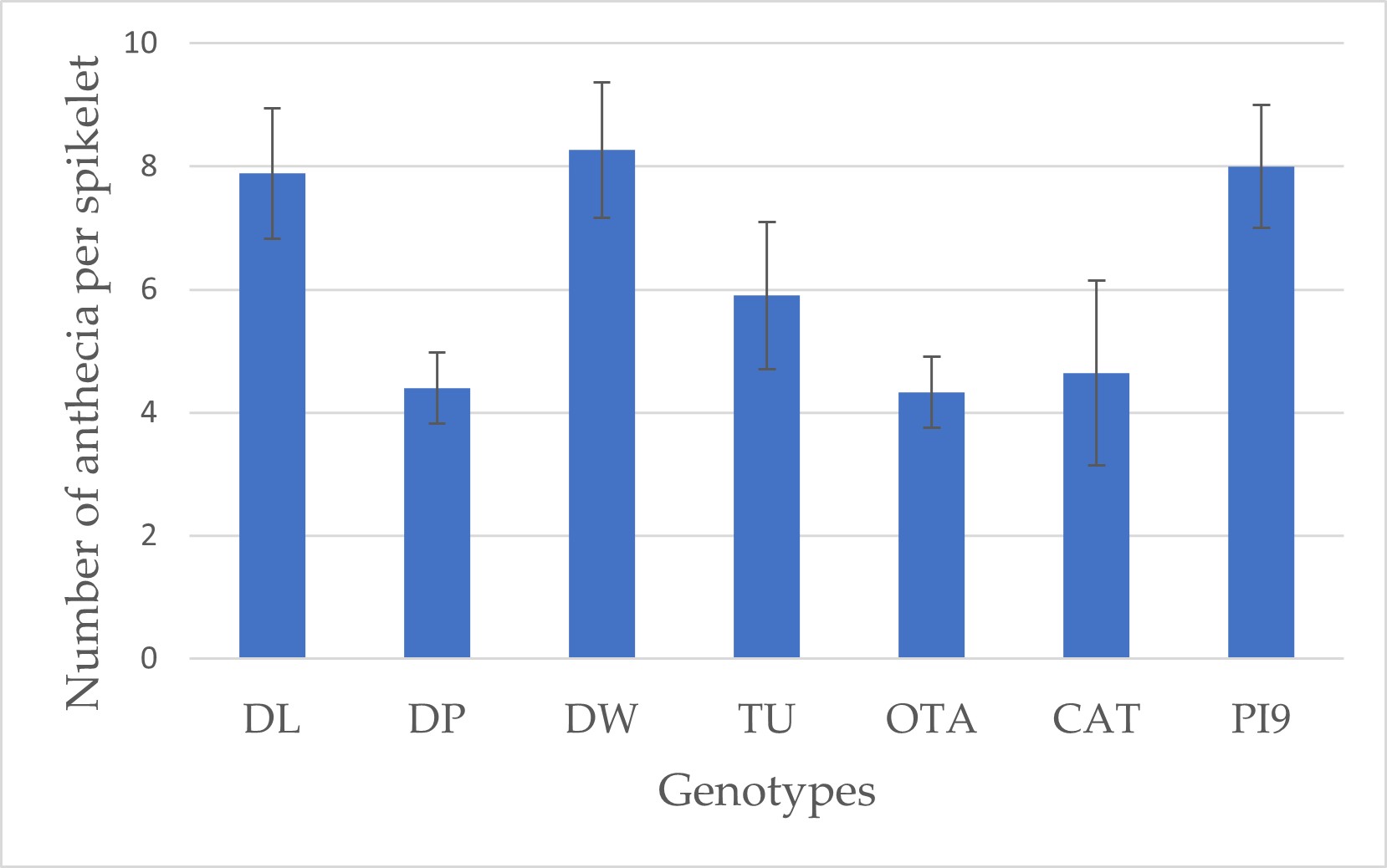

Supplement: Supplementary file 1 [file plants-15-01050-s001.zip › supplementary material/Figure S2. Anthecia per spikelet.jpg]

***Ovary lenght - Stage I***

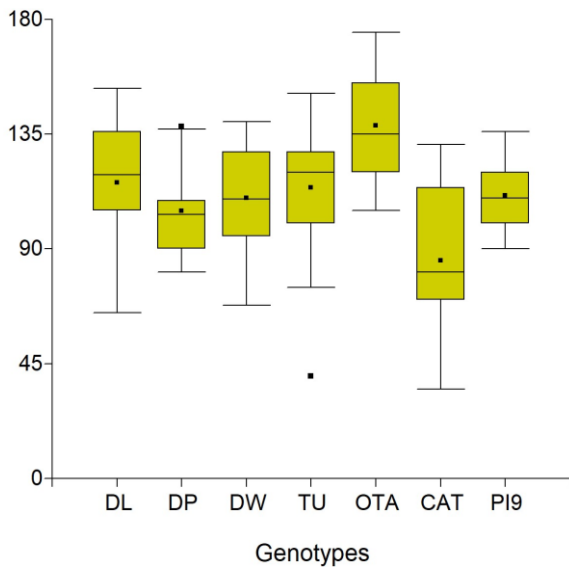

***Ovary lenght - Stage II***

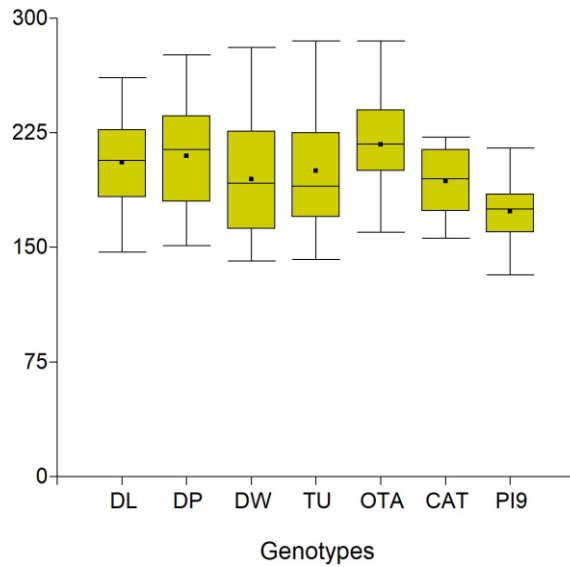

***Ovary lenght - Stage III***

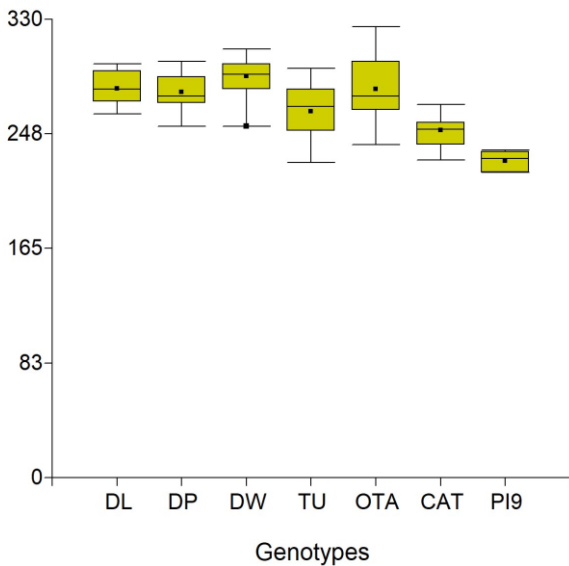

***Ovary lenght - Stage IV***

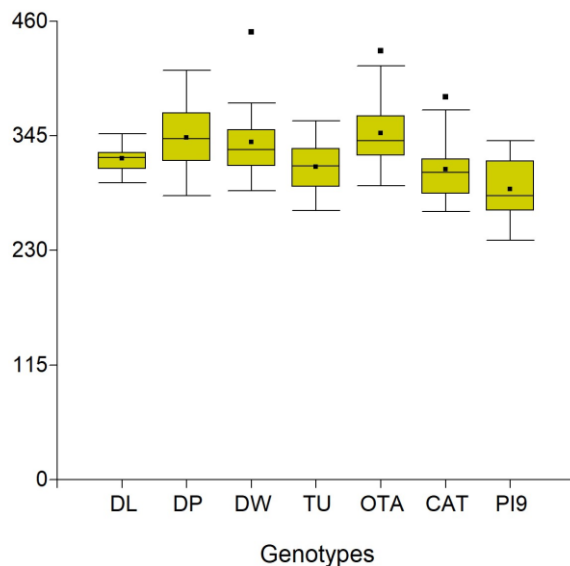

Supplement: Supplementary file 1 [file plants-15-01050-s001.zip › supplementary material/Figure S7. OL Stages.pdf]

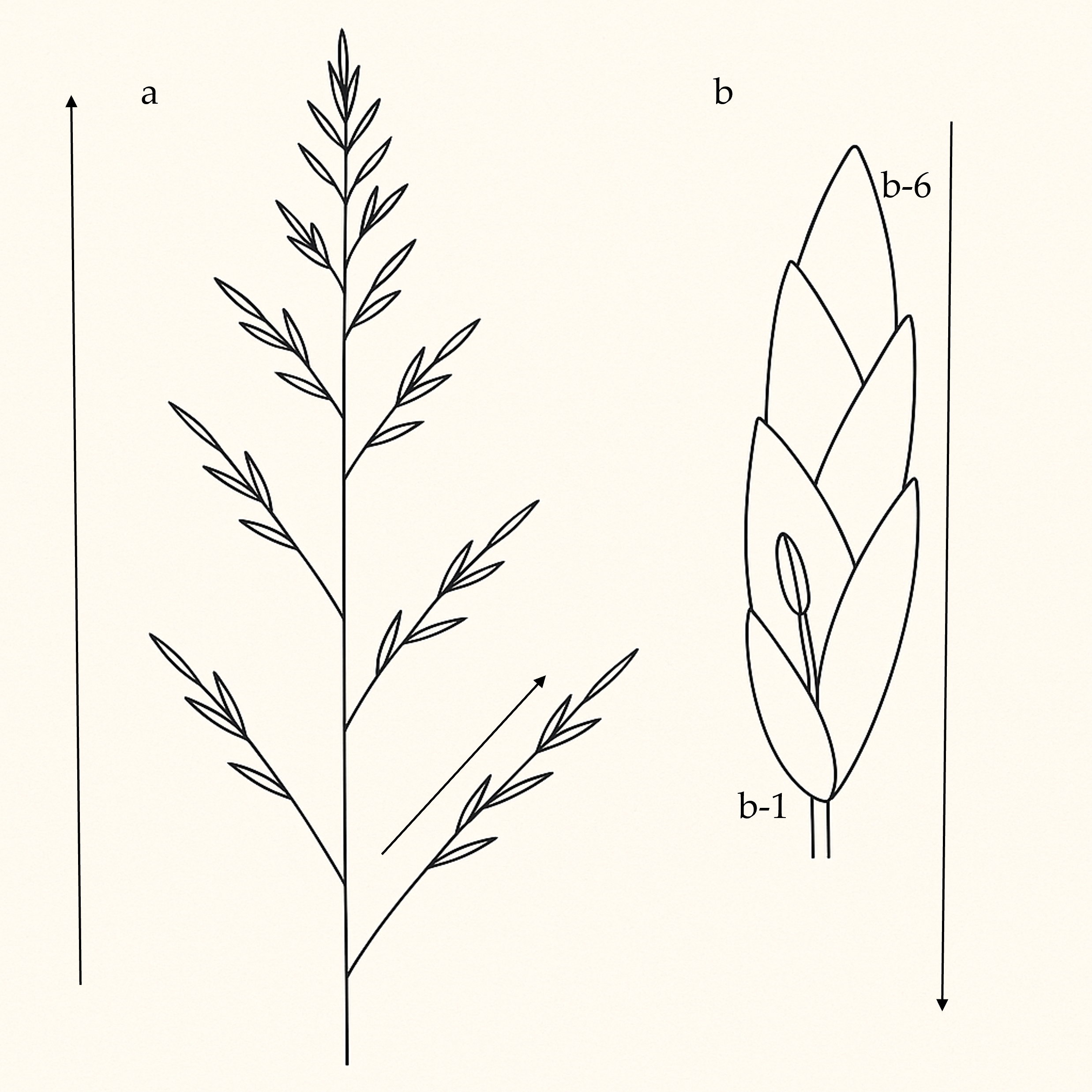

Supplement: Supplementary file 1 [file plants-15-01050-s001.zip › supplementary material/Figure S1. Panicle and spikelet scheme.png]

***Style lenght - Stage I***

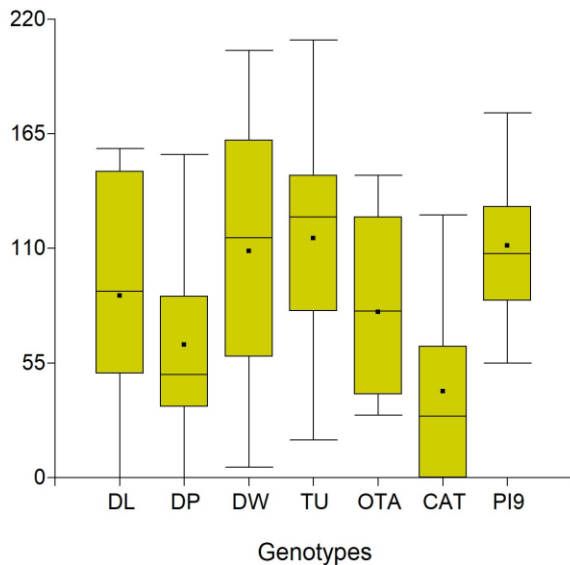

***Style lenght - Stage II***

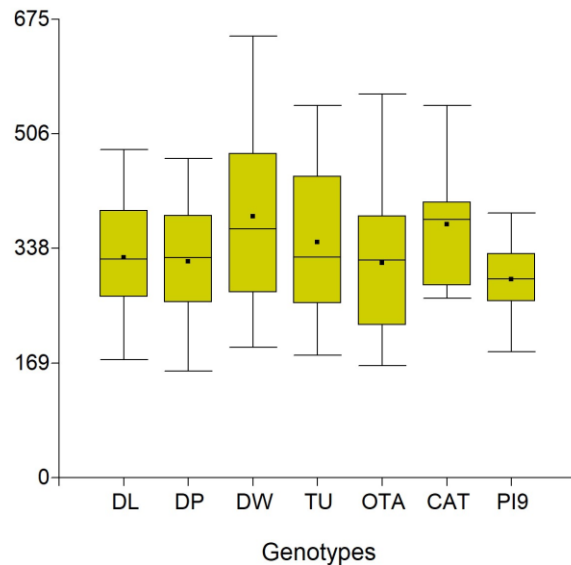

***Style lenght - Stage III***

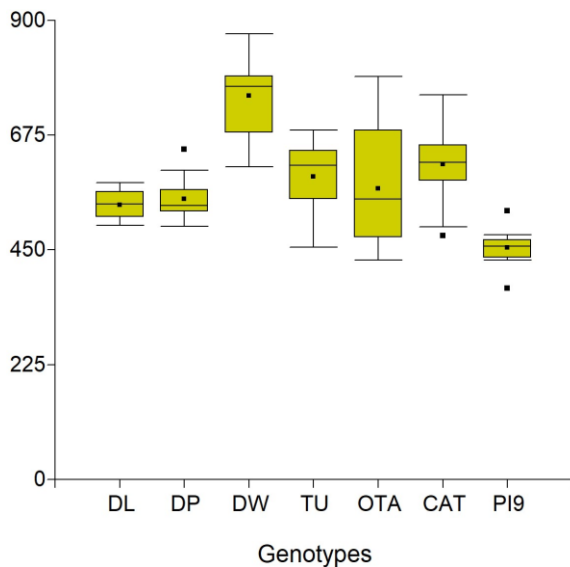

***Style lenght - Stage VI***

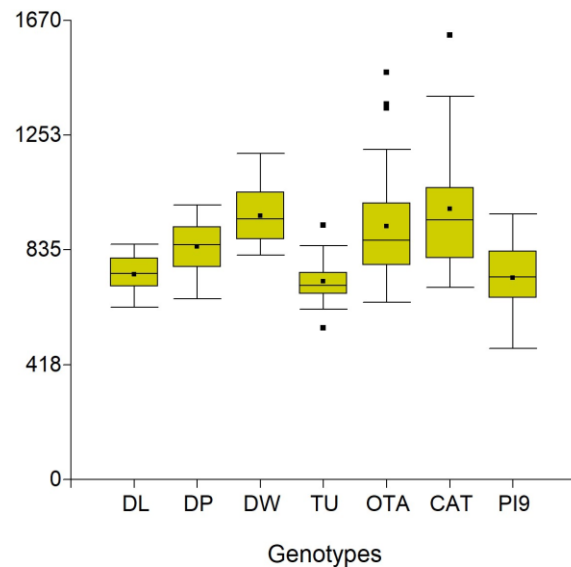

Supplement: Supplementary file 1 [file plants-15-01050-s001.zip › supplementary material/Figure S8. SL Stages.pdf]

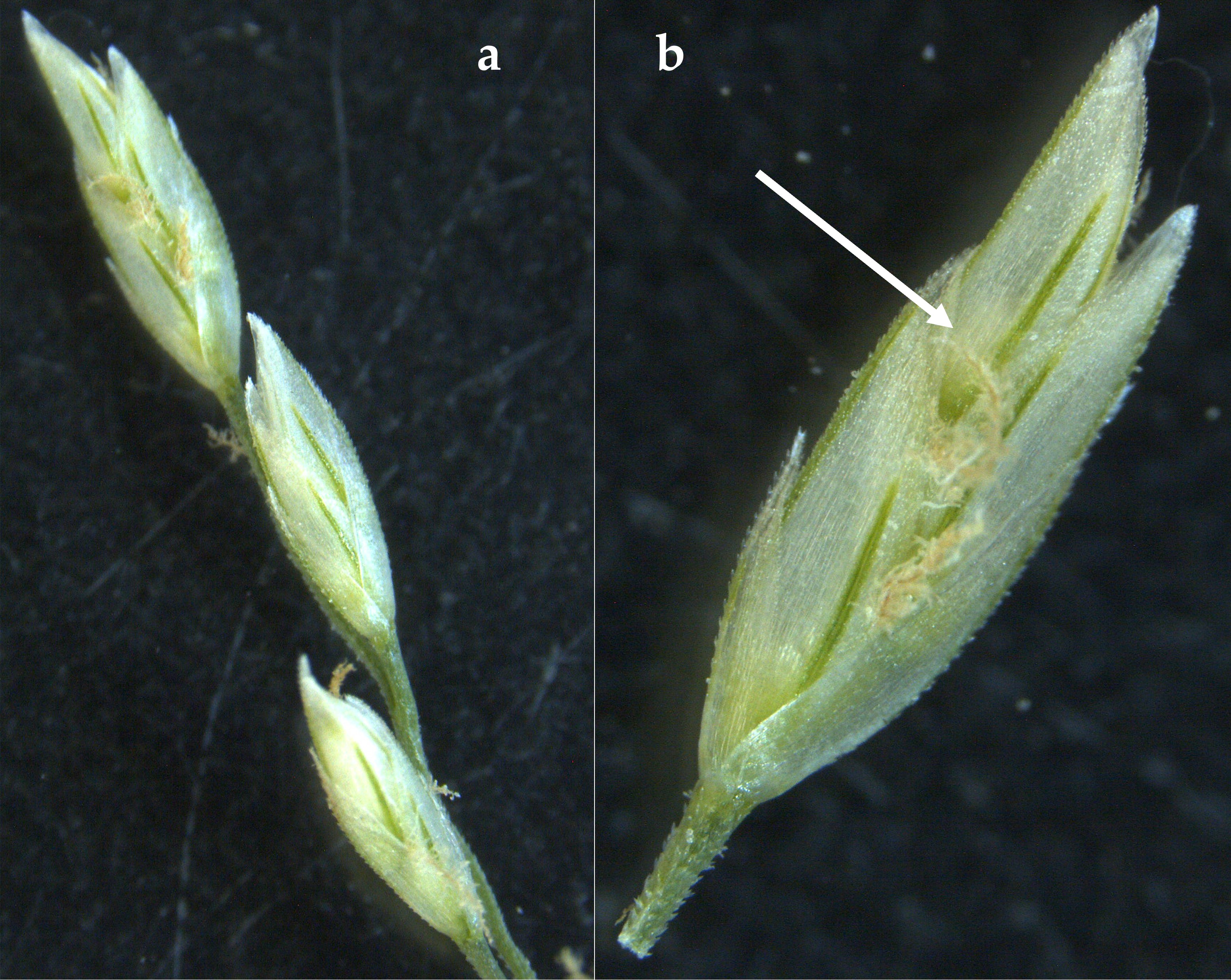

Supplement: Supplementary file 1 [file plants-15-01050-s001.zip › supplementary material/Figure S3. OTA splikelet.jpg]

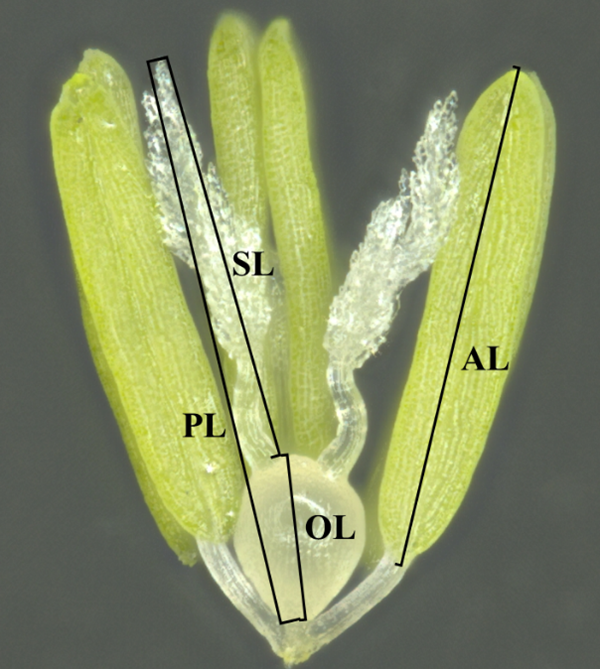

Supplement: Supplementary file 1 [file plants-15-01050-s001.zip › supplementary material/Figure S10. Parameters.png]

***Anther lenght - Stage I***

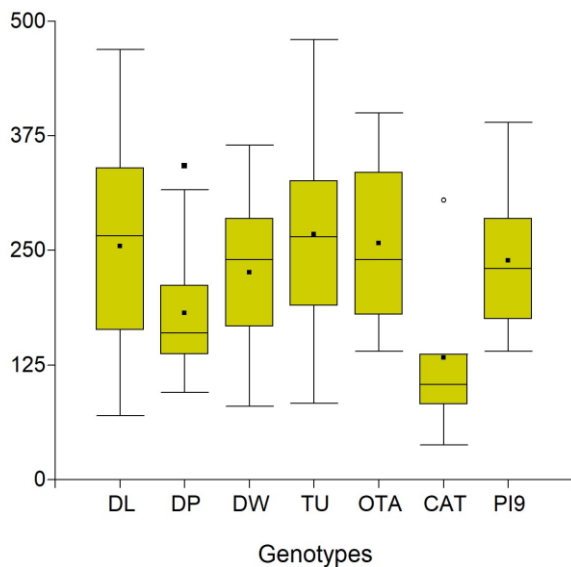

***Anther lenght - Stage II***

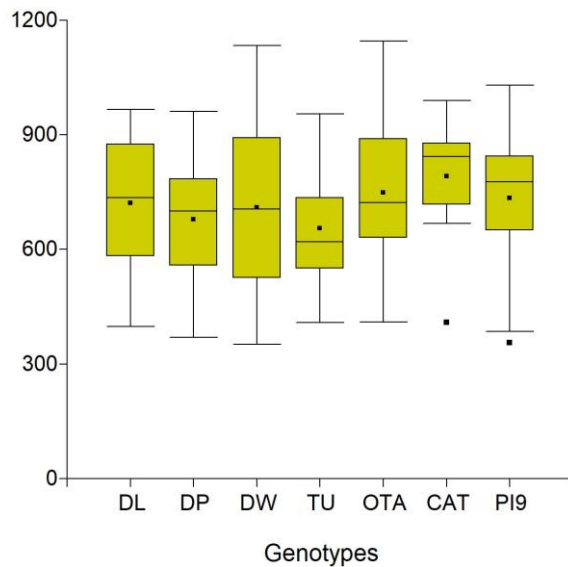

***Anther lenght - Stage III***

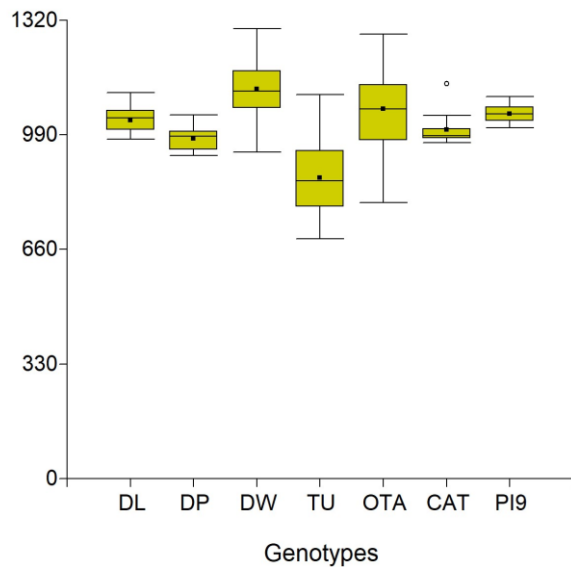

***Anther lenght - Stage VI***

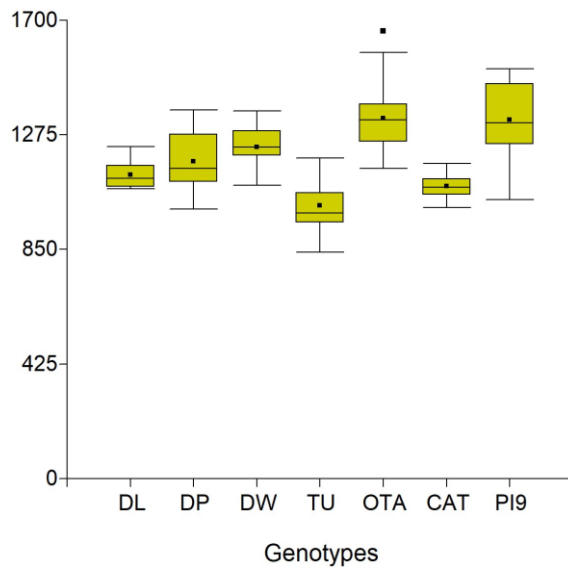

Supplement: Supplementary file 1 [file plants-15-01050-s001.zip › supplementary material/Figure S9. AL Stages.pdf]
